# From laboratory to clinic: opportunities and challenges of functional food active ingredients in cancer therapy

**DOI:** 10.3389/fnut.2025.1627949

**Published:** 2025-07-30

**Authors:** Nie Zhang, Yanzhi Ren, Yahui Xu

**Affiliations:** ^1^Department of Oncology, Graduate School of Anhui Medical University, Hefei, China; ^2^Key Laboratory of Gametes and Abnormal Reproductive Tract of National Health Commission, Anhui Medical University, Hefei, China; ^3^Department of Cardiology, Shizhong District People's Hospital, Zao Zhuang, China; ^4^Department of Anesthesiology, The First Affiliated Hospital of Anhui Medical University, Hefei, China

**Keywords:** functional food, bioactive compounds, cancer therapy, nanodelivery systems, precision nutrition

## Abstract

This review provides a comprehensive analysis of the potential of functional food active ingredients in cancer prevention and therapy. It outlines the multifaceted anticancer mechanisms of bioactive compounds—such as polyphenols, carotenoids, omega-3 fatty acids, phytosterols, alkaloids, isothiocyanates, polysaccharides, phenolic acids, flavonols, and amide-bearing compounds—which include antioxidant and anti-inflammatory activities, induction of apoptosis and autophagy, modulation of the tumor microenvironment, interference with cell cycle regulation and signaling pathways, and regulation of cancer-related microRNA expression. The review further discusses the synergistic effects of these compounds when combined with conventional treatments like radiotherapy and chemotherapy, highlighting their role in enhancing efficacy and mitigating side effects. Despite promising preclinical data, challenges such as poor bioavailability, dose-dependent safety concerns, and the need for large-scale randomized clinical trials and regulatory standardization remain. Proposed future directions include advanced nanodelivery systems, eutectic technologies, and precision nutrition strategies, which together could accelerate the translation of these natural compounds from the laboratory to clinical application. Ultimately, the integration of functional food active ingredients into comprehensive cancer care may offer novel, safer, and more personalized approaches to oncologic treatment and prevention.

## 1 Introduction

**Figure 1:**
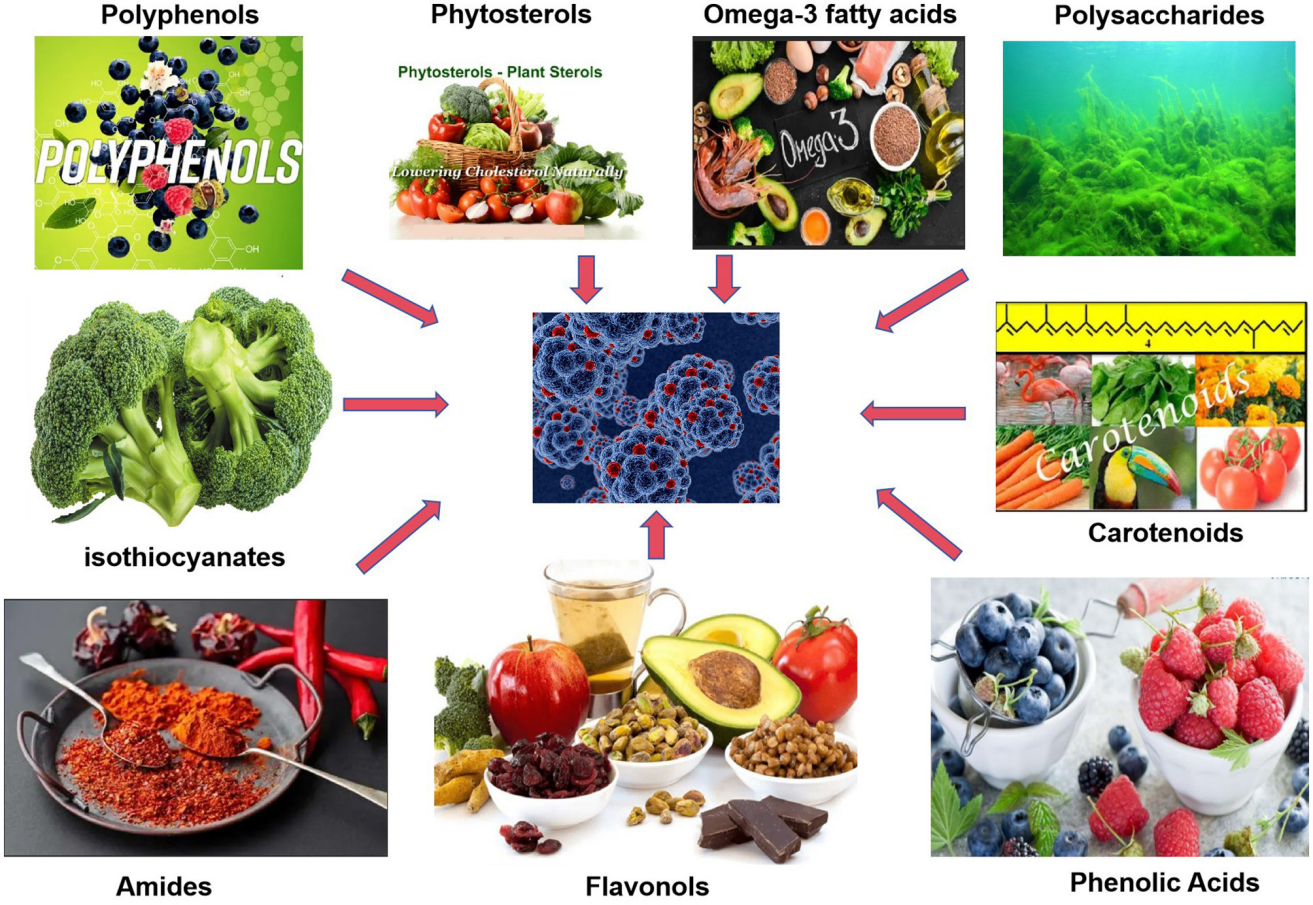
Schematic diagram of anticancer mechanisms of functional food active ingredients. Created with figdraw.com.

Functional foods are usually defined as foods that have health-promoting and disease-preventing effects in addition to providing basic nutrition. Unlike conventional foods, functional foods are enriched with certain bioactive ingredients, either naturally present in the food or added through fortification. They confer health benefits that go “beyond basic nutrition”, such as antioxidant, immunomodulatory, or disease risk reduction effects (1). Common functional food active ingredients include plant-derived polyphenolic compounds, carotenoids, omega-3 polyunsaturated fatty acids, as well as dietary fiber, prebiotics, and probiotics (2). These active ingredients can play a variety of biological roles in the body, making functional foods more beneficial to health compared to ordinary foods (Figure 1).

Cancer is a major social, public health, and economic issue of the 21st century, accounting for almost one in six (16.8%) deaths worldwide and one in four (22.8%) deaths from non-communicable diseases. According to the latest statistics from the International Agency for Research on Cancer, the estimated number of new cancer cases worldwide in 2020 was around 20 million, with nearly 10 million deaths due to cancer. As the population ages and grows, the burden of cancer continues to increase, with the number of new cancer cases globally projected to rise to about 35 million per year by 2050 (3). Such a grim epidemiologic picture has prompted increased attention to modifiable cancer-causing factors, among which dietary factors are particularly important.

Numerous studies have shown that dietary structure plays a key role in the development of cancer. Healthy dietary patterns are associated with a lower risk of cancer, while diets high in fat, red meat, and processed meats increase the risk of certain cancers. It is estimated that maintaining a proper diet could prevent about 30–50% of cancers (4). Epidemiological investigations have confirmed that increased intake of fruits and vegetables reduces the risk of many types of cancer, including colorectal, prostate, breast, and gastric cancer (5–7). Additionally, increased intake of foods rich in omega-3 fatty acids, such as deep-sea fish and nuts, can help reduce the risk of cancer development (8). Accordingly, the Mediterranean diet, which is based on plant foods, is considered to have significant cancer-preventive benefits due to its richness in functional components, such as antioxidants and anti-inflammatory properties, which help inhibit the proliferation of tumor cells, induce apoptosis, and reduce cancer cell invasion and angiogenesis, among other effects (4). Epidemiologic evidence suggests that adherence to the Mediterranean diet is strongly associated with a reduction in the incidence of and mortality from many types of cancer, including colorectal and breast cancer (9–11). These studies demonstrate the great potential of dietary interventions in cancer prevention.

**Figure 2:**
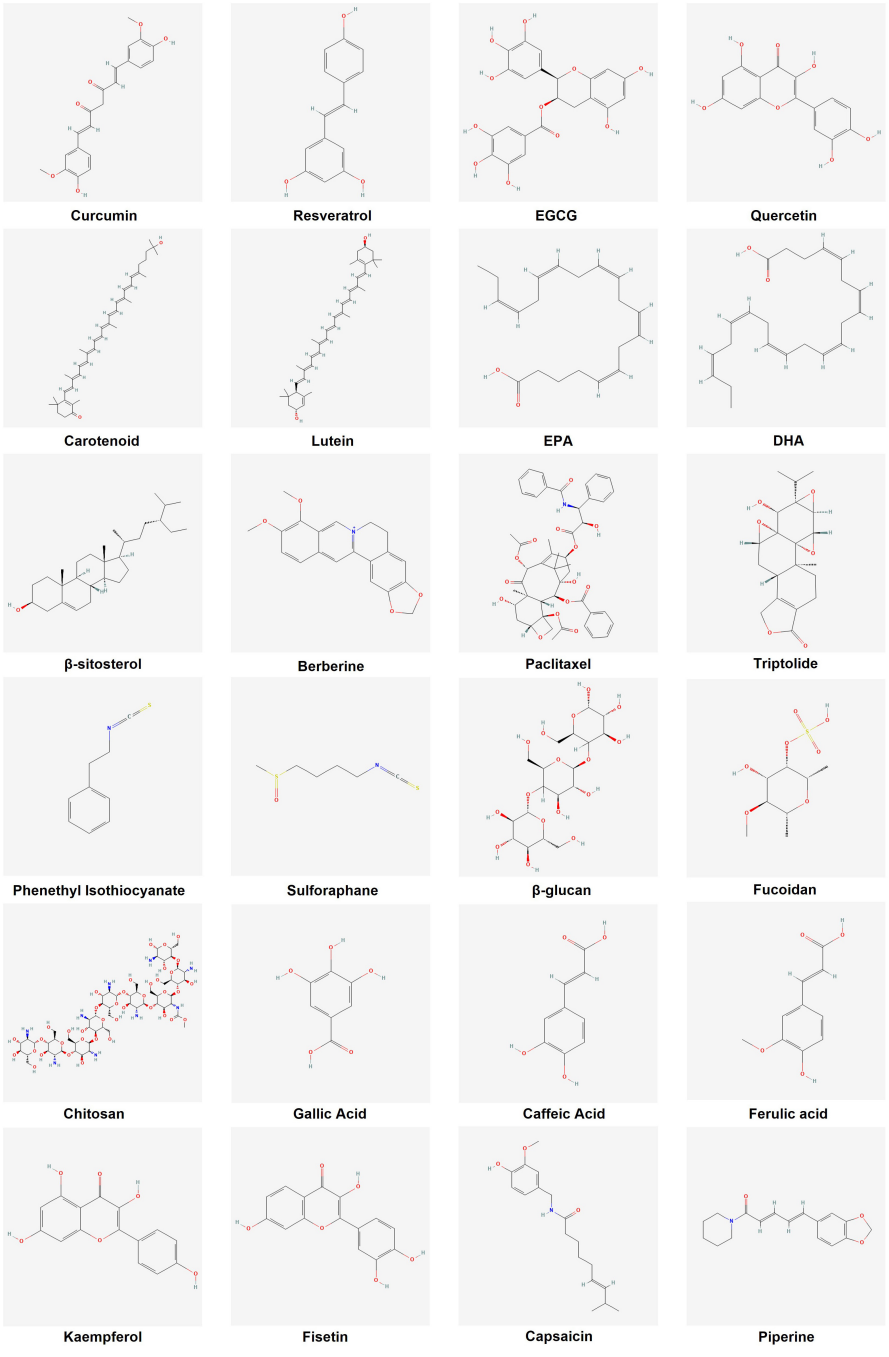
Chemical structures of representative dietary anticancer bioactives.

In view of the important role and great potential of functional food active ingredients in cancer prevention and treatment, this paper will provide an overview of recent advances in related fields. First, we will describe the mechanisms of action of the major active ingredients in functional foods in preventing and suppressing tumors, including their intervention in cancer development through antioxidant, anti-inflammatory, apoptosis-inducing, and tumor proliferation and metastasis-inhibiting pathways (Figure 2). Second, we will explore the potential value of these active ingredients in clinical applications, such as their use as dietary supplements for cancer prevention or as adjuvant therapy in conjunction with conventional therapies, and analyze the current challenges. These challenges include the limited stability and bioavailability of bioactive ingredients *in vivo*, complex mechanisms of action, inadequate dosage and safety evaluations, and regulatory barriers. Finally, we emphasize the need for further studies to address these issues, provide insight into the mechanisms of action of functional food active ingredients, and validate their clinical efficacy, with a view to better translating them into effective strategies for cancer prevention and treatment. Through this review, we hope to provide a comprehensive understanding of the current status of research on functional food active ingredients in the field of tumor prevention and treatment and to guide future research and applications.

## 2 Methods

We conducted a comprehensive literature search to gather relevant studies and evidence on functional food-derived bioactive compounds in cancer therapy. The search was performed in multiple databases, including PubMed, Web of Science, and Embase, and was supplemented by manual searches using Google Scholar to ensure completeness. Search terms included combinations of keywords such as “bioactive compounds”, “functional food ingredients”, “cancer prevention”, “cancer therapy”, “polyphenols”, “carotenoids”, “omega-3 fatty acids”, “phytosterols”, “alkaloids”, “isothiocyanates”, “miRNA”, and “nanodelivery”. Only English-language publications were considered. Searches were limited to publications in English and primarily to the last 5 years (2020–2024) to capture the most recent advances.

### 2.1 Inclusion criteria

We defined strict inclusion criteria to select which functional food active ingredients and studies to include in this review. Only naturally derived compounds found in foods were considered, in line with the definition of functional food ingredients. Each included compound was required to have documented anticancer mechanisms of action supported by scientific studies. We further required that each bioactive compound's antitumor potential be supported by recent preclinical or clinical evidence, preferably from studies published within the past 5 years, including *in vivo* animal models or clinical trials demonstrating its efficacy or elucidating underlying mechanisms. Priority was given to higher levels of evidence; whenever available, systematic reviews, meta-analyses, and large-scale randomized clinical trials were included to provide comprehensive support. Original research articles were included if they offered novel mechanistic insights or translational data on a qualifying compound. All included studies had to be published in English-language, peer-reviewed journals.

### 2.2 Exclusion criteria

We excluded any articles or compounds that did not meet the above standards. Synthetic chemicals or isolated nutrients that were not derived from natural food sources were outside the scope of this review. We also excluded studies that lacked a clear link between the functional ingredient and cancer mechanisms—for instance, purely observational epidemiological studies reporting correlations between diet and cancer outcomes without investigating any biological mechanism were not included. Similarly, small-scale or non-generalizable animal experiments were excluded if they failed to provide substantial mechanistic insights. In terms of publication type, non-peer-reviewed materials such as conference abstracts, correspondence, and editorials were not considered. Case reports and anecdotal observations lacking broader scientific relevance were also excluded.

### 2.3 Study selection and data extraction

After completing the initial database searches, all retrieved records were screened by title and abstract to identify potentially relevant studies, followed by a full-text review to determine final eligibility based on the predefined inclusion and exclusion criteria. Two independent reviewers conducted the selection process to ensure consistency, with any discrepancies resolved through discussion. From each included study, we extracted detailed information on the type and source of the bioactive compound, targeted cancer types, experimental models used (*in vitro, in vivo*, or clinical), elucidated anticancer mechanisms, and any reported clinical efficacy or safety outcomes. Emphasis was placed on studies that clearly delineated molecular pathways or therapeutic effects, with priority given to those with translational relevance. In synthesizing the data, we organized the findings according to the major classes of functional food compounds, highlighting their mechanistic diversity, therapeutic promise, and key challenges in clinical application.

## 3 Anticancer mechanisms of functional food active ingredients

### 3.1 Anticancer mechanisms of polyphenols

#### 3.1.1 Antioxidant and anti-inflammatory effects

Polyphenolic compounds exhibit significant antioxidant and anti-inflammatory activities, scavenging excess free radicals and reducing cellular damage caused by oxidative stress (12). By boosting intracellular antioxidant enzyme levels and directly scavenging reactive oxygen species (ROS), polyphenols protect DNA from oxidative damage, thereby reducing the risk of mutation and carcinogenesis. Additionally, polyphenols can inhibit signaling pathways associated with chronic inflammation, such as nuclear factor-κB (NF-κB), thereby decreasing the production of pro-inflammatory mediators. Studies have shown that polyphenols, such as curcumin, can block NF-κB activation and downregulate the expression of pro-inflammatory genes, including interleukin-6 and cyclooxygenase-2 (13). By reducing the levels of these pro-tumor inflammatory factors, polyphenols lessen the constant irritation caused by inflammation on tissues, thereby helping to reduce the risk of cancer development and progression.

#### 3.1.2 Apoptosis and autophagy regulation

Polyphenols induce programmed cell death in cancer cells, mainly via mitochondria-mediated endogenous pathways. They tend to upregulate the pro-apoptotic protein Bax and inhibit the anti-apoptotic protein Bcl-2, altering the Bax/Bcl-2 ratio to promote the release of cytochrome c, which in turn activates downstream apoptosis-executing proteins such as caspase-3 and caspase-9 (14, 15). Curcumin has been shown to downregulate Bcl-2 family proteins by inhibiting pathways such as NF-κB, triggering the cysteine asparaginase cascade in cancer cells to trigger apoptosis (12). Polyphenols such as resveratrol likewise enhance apoptotic signaling, making cancer cells more susceptible to apoptosis. In addition to inducing apoptosis, polyphenols also affect the regulation of autophagy, a cellular survival/death process. Many polyphenols initiate the autophagy program by inhibiting the PI3K/Akt/mTOR signaling pathway or activating energy-sensing pathways such as AMPK (16). This implies that polyphenols can disrupt the inhibition of autophagy by mTOR and induce autophagic cell death or autophagy-mediated survival stress relief in cancer cells. Experimental evidence showed that resveratrol treatment upregulated the expression of the autophagy marker protein Beclin-1 and increased the LC3-II/LC3-I ratio, demonstrating that it induced autophagy in cancer cells while also promoting apoptosis (14).

#### 3.1.3 Inhibition of tumor microenvironment and angiogenesis

Polyphenols also exert anticancer effects by remodeling the tumor microenvironment. They can intervene in the function of peritumoral support cells, thereby weakening the tumor's growth environment. Studies have shown that resveratrol treatment reduces the proportion of tumor-promoting M2 tumor-associated macrophages (TAM) in tumor tissues and significantly enhances the activation of tumor-infiltrating CD8+ T cells (17). In addition, resveratrol can effectively eliminate senescent cancer-associated fibroblasts (CAF) in tumor tissues and diminish the supportive effect of CAF on tumor cell proliferation and invasion, thus inhibiting the progression of pancreatic cancer (18). In terms of tumor angiogenesis, polyphenols inhibit neovascularization by blocking pro-angiogenic signals. Specific mechanisms include downregulation of pro-angiogenic factors such as vascular endothelial growth factor (VEGF) and hypoxia-inducible factor-1α (HIF-1α) expression (15). Taking tea polyphenol epigallocatechin gallate (EGCG) as an example, it can significantly reduce the levels of VEGF-A and HIF-1α secreted by tumor cells and inhibit tumor angiogenesis and nutrient supply, thus effectively hindering tumor growth and metastasis (15).

Polyphenols exert multitargeted anticancer effects through modulation of key signaling pathways. The green tea catechin EGCG can inhibit multiple pro-tumor signaling cascades. It has been shown to reduce the activation of the MAPK/ERK and PI3K/Akt pathways, upregulate the cyclin-dependent kinase inhibitor p21, and trigger mitochondrial apoptosis by downregulating the anti-apoptotic proteins Bcl-2/Bcl-xL (19–21). EGCG also attenuates cancer cell invasion and metastasis by lowering matrix metalloproteinases (MMPs) and suppressing angiogenic factors (22). Another well-studied polyphenol, quercetin, induces apoptosis via the PI3K/Akt/mTOR axis. In hepatocellular carcinoma cells, quercetin treatment downregulated proline 4-hydroxylase (P4HA2) and inhibited the PI3K/Akt/mTOR signaling pathway, leading to pronounced apoptosis (23). This highlights how polyphenols can interfere with survival pathways and activate intrinsic death programs.

#### 3.1.4 Polyphenols: precision miRNA targeting for cancer suppression

Curcumin emerges as a potent miRNA modulator with specific targeting of oncogenic and tumor suppressor miRNAs. Recent studies demonstrate that curcumin significantly downregulates miR-21 (oncomiR suppression) while upregulating miR-34a/b/c by two- to four-fold in colorectal cancer cells (24). The compound activates tumor suppressor miRNAs through ROS/NRF2 pathway activation, independent of p53 status, and modulates miRNA promoters through direct transcription factor binding (24). Clinical evidence from 2023 shows that a curcumin nanomicelle formulation significantly reduces miR-155 (*p* = 0.002), miR-138 (*p* = 0.024), and miR-16 (*p* = 0.0001) in human subjects, demonstrating systemic miRNA modulation (25). In colorectal cancer models, curcumin treatment reduced IC50 values from 40 ± 4.2 μM to 5 ± 0.36 μM when combined with 5-FU, with miR-34a expression increasing 2.5-fold in HCT-116 cells (24).

**Figure 3:**
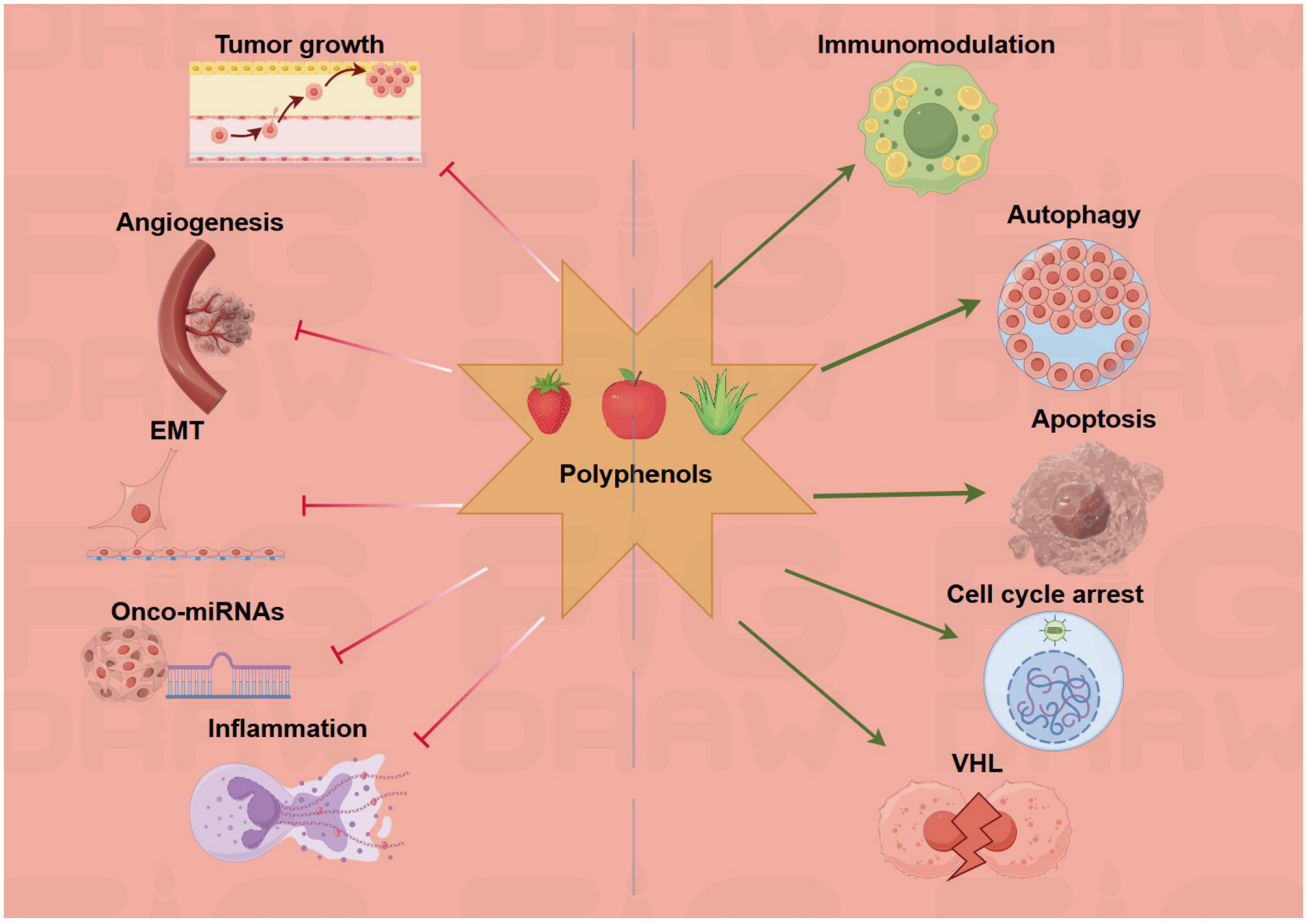
Polyphenols target multiple hallmarks of cancer. The green arrows refer to promotion or increase, whereas the red hammerhead lines refer to inhibition. Created with figdraw.com.

Resveratrol targets the miR-200 family and miR-125b-5p for epithelial–mesenchymal transition (EMT) inhibition. The compound upregulates miR-200c-3p and miR-125b-5p by two- or three-fold, suppressing mesenchymal markers and inducing apoptosis through the modulation of the Bcl-2/Bax ratio modulation (26). A breakthrough 2022 study using genetically engineered mice showed that resveratrol prevents 60% of tumors and achieves 33% complete remission through miR-96 upregulation, which inhibits KRAS translation (27). EGCG demonstrates unique miRNA targeting through HIF-1α stabilization. The compound binds directly to HIF-1α (Kd = 3.47 μM), upregulating miR-210 and enhancing miR-155-5p expression by 2.12 ± 0.02-fold in HCT-116 cells (28). This miRNA modulation reverses chemoresistance by targeting MDR1, reducing the 5-FU IC50 from 150 ± 6.4 μM to 11 ± 0.96 μM in DLD1 cells (29). Quercetin affects 105 miRNAs in pancreatic cancer (80 upregulated, 25 downregulated), with particular emphasis on the downregulation of miR-200b-3p, which reduces cancer stem cell aggressiveness. The compound targets Notch signaling through miRNA-mediated regulation of the Notch/Numbl pathway, while miR-217 upregulation reduces cisplatin resistance in ovarian cancer models (30) (Figure 3).

### 3.2 Anticancer mechanisms of carotenoids

#### 3.2.1 Pro-oxidant and antioxidant balance

Carotenoids are a class of strong antioxidants that scavenge free radicals and reduce DNA damage from oxidative stress, thereby preventing genetic mutations and cancerous lesions (31, 32). However, under certain conditions (e.g., high oxygen partial pressure, high doses, or environments with high levels of ROS already present in tumor cells), carotenoids can be converted from antioxidants to pro-oxidants, generating excess ROS and triggering oxidative stress that induces cancer cell death (32). This “dual” effect allows carotenoids to play a protective role in normal cells while triggering apoptosis in cancer cells by increasing their ROS levels, resulting in a selective killing effect on tumors. In other words, carotenoids optimize the redox balance of normal cells while enhancing oxidative stress in tumor cells, thus inhibiting tumor growth (32).

#### 3.2.2 Immunomodulatory effect

Carotenoids also exert anticancer effects through immunomodulation. Studies have shown that these compounds can affect the function of various immune cells, such as enhancing the activity of T lymphocytes and natural killer cells (NK cells), and regulating macrophage function, thereby enhancing the overall antitumor immune response of the body (31, 33). In addition, carotenoids have significant anti-inflammatory effects, inhibiting chronic inflammatory responses in the tumor microenvironment. Lycopene downregulates the production of pro-inflammatory cytokines and decreases the expression of inflammatory mediators, such as inducible nitric oxide synthase (iNOS) and COX-2, through the inhibition of the NF-κB signaling pathway (34, 35). This inhibition of pro-inflammatory signaling helps to weaken the pro-growth inflammatory environment of tumors, reduces the opportunities for tumor cells to evade immune surveillance through inflammation, and enhances the effectiveness of the antitumor immune response. As a result, carotenoids synergistically inhibit tumor development by creating a microenvironment more conducive to the immune system's recognition and elimination of cancer cells (36).

#### 3.2.3 Cell cycle regulation and DNA repair

Carotenoids also inhibit tumor growth by regulating the cell cycle and promoting DNA damage repair. Many studies have found that carotenoids can affect the expression of cell cycle-related proteins, such as increasing the levels of the cell cycle inhibitors p21Cip1 and p27Kip1, while inhibiting the activity of Cyclin and its related kinases, thereby inducing cell cycle arrest in cancer cells (37, 38). β-Carotene treatment arrests a variety of cancer cells in the G0/G1 or G2/M phase: On the one hand, it upregulates CDK inhibitors such as p21 and decreases Cyclin A levels, and on the other hand, it increases intracellular p27Kip1 levels by inhibiting the expression of Skp2 (the protein that drives p27 degradation). Both pathways effectively prevent the continued proliferation of cancer cells (39–41).

In addition to inhibiting proliferation, carotenoids affect the cellular DNA damage response pathway, enhancing p53-mediated gene monitoring and repair. Activated p53 triggers cell cycle arrest to provide damaged DNA with enough time to repair or, in the case of excessive damage, to initiate apoptosis, thus preventing the proliferation of cells carrying severe genetic defects (42, 43). Certain carotenoids can upregulate p53 and its downstream repair genes to improve DNA repair efficiency. For example, fucoxanthin was found to induce a G0/G1 phase block in cancer cells while upregulating GADD45α, a DNA repair gene regulated by p53, which helps maintain genomic stability and reduce cancer risk (44). Through these mechanisms, carotenoids can reduce the accumulation of DNA damage and the occurrence of gene mutations, playing an anticancer role at the molecular level.

Carotenoids likewise impact critical molecular pathways in cancer cells. Lycopene, a carotenoid found in tomatoes, is known to suppress chronic inflammatory signaling; notably, it inhibits NF-κB activation, thereby reducing pro-inflammatory cytokines and mediators such as COX-2 and iNOS in the tumor microenvironment. This attenuation of NF-κB leads to decreased tumor-promoting inflammation and can impair angiogenesis signals, hindering tumor growth. Lutein, another dietary carotenoid, directly influences cell survival pathways. In lung cancer models, lutein was found to induce apoptosis by modulating PI3K/Akt signaling, effectively inhibiting Akt to trigger cancer cell death while sparing normal cells (45). Moreover, lutein can act as a pro-oxidant under tumor conditions: in gastric cancer cells, lutein elevated intracellular ROS via NADPH oxidase, which unexpectedly led to NF-κB–mediated upregulation of pro-apoptotic factors and caspase-3 activation, culminating in apoptosis (46). These examples illustrate that carotenoids can both protect normal cells and selectively kill cancer cells by shifting redox and signaling balances toward cell cycle arrest and apoptosis.

#### 3.2.4 Carotenoids: oxidative stress and miRNA networks

Lycopene modulates oxidative stress-responsive miRNAs in prostate cancer through antioxidant pathways. Clinical studies demonstrate that circulating lycopene increases with supplementation (pooled mean difference: 0.1361; 95% CI [0.0574; 0.2148]), correlating with a 7% reduction in specific prostate cancer types. The compound induces apoptosis and cell cycle arrest through miRNA-mediated regulation of apoptotic proteins at effective concentrations of 5–25 μM (47). β-Carotene affects cancer pathways through Pin1 inhibition and PI3K/AKT signaling suppression; however, clinical studies reveal an increased lung cancer risk in smokers (RR: 1.19; 95% CI: 1.08–1.32), emphasizing the importance of personalized approaches based on individual risk factors (48).

#### 3.2.5 Advances in potential clinical applications

In terms of clinical translation, carotenoids are being investigated for cancer prevention and adjuvant therapy. Epidemiologic studies have shown that high fruit and vegetable intake is associated with a reduced risk of certain cancers, but high doses of carotenoid supplements may be counterproductive in specific populations (31). Therefore, one of the current research priorities is to clarify the optimal dosage of different carotenoids and the applicable populations to safely and effectively exert their anticancer preventive effects. Meanwhile, the potential of carotenoids as chemotherapeutic adjuvants has also received attention. It has been proposed to combine carotenoids with certain chemotherapeutic drugs: carotenoids play an antioxidant protective role in normal tissues to alleviate the side effects of chemotherapy while exhibiting pro-oxidant effects in tumor tissues to enhance the killing of cancer cells (49). These recent advances suggest that the rational utilization of the multiple mechanisms of antioxidant and immune enhancement of carotenoids is expected to improve the efficacy of cancer prevention and treatment, but more clinical trials are needed to validate their safety and efficacy.

### 3.3 Anticancer mechanisms of omega-3 fatty acids

#### 3.3.1 Regulation of inflammatory pathways

Eicosapentaenoic acid (EPA) and docosahexaenoic acid (DHA) reduce the levels of pro-inflammatory cytokines and downregulate inflammatory mediators such as COX-2, TNF-α, NF-κB, and prostaglandin E2 (PGE_2_), which attenuates cancer-related inflammatory responses (50, 51). Meanwhile, DHA and EPA exhibit anticancer effects by inducing apoptosis and inhibiting proliferation and angiogenesis. Omega-3 polyunsaturated fatty acids have significant anti-inflammatory effects and may reduce the inflammatory response in the tumor microenvironment through several mechanisms. EPA and DHA can competitively replace arachidonic acid in the cyclooxygenase pathway, reducing the synthesis of pro-inflammatory mediators such as PGE_2_ and leukotrienes (52). In addition, omega-3-derived bioactive products promote inflammation resolution and help to end chronic inflammation (53). At the level of signaling pathways, omega-3 inhibits the activation of the pro-inflammatory transcription factor NF-κB and prevents its translocation into the nucleus, thereby downregulating the expression of a series of pro-inflammatory genes. Cellular experiments have shown that EPA treatment blocked the entry of NF-κB p65 into the nucleus and reduced its activity (51). Meanwhile, DHA reduces the expression level of the COX-2 enzyme in cancer cells and decreases oncogenic inflammatory signaling mediated by COX-2 (54).

Together, these effects reduce tumor-associated pro-inflammatory cytokine release and inflammatory cascade responses, thereby helping to inhibit inflammation-driven tumor progression. Clinical studies also support the anti-inflammatory benefits of omega-3. Omega-3 supplementation in cancer patients reduces inflammatory markers such as IL-6 and C-reactive protein (CRP) and improves inflammation-based prognostic scores (55). In conclusion, omega-3 fatty acids alleviate cancer-associated chronic inflammation and facilitate antitumor therapy by inhibiting NF-κB and reducing COX-2 and pro-inflammatory mediator production.

#### 3.3.2 Affects cell membrane structure and signaling

Omega-3 fatty acids can be incorporated into the phospholipid bilayer of cell membranes, significantly altering the fatty acid composition and biophysical properties of the membranes. This incorporation decreases the proportion of ω-6 fatty acids, such as arachidonic acid, and increases the content of ω-3 fatty acids, thereby improving membrane fluidity and flexibility (56). Changes in membrane structure affect the composition and stability of membrane microdomains, which in turn regulate membrane receptor aggregation and signaling (57). It has been shown that DHA/EPA-enriched membranes can disrupt the lipid raft structure in cancer cell membranes, leading to the depolymerization of the epidermal growth factor receptor (EGFR) from the lipid rafts and impairing the activity of its downstream signaling pathways (58). Through this action, omega-3 indirectly inhibits multiple pro-cancer signaling pathways, including the EGFR-mediated RAS/MAPK cascade and the PI3K/Akt survival pathway (59). Indeed, in models such as breast cancer, EPA/DHA treatment reduces the phosphorylation levels of kinases such as Akt and ERK, and inhibits the PI3K/Akt signaling pathway, which reduces pro-survival signaling and promotes apoptosis (50, 60). At the same time, DHA also upregulates the activity of the stress kinase p38 MAPK and increases the level of ROS in tumor cells, triggering the apoptotic program of these cells (61). In summary, omega-3 fatty acids interfere with the normal operation of multiple signaling pathways, including EGFR and PI3K/Akt, by remodeling the lipid composition and microstructure of cell membranes, inhibiting proliferation and survival signals, and enhancing apoptotic signals in tumor cells. This dual effect on membrane structure and signaling is believed to be one of the important mechanisms of omega3's anticancer effects.

Omega-3 fatty acids target inflammation and cell survival pathways to exert anticancer effects. EPA and DHA can significantly downregulate the NF-κB pathway, thereby lowering the expression of COX-2, TNF-α, and other pro-inflammatory genes in tumors. This anti-inflammatory action creates a less favorable environment for tumor progression. Additionally, ω-3 PUFAs alter membrane lipid rafts and receptor signaling: DHA/EPA enrichment in cancer cell membranes disrupts EGFR localization, which in turn impairs downstream PI3K/Akt signaling. In breast cancer cells, such membrane remodeling by ω-3 has been shown to reduce Akt and ERK phosphorylation, diminishing pro-survival signals and promoting apoptosis. Beyond apoptosis, omega-3s may induce other forms of cell death; for example, DHA was reported to trigger pyroptosis in triple-negative breast cancer cells by activating caspase-1 and gasdermin D pores (62). Collectively, these findings confirm that ω-3 fatty acids can inhibit proliferative signaling and activate cell death programs while also mitigating cancer-related inflammation.

#### 3.3.3 Omega-3 fatty acids: exosomal miRNA transfer and tumor communication

**Figure 4:**
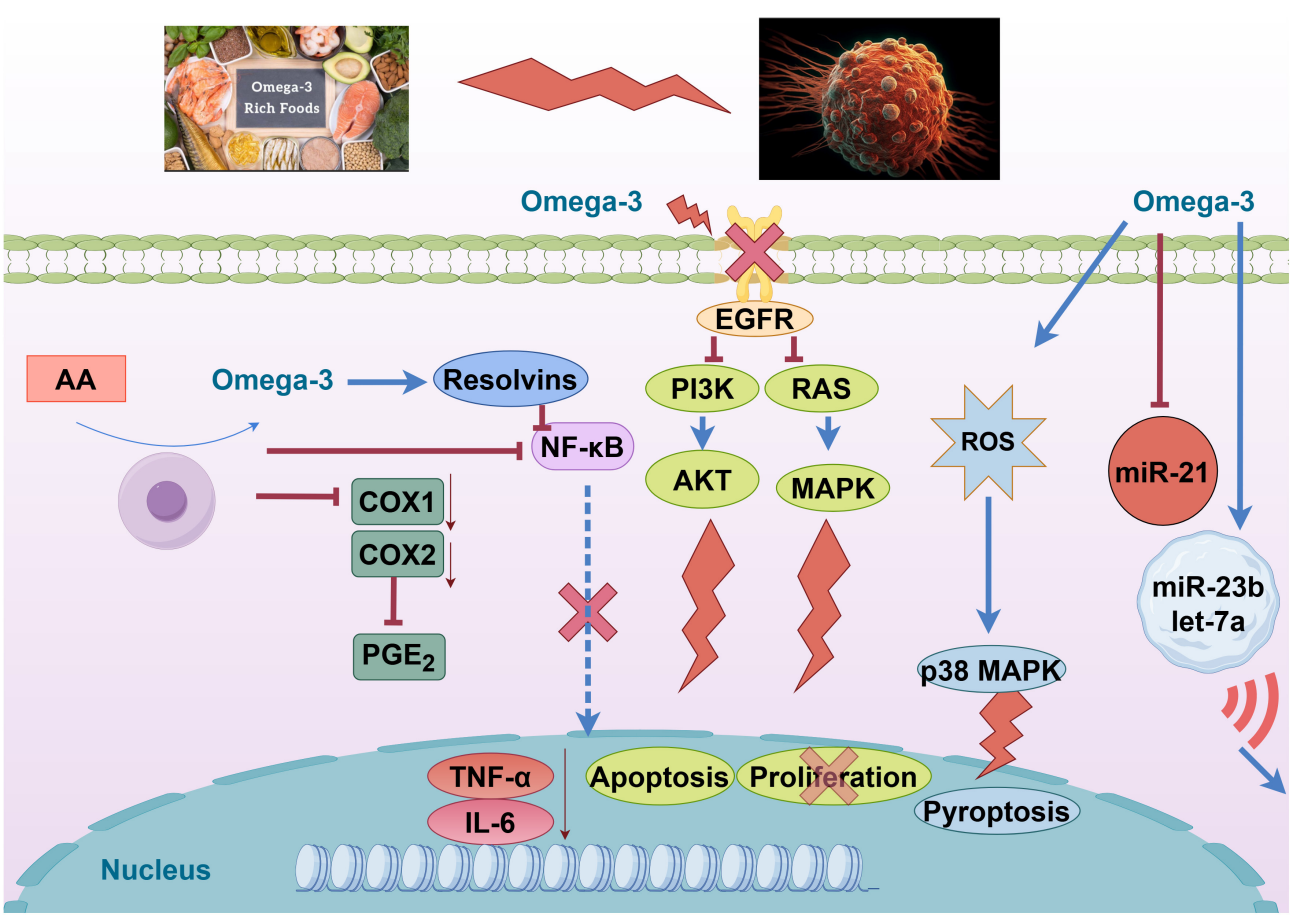
Omega-3 fatty acids modulate oncogenic and inflammatory pathways. Created with figdraw.com.

DHA and EPA demonstrate sophisticated miRNA-mediated mechanisms involving exosomal miRNA transfer between cancer and stromal cells. DHA treatment significantly reduces miR-21 expression in MCF-7 breast cancer cells while enhancing exosomal packaging of miR-23b, miR-27b, miR-320b, and let-7a (63). These exosomal miRNAs target PLAU, AMOTL1, NRP1, and ETS2 in endothelial cells, disrupting angiogenic signaling. Animal studies show that EPA/DHA combinations achieve a 60-70% reduction in tumor size compared to ALA alone, with eight-fold greater efficacy in HER2+ mouse models. The mechanism involves time-dependent miR-21 reduction at 1, 3, and 24 h post-treatment, though PTEN protein expression remains unchanged despite miR-21 suppression, suggesting complex regulatory networks (64). Clinical trials demonstrate safety and efficacy with EPA (1.6 g) + DHA (0.8 g) daily for 12 weeks in lung cancer patients, showing improved nutritional status and suppressed inflammatory markers (CRP, TNF-α, IL-6) (63). The preclinical evidence reveals that DHA suppresses non-small-cell lung cancer via the RvD1/miR-138-5p/FOXC1 pathway, providing mechanistic support for clinical applications (Figure 4).

#### 3.3.4 Potential to prevent cancer cachexia

Cancer cachexia is a syndrome characterized by weight loss, skeletal muscle loss, and metabolic disturbances, commonly seen in patients with advanced tumors. Omega-3 fatty acids have been recognized as one of the potential nutritional interventions for cachexia due to their anti-inflammatory and metabolic modulating effects (65). First, omega-3 reduces cachexia-related muscle wasting by inhibiting inflammation. Patients with cachexia often have high levels of inflammatory factors that activate protein degradation pathways and promote muscle breakdown (66, 67). Omega3's inhibition of inflammatory signals such as NF-κB reduces the activity of the ubiquitin–proteasome pathway in muscle—for example, by downregulating the expression of the muscle-specific ubiquitin ligase MuRF-1—thereby reducing skeletal muscle protein breakdown. At the same time, the reduction of inflammation also alleviates metabolic stress on the body, resulting in a decrease in the rate of muscle catabolism (68). Second, omega-3 helps improve the nutritional metabolic status of cancer patients. Studies have shown that omega-3 can activate muscle synthesis pathways, enhance amino acid uptake by myocytes, and increase the efficiency of muscle protein synthesis (69, 70). In addition, omega-3 improves mitochondrial function and reduces inflammation-induced oxidative stress, which overall contributes to the maintenance of lean body tissue (71, 72).

Clinical trials and meta-analyses provide support for the effect of omega-3 in alleviating cachexia. A study of patients with pancreatic cancer and gastrointestinal tumors reported that during omega-3 supplementation prior to chemotherapy, patients gained an average of approximately 2.5 kilograms of body weight, which was maintained after chemotherapy was initiated (73). Another randomized controlled trial of patients undergoing surgery for esophageal cancer showed that EPA-enriched enteral nutrition, given preoperatively and postoperatively, significantly protected against lean body mass (with significantly less postoperative lean body mass loss in the EPA group), resulting in an overall weight loss of only about 1.2 kilograms (compared to 1.9 kilograms in the control group) (74). Inflammatory mediators such as TNF-alpha and IL-8 levels were also significantly reduced in these patients receiving omega-3 nutritional support, suggesting a reduction in the systemic inflammatory response. Recent systematic evaluations and dose–response meta-analyses have further demonstrated that omega-3 supplementation has a small but beneficial effect on cancer cachexia; in particular, in elderly patients with cancer cachexia, omega-3 contributes to an increase in body weight (by about 1 kg) (75). It is important to note that the effect of omega-3 on cachexia varies among studies and may depend on dosage, stage of disease, and other factors (76). There is evidence that combining omega-3s with anti-inflammatory drugs or exercise may have more significant effects. A multimodal intervention study combining omega-3 supplementation (~2 grams per day) with moderate exercise in patients with malignant lung and pancreatic cancers resulted in an average weight gain of 4.5% and an improvement in muscle mass after 6 weeks, compared with continued weight loss in the control group (77). Overall, omega-3 fatty acids, due to their anti-inflammatory, anti-catabolic properties and improved nutritional status, show potential to alleviate cancer cachexia and may help reduce muscle wasting, stabilize body weight, and improve patients' quality of life. Further large-scale clinical studies will clarify the optimal strategy for using omega-3 in the comprehensive treatment of cancer cachexia.

### 3.4 Anticancer mechanisms of phytosterols and alkaloids

#### 3.4.1 Interference with cholesterol metabolism and tumor cell membrane structure

Phytosterols such as β-sitosterol are structurally similar to cholesterol and competitively interfere with cholesterol utilization by tumor cells (78). β-sitosterol inhibits cholesterol synthesis and uptake while promoting cholesterol efflux, thereby significantly reducing cholesterol levels in cancer cell membranes (79). The addition of β-sitosterol to colon cancer cells has been reported to reduce cell membrane cholesterol levels by about 26% (80). Depletion of cholesterol in cell membranes disrupts microdomain structures such as membrane lipid rafts, impairs the function of many raft-dependent receptors and signaling pathways, and inhibits tumor cell growth and survival (81). In addition, some alkaloids can affect cholesterol and lipid metabolism in tumor cells. Berberine has been shown to inhibit cancer cell proliferation by upregulating LDL receptors and inhibiting lipid synthesis pathways, such as fatty acid synthase, through activation of the AMPK pathway, which reduces cholesterol and lipid accumulation in tumor cells (82).

#### 3.4.2 Induction of apoptosis and inhibition of cancer cell proliferation

Both β-sitosterol and berberine inhibit cancer cell proliferation by inducing apoptosis through the mitochondria-mediated intrinsic pathway and blocking cell cycle progression (83). In apoptosis, β-sitosterol upregulates pro-apoptotic factors such as p53 and Bax, while downregulating anti-apoptotic proteins such as Bcl-2, leading to an increase in mitochondrial outer membrane permeability and the activation of key apoptotic enzyme cascades such as caspase-9 and −3, which induces programmed cell death (83). Furthermore, β-sitosterol increases the expression of death receptors and blocks the inhibitory effect of IAP proteins on caspases, making cancer cells more sensitive to exogenous death signals (84). Berberine also induces apoptosis via the mitochondrial pathway, promoting the release of cytochrome c from mitochondria, activating caspase-8, −9, and subsequent caspase-3 cascades, and triggering apoptosis by inhibiting the expression of Bcl-2 while increasing pro-apoptotic proteins (85). Regarding cell proliferation, these natural compounds can interfere with the activity of cell cycle regulatory proteins and induce cell cycle arrest. β-Sitosterol has been reported to inhibit a variety of cyclin-CDK complexes, upregulate the cell cycle inhibitor p27, and cause cells to arrest in the G1 phase, among others (86, 87). Berberine, on the other hand, triggers cell cycle arrest in the G0/G1 or G2/M phases by downregulating proteins such as Cyclin D1 and Cyclin E and upregulating p21, preventing cancer cells from entering the proliferation and division phases (88). By inducing apoptosis and halting the cell cycle, these two classes of compounds effectively inhibit the proliferation and growth of tumor cells.

#### 3.4.3 Influencing cancer-associated inflammation and immune escape

β-Sitosterol and berberine may also exert anticancer effects by modulating inflammatory mediators and immune responses in the tumor microenvironment (89–91). Chronic inflammation contributes to tumorigenesis and development, and these natural products inhibit pro-inflammatory signals, thereby weakening the pro-inflammatory microenvironment of tumors. β-Sitosterol selectively inhibits the activity of the pro-inflammatory mediator COX-2 and reduces the levels of the oncogenic PGE_2_ in tumor tissues, blocking the promotion of tumor cell proliferation by the COX-2/PGE_2_ inflammatory axis (92). Meanwhile, β-sitosterol enhances the antitumor immune response. Animal experiments showed that in a melanoma mouse model, tumors tend to limit the proliferation of the host's T lymphocytes, decrease their killing function, and inhibit the activity of macrophages. However, β-sitosterol administration significantly restored T-cell and macrophage function, increased NK cell activity, enhanced the body's immune surveillance of tumors, and significantly reduced the number of lung metastases (93). Berberine also has anti-inflammatory immunomodulatory effects: studies have shown that berberine antagonizes pro-inflammatory signaling pathways and inhibits immune cells from overproducing cytokines such as TNF-α, IL-6, and IL-1β (94). Furthermore, berberine blocks IFN-γ-induced IDO1 expression by inhibiting STAT1 phosphorylation (95). Inhibiting IDO1, one of the key enzymes involved in tumor immune escape, reduces the functional suppression of T cells by tumors and improves the effector function of immune cells in the tumor microenvironment. Moreover, berberine also reduces the expression of PD-L1 on the surface of tumor cells, interfering with the mechanism by which tumor cells use the PD-1/PD-L1 pathway to evade the immune system. By downregulating PD-L1, berberine enhances the recognition and killing of cancer cells by NK cells and T lymphocytes (96). In conclusion, phytosterols and alkaloids can disrupt cancer-associated inflammatory and immune escape mechanisms by inhibiting tumor pro-inflammatory responses and enhancing antitumor immune effects, demonstrating potential clinical applications in improving the tumor microenvironment.

Phytosterols like β-sitosterol primarily act by interfering with cholesterol-dependent cell functions and oncogenic signaling. The structural mimicry of cholesterol by β-sitosterol allows it to reduce membrane cholesterol, disrupting lipid raft-associated pathways. Mechanistically, β-sitosterol can induce cancer cell apoptosis by modulating key regulators: it elevates the Bax/Bcl-2 ratio and activates caspases via the intrinsic mitochondrial pathway. Recent studies also show that β-sitosterol suppresses pro-survival signaling cascades—for instance, an oxidized β-sitosterol derivative significantly downregulated phosphorylated ERK1/2 and NF-κB p65 levels, leading to increased ceramide accumulation and apoptosis in breast and liver cancer cells (97). Furthermore, β-sitosterol attenuates IκB degradation, thereby blocking NF-κB nuclear translocation and reducing the expression of inflammation-linked growth genes (80). On the other hand, alkaloids from plants often target cell division and transcriptional machinery. A classic example is paclitaxel, which binds to β-tubulin to stabilize microtubules—this causes G2/M cell cycle arrest and triggers apoptosis (98). Paclitaxel-induced mitotic stress leads to cleavage of procaspases and activation of the caspase cascade, effectively executing programmed cell death (99). Another potent plant alkaloid, triptolide, exerts its anticancer activity by downregulating oncogenic transcription pathways. Triptolide and its analogs have been shown to inhibit NF-κB signaling, which in pancreatic cancer cells results in elevated oxidative stress, reduced Bcl-2 expression, and activation of mitochondrial apoptosis pathways (100). In essence, phytosterols and alkaloids can interfere with tumor cell survival on multiple fronts—from membrane signaling and cell cycle regulation to intrinsic apoptosis—often targeting master regulators like NF-κB, Akt/mTOR, or microtubules.

#### 3.4.4 Alkaloids: comprehensive miRNA biogenesis modulation

Berberine targets multiple oncogenic miRNAs, including miR-21 (50–70% reduction in HCT116 cells), miR-23a (2.5-fold increase in hepatoma cells), and suppression of the miR-17–92 cluster in multiple myeloma. The compound regulates miRNA expression through AMPK signaling pathway activation and chromatin remodeling, with PDCD4 protein levels increasing by 60% following miR-21 inhibition (101). Capsaicin demonstrates TRPV1-mediated miRNA modulation, with miR-449a significantly upregulated (3–4-fold) in prostate cancer cells, promoting androgen receptor degradation (40–50% reduction) (102). The compound enhances miR-34a expression two-fold in non-small-cell lung cancer, promoting Bax-dependent apoptosis through calcium influx and ROS generation (103). Caffeine affects colon carcinogenesis through miR-21a-5p suppression (fold change >2) and activates miR-30c/miR-96, targeting KRAS expression (30–40% reduction) (104). Combination treatments with chlorogenic acid show synergistic miRNA modulation affecting miR-144-3p (2.2-fold upregulation) in hepatocarcinogenesis prevention studies (105).

### 3.5 Anticancer mechanisms of isothiocyanates

#### 3.5.1 Induction of apoptosis and cell cycle arrest

Isothiocyanates (ITCs) are bioactive compounds derived from glucosinolate precursors abundant in cruciferous vegetables (e.g., broccoli and watercress) (106). Notable examples include sulforaphane (SFN) from broccoli and phenethyl isothiocyanate (PEITC) from watercress (107). These phytochemicals have been widely linked to cancer prevention and therapy due to their broad spectrum of anticancer mechanisms. ITCs can trigger programmed cell death in cancer cells and halt their proliferation. Numerous *in vitro* studies show that ITCs induce apoptosis via both intrinsic and extrinsic pathways (108)—for instance, through caspase activation (109), mitochondrial dysfunction (110), and oxidative stress generation (111). A recent chemical proteomics study identified the pro-apoptotic Bcl-2 family protein BID as a direct covalent target of PEITC; its activation leads to mitochondrial outer membrane permeabilization and robust apoptosis in tumor cells (112). Consistently, compounds like SFN are known to cause G2/M cell cycle arrest in various cancer cell lines, thereby suppressing tumor growth (113). The promotion of apoptotic cell death and cell cycle inhibition is a central antitumor mechanism of ITCs in many cancer types (114).

#### 3.5.2 Activation of the Keap1–Nrf2 pathway and detoxification

ITCs are potent activators of the Keap1–Nrf2 signaling pathway, which upregulates cytoprotective genes. By modifying Keap1 cysteine residues, ITCs stabilize Nrf2, leading to enhanced transcription of antioxidant response elements and phase II detoxification enzymes (113). Through this mechanism, ITCs bolster cellular defenses against oxidative damage and help eliminate carcinogens. For example, SFN is one of the most potent naturally occurring inducers of phase II enzymes, contributing to chemoprevention (115). This antioxidative detoxification mechanism protects normal cells from genotoxic stress; however, it can also create a reductive microenvironment that paradoxically may aid advanced tumors, reflecting the complex dual role of Nrf2 in cancer. Overall, the Nrf2-mediated induction of protective enzymes is a major pathway through which ITCs exert cancer-preventive effects (116).

#### 3.5.3 Epigenetic modulation

A distinctive molecular action of ITCs, especially SFN, is the inhibition of histone deacetylases (HDACs). SFN functions as an HDAC inhibitor in mammalian cells, leading to increased histone acetylation and re-expression of epigenetically silenced tumor suppressor genes (117). This epigenetic reprogramming is partly linked with other ITC effects: HDAC inhibition by SFN has been associated with the suppression of phase I carcinogen-activating enzymes and the concurrent induction of phase II detoxifiers (118). In cancer models, SFN's HDAC-inhibitory activity has been shown to reactivate pathways that induce cell cycle arrest and apoptosis (119). Likewise, PEITC and other ITCs can influence DNA methylation and non-coding RNA expression, further contributing to the epigenetic regulation of cancer cell fate (120). The ability to remodel the epigenetic landscape underscores the multitargeted anticancer potential of ITCs.

#### 3.5.4 Modulation of the tumor microenvironment

ITCs also exert anticancer effects by altering the tumor microenvironment. They have demonstrated anti-inflammatory activity; notably, SFN suppresses the NF-κB pathway, reducing pro-inflammatory cytokine production that otherwise supports tumor growth and immune evasion (121). ITCs can inhibit angiogenesis and metastasis; early studies showed that ITCs block new blood vessel formation and epithelial–mesenchymal transition (EMT) in tumors (122). For example, SFN and PEITC have been reported to downregulate matrix metalloproteinases and other mediators of invasion, thereby impairing cancer cell migration and metastatic spread (123). Additionally, ITCs may enhance antitumor immunity: in experimental models, they promote the activity and proliferation of immune effector cells such as natural killer and T cells, contributing to immunosurveillance against tumors (113). By modulating inflammatory pathways, blood supply, and immune cell responses in the tumor microenvironment, ITCs create conditions that are less favorable for cancer progression.

#### 3.5.5 Isothiocyanates emerge as potent miRNA modulators across cancer types

**Figure 5:**
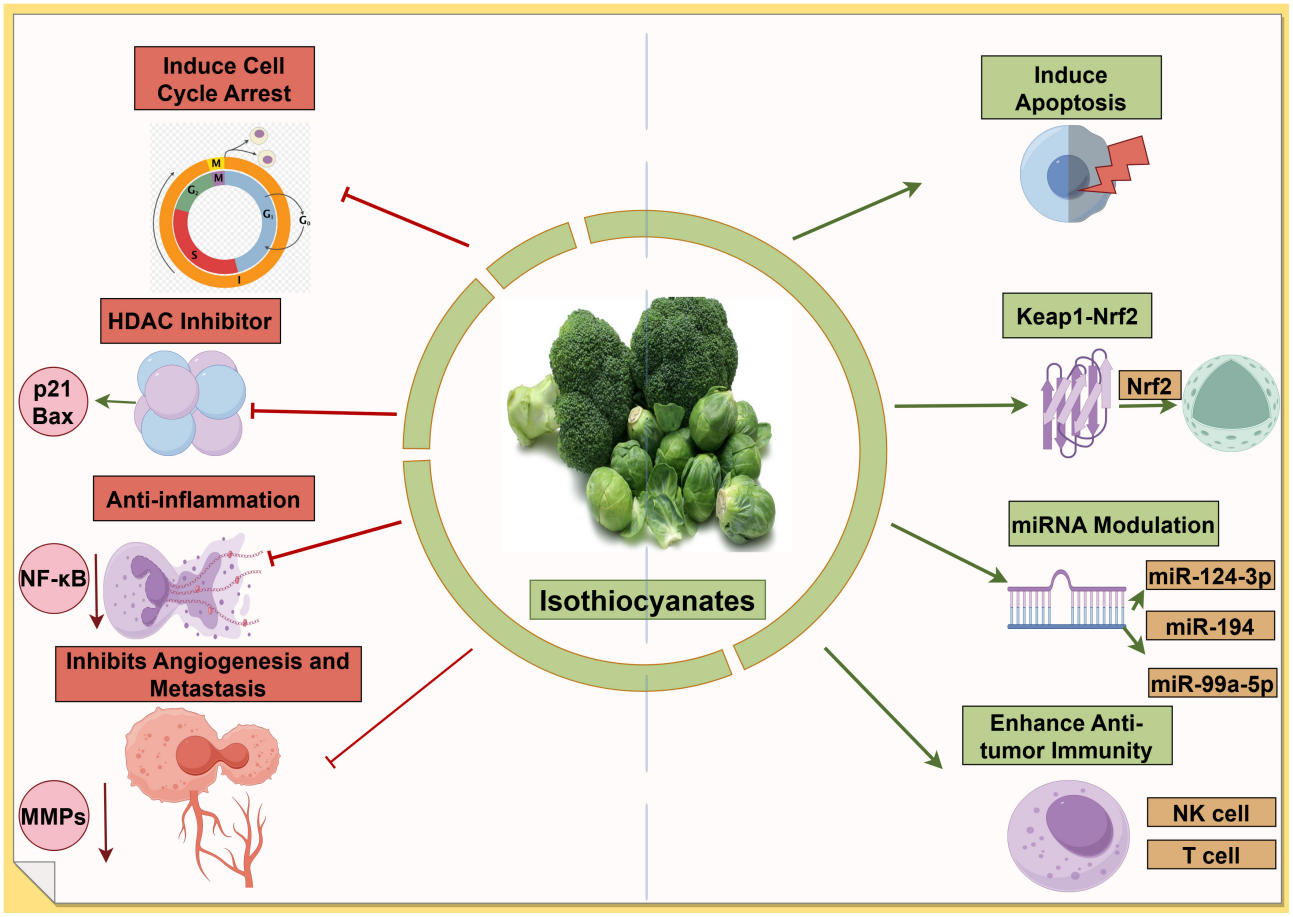
Isothiocyanates target multiple cancer pathways. The green arrows refer to promotion or increase, whereas the red hammerhead lines refer to inhibition. Created with figdraw.com.

Sulforaphane demonstrates extensive miRNA effects with well-characterized targets across multiple cancer types. Zhang et al. (124) identified the upregulation of miR-3919 in prostate cancer cells (PC-3, DU145), where the miR-3919/DJ-1 axis mediates antitumor effects. In nasopharyngeal cancer, sulforaphane upregulates miR-124-3p, which directly targets STAT3 signaling and reduces cancer stem cell markers, including β-catenin, Nanog, and Oct3/4 in HONE1, SUN1, CNE1, and CNE2 cell lines (125). Phenethyl isothiocyanate (PEITC) specifically targets the miR-194/BMP1 axis in prostate cancer. Oligonucleotide microarray analysis followed by qPCR validation revealed the upregulation of miR-194 in LNCaP and PC3 cells, with direct targeting of bone morphogenetic protein 1 (BMP1) in the 3'-UTR region. This mechanism decreases MMP2 and MMP9 expression, effectively suppressing cell invasion through Matrigel (126). Benzyl isothiocyanate (BITC) demonstrates unique miRNA restoration in bladder cancer. Recent comprehensive studies using qPCR microarray identified 79 aberrantly expressed miRNAs, with miR-99a-5p showing dose-dependent upregulation in 5637, RT4, HT1376, HT1197, and T24 cell lines. Validation studies confirmed the direct targeting of IGF1R, FGFR3, and mTOR, with antisense miR-99a-5p sequences reversing BITC effects (127) (Figure 5).

#### 3.5.6 Potential clinical value

Given their multitargeted mechanisms and low toxicity profiles, ITCs are actively explored in clinical settings as chemopreventive or adjunct therapeutic agents (128). Epidemiological data link high cruciferous vegetable intake with reduced cancer incidence in humans (113). More directly, early-phase clinical trials with purified ITCs or ITC-rich preparations have yielded encouraging results. In a recent randomized Phase II trial, a broccoli sprout-derived SFN supplement given to former smokers for 12 months significantly decreased the Ki-67 proliferative index in their bronchial epithelium compared to an increase in the placebo group. No serious adverse events were reported, underscoring the tolerability of dietary ITC. Although that trial did not reverse pre-neoplastic changes, the reduction in cell proliferation supports further investigation of SFN for lung cancer chemoprevention (129). Additionally, combination therapy studies indicate that ITCs can sensitize tumors to conventional chemotherapy and overcome drug resistance, thereby improving therapeutic outcomes when used as adjuncts (130). It is important to note that ITCs exhibit a hormetic dose-response: low dietary doses may have negligible or even protective effects on cells, whereas higher pharmacological doses are required for cytotoxic antitumor activity (113). This biphasic behavior necessitates careful optimization of dosing in any future clinical applications. Nevertheless, the consensus of recent literature is that isothiocyanates are promising cancer-fighting phytochemicals with potential roles in both cancer prevention and therapy. Their ability to target multiple pathways—from initiating apoptosis and detoxifying carcinogens to reprogramming epigenetics and the tumor microenvironment—positions ITCs as attractive candidates for integrative oncology approaches (113).

### 3.6 Anticancer mechanisms of polysaccharides

#### 3.6.1 Dual roles in apoptosis induction and immune potentiation

Polysaccharides are high-molecular-weight carbohydrate compounds abundant in functional foods such as mushrooms, algae, and medicinal herbs. Representative anticancer polysaccharides include β-glucans (from yeast and fungi) and fucoidan (from brown seaweed), among others. These bioactive polysaccharides exhibit multifaceted anticancer mechanisms. Induction of apoptosis is a major pathway—many polysaccharides trigger caspase-dependent programmed cell death in tumor cells (131). For example, β-glucans from fungi can activate initiator caspase-8 and effector caspase-3, leading to mitochondrial cytochrome C release and apoptosis in cancer cells (132). Polysaccharides also cause cell cycle arrest, thereby halting the proliferation of malignant cells (133). In parallel, they exert significant immunomodulatory effects: polysaccharides stimulate the immune system's antitumor response by activating macrophages, T lymphocytes, natural killer cells, and dendritic cells, and by promoting the release of cytokines such as TNF-α, IFN-γ, and IL-2 (134). This immune regulation helps the body recognize and destroy cancer cells more effectively. Indeed, enhancing host immunity is considered a primary antitumor pathway for plant polysaccharides (135).

#### 3.6.2 Oxidative stress modulation and signaling pathway interference

Another important mechanism is oxidative stress regulation. Polysaccharides often possess antioxidant properties that allow them to modulate ROS levels and related signaling. By scavenging excessive ROS, polysaccharides can prevent the activation of pro-tumorigenic pathways (e.g., NF-κB and MAPK) that are driven by oxidative stress (136). Some polysaccharides can also induce mild ROS generation in cancer cells, pushing them beyond their oxidative threshold and triggering cell death, all while protecting normal cells—an effect linked to the activation of endogenous antioxidant pathways like Nrf2 (137). Furthermore, polysaccharides interfere with key signaling pathways that regulate survival, metastasis, and angiogenesis in tumors. For instance, a dandelion-derived polysaccharide was shown to inhibit the PI3K/Akt pathway, downregulating HIF-1α and VEGF expression, and thereby suppressing tumor angiogenesis (138). Many polysaccharides also inhibit NF-κB signaling, which in turn reduces pro-inflammatory mediators in the tumor microenvironment and sensitizes cancer cells to immune attack (139). In addition, the marine polysaccharide fucoidan exemplifies broad pathway modulation: it can induce cancer cell apoptosis and cell cycle arrest, block metastasis and angiogenesis, and modulate various signaling molecules involved in tumor growth (133).

#### 3.6.3 Epigenetic remodeling, therapeutic synergy, and nanodelivery potential

Notably, emerging research indicates that polysaccharides can act as epigenetic modulators in cancer. Certain plant-derived polysaccharides (e.g., from *Astragalus* or *Ganoderma lucidum*) promote DNA demethylation of tumor suppressor genes via TET enzymes, inhibit oncogenic histone-modifying enzymes, and regulate cancer-related microRNAs (140). Through these epigenetic reprogramming actions, polysaccharides may reactivate silenced tumor suppressor pathways and impede tumor progression. This epigenetic influence adds a new dimension to their anticancer activity. Moreover, polysaccharides often work synergistically with conventional therapies. Clinically, mushroom β-glucans have been used as adjuvants to chemotherapy and radiotherapy to boost immune function; co-administration of fungal β-glucan has been reported to mitigate therapy-induced leukopenia and immune suppression, improving patient recovery (141). Polysaccharides from Angelica sinensis similarly protected hematopoietic cells from cyclophosphamide toxicity (142). Preclinical studies confirm that combining polysaccharides with chemotherapeutics can enhance tumor killing while reducing side effects through immune modulation and tissue protection (140). For example, *Astragalus* polysaccharide (APS) was shown to inhibit the formation of the pre-metastatic niche in lung cancer by blocking the S1PR1/STAT3 signaling pathway, thereby preventing metastasis when used alongside standard treatment (143). In addition, polysaccharides hold promise as drug delivery vehicles in cancer therapy due to their biocompatibility and tunable structure. Natural polymers like chitosan, pectin, and alginate are being developed as carriers in nanoparticle and hydrogel systems to deliver anticancer drugs more specifically to tumors. Such polysaccharide-based delivery systems can improve drug stability and targeting, enhancing therapeutic efficacy. Overall, polysaccharides from functional foods exert anticancer effects through a combination of apoptotic induction, immune activation, oxidative stress attenuation, and pathway interference, with additional roles in epigenetic regulation and supportive therapy. These diverse mechanisms underscore their potential as nontoxic adjuvants and therapeutic agents in future cancer treatment strategies (140).

#### 3.6.4 Polysaccharides regulate tumor-suppressive and oncogenic miRNA networks

Fucoidan exhibits dual miRNA regulation affecting both tumor-suppressive and oncogenic pathways. Wu et al. and Yan et al. demonstrated the upregulation of miR-29c and the downregulation of miR-17-5p in breast cancer (MCF-7, MDA-MB-231) and hepatocellular carcinoma (SMMC-7721) cells. The upregulation of miR-29c targets ADAM12 and inhibits EMT, while the downregulation of miR-17-5p restores PTEN pathway function (144). *Astragalus* polysaccharides (APS) show cancer-specific miRNA targeting. Tao et al. identified the upregulation of miR-195-5p in non-small-cell lung cancer, which leads to suppressed proliferation and invasion (145). In prostate cancer, a study demonstrated that APS-mediated upregulation of miR-133a in DU145 cells effectively inhibited proliferation, invasion, and migration by targeting oncogenic pathways (146). Chitosan-based delivery systems enable precise miRNA modulation. Chitosan nanoplexes have been developed for the targeted delivery of miR-200c and miR-141 to breast cancer cells (MCF-7, MDA-MB-231, MDA-MB-435), successfully restoring miRNA expression levels to those observed in non-cancerous cells. Advanced formulations utilizing GE11 peptide-conjugated chitosan nanoparticles can specifically target EGFR-overexpressing triple-negative breast cancer cells, leading to the downregulation of miR-21 and inhibition of the AKT/ERK signaling axis (147).

### 3.7 Anticancer mechanisms of phenolic acids

#### 3.7.1 Apoptosis induction and suppression of angiogenesis and metastasis

Phenolic acids are a major class of plant-derived polyphenols (non-flavonoid type) widely present in fruits, vegetables, teas, coffee, and other foods. Key representatives include gallic acid (GA) (a hydroxybenzoic acid found in berries, tea, and other sources) and caffeic acid (a hydroxycinnamic acid abundant in coffee, apples, and herbs), along with others like ferulic and p-coumaric acids (148). These compounds are well known for their potent antioxidant properties and have demonstrated broad-spectrum anticancer activities. Phenolic acids combat cancer through multiple molecular mechanisms. They induce apoptosis in tumor cells via both intrinsic and extrinsic pathways, often associated with the activation of pro-apoptotic proteins and caspase cascades. For example, GA can trigger mitochondrial apoptotic signals: it upregulates Bax, downregulates Bcl-2, and activates caspase-9 and −3, leading to programmed cell death in various carcinoma cells (148). They also cause cell cycle arrest; GA has been shown to halt cancer cell cycles at the S or G2/M phase, thereby suppressing proliferation (149). In addition, phenolic acids exhibit anti-angiogenic and anti-metastatic effects. *In vivo* studies indicate these compounds can inhibit tumor neovascularization and invasion; for instance, phenolics were observed to reduce VEGF levels and MMP activity, thereby impairing angiogenesis and metastatic spread. Caffeic acid and its derivatives can block pro-angiogenic signaling: caffeic acid phenethyl ester (CAPE) has been reported to suppress STAT3-mediated VEGF expression, inhibiting tumor angiogenesis in renal carcinoma models. Likewise, caffeic acid itself directly inhibited ERK1/2 (MAPK) activity in melanoma and epidermoid carcinoma cells, disrupting a key pathway that promotes tumor growth and migration (150).

#### 3.7.2 Dual modulation of oncogenic and antioxidant signaling pathways

Phenolic acids strongly modulate cellular signaling pathways that govern survival and inflammation in cancer. A prominent example is their inhibition of the PI3K/Akt pathway—GA treatment in lung cancer cells led to downregulation of PI3K/Akt alongside increased p53 levels, which in turn activated downstream executioner caspases and cell cycle inhibitors (p21Cip1/p27Kip1), culminating in apoptosis and growth arrest (151). Many phenolic acids are also known to suppress the NF-κB pathway, thereby reducing pro-inflammatory cytokines in the tumor microenvironment and sensitizing cancer cells to immune attack (150). CAPE, for instance, is a well-documented NF-κB inhibitor and has demonstrated antitumor efficacy by blocking NF-κB–driven survival signals in prostate and ovarian cancer cells (152). In addition to inhibiting oncogenic signals, these compounds often activate stress–response pathways in cells. As antioxidants, phenolic acids typically activate the Nrf2 pathway in normal cells, enhancing cytoprotective antioxidant enzymes. This effect can protect healthy tissues from oxidative damage and contributes to chemoprevention (153). Interestingly, recent research has highlighted a dual role for Nrf2 in cancer: while basal Nrf2 activity aids normal cell defense, cancer cells can exploit Nrf2 for chemoresistance. Certain phenolic compounds can inhibit Nrf2 in tumor cells, stripping them of antioxidant defenses (154). This targeted Nrf2 inhibition drives cancer cells into lethal oxidative stress and can improve the efficacy of chemo-radiotherapy. Thus, phenolic acids can both quench excess radicals to prevent cancer initiation and, in established tumors, undermine the antioxidant shield of cancer cells.

#### 3.7.3 Epigenetic reprogramming and oncogene suppression

Phenolic acids also exert epigenetic effects that restore normal regulatory controls in cancer cells. They can influence DNA methylation and histone modifications that are dysregulated in tumors. For example, GA has been shown to bind and stabilize G-quadruplex DNA structures in oncogene promoters (such as c-Myc), leading to transcriptional downregulation of those genes (155). Such an interaction essentially mimics telomerase/telomere inhibition and triggers nucleolar stress, contributing to tumor growth suppression. Indeed, GA was found to selectively kill colorectal cancer cells in part by inducing G-quadruplex-mediated telomeric dysfunction (156). More broadly, dietary polyphenols like gallic and ferulic acid are recognized for reversing aberrant epigenetic marks—they can inhibit DNA methyltransferases or HDACs, reactivating silenced tumor suppressor genes (153). Through these mechanisms, phenolic acids help reprogram cancer cells to a less aggressive state.

#### 3.7.4 Phenolic acids demonstrate pathway-specific miRNA regulation

Ferulic acid specifically targets the miR-221/TP53INP1 autophagy axis. Sweed et al. demonstrated significant downregulation of miR-221 in colorectal cancer (Caco-2 cells), leading to upregulation of TP53INP1 (*p* < 0.0001) and enhanced autophagy-mediated cell death with S-phase cell cycle arrest (157). Gallic acid affects complex miRNA regulatory networks. Recent RNA-sequencing analysis revealed 13 upstream miRNAs in gallic acid's therapeutic network in cervical cancer, with regulatory networks involving lncRNA/circRNA–miRNA–mRNA pathways targeting genes such as CDC20, DLGAP5, and KIF20A (158). In colorectal cancer, low-dose gallic acid ( ≤ 100 μM) reduces invasiveness through specific miRNA regulation without inducing cell death (159). Chlorogenic acid induces cancer differentiation through coordinated miRNA regulation. Key miRNAs include miR-20a, miR-93, and miR-106b, which regulate p21 targeting through the seed sequence AAGUGC. Dual luciferase reporter assays confirmed miRNA-p21 interactions, with upregulation of the NFATC2 and NFATC3 genes in immune pathways (160).

#### 3.7.5 Synergistic therapeutics and nanodelivery innovations

Notably, phenolic acids can enhance conventional cancer therapies and have shown synergistic effects with chemo-radiotherapy. GA, for instance, significantly boosted the efficacy of standard chemotherapeutics (e.g., cisplatin, doxorubicin, and 5-FU) when combined with gamma irradiation in oral cancer cells, by promoting superoxide-dependent apoptosis and blocking protective autophagy in an Nrf2-dependent manner (161). This combination led to increased cancer cell kill rates, illustrating GA's potential as a radiosensitizer. Similarly, caffeic acid has been reported to synergize with paclitaxel: co-treatment of caffeic acid with low-dose paclitaxel inhibited non-small-cell lung cancer growth more effectively than paclitaxel alone, with a marked increase in apoptosis and suppression of proliferative signals in tumor cells (162). Phenolic acids may also mitigate chemotherapy side effects through their antioxidant and anti-inflammatory properties, thereby improving therapeutic indices. In terms of drug delivery, these small-molecule phytochemicals can be chemically modified or encapsulated to enhance their bioavailability. For example, caffeic acid's derivative CAPE has been formulated in nanocarriers (such as γ-cyclodextrin inclusion complexes) to improve its stability and anticancer potency (148). Such strategies aim to harness phenolic acids in more effective forms for clinical use.

### 3.8 Anticancer mechanisms of flavonols

#### 3.8.1 Antioxidant and pro-apoptotic agents in cancer therapy

Flavonols are a prominent subclass of dietary flavonoids found abundantly in fruits, vegetables, tea, and wine. Representative flavonols include quercetin, kaempferol, myricetin, fisetin, isorhamnetin, and morin, which are present in foods such as onions, berries, apples, brassica vegetables, and capers (163). These compounds exhibit broad-spectrum anticancer activities through multiple mechanisms. Antioxidant and free radical scavenging are key properties—flavonols directly neutralize ROS and upregulate cellular antioxidant defenses, protecting DNA from oxidative damage and mutagenesis (163). Paradoxically, in the context of tumors, flavonols can exert pro-oxidant effects at higher concentrations, elevating ROS within cancer cells to trigger oxidative stress-induced apoptosis. Flavonols also potently induce apoptosis in cancer cells via the intrinsic (mitochondrial) pathway. Quercetin and related flavonols modulate Bcl-2 family proteins, increasing pro-apoptotic Bax and reducing anti-apoptotic Bcl-2, which promotes cytochrome c release and caspase-3/9 activation (164). They can also activate extrinsic apoptosis by upregulating death receptors or downstream effectors. In parallel, flavonols cause cell cycle arrest in various phases to halt cancer cell proliferation. For example, quercetin and fisetin have been shown to induce G0/G1 or G2/M phase arrest by downregulating cyclins (Cyclin B, D, E) and cyclin-dependent kinases (CDK1, CDK2, CDK4/6) while upregulating CDK inhibitors like p21Cip1 and p27Kip1 (165). This disruption of cell cycle progression limits the unchecked growth of cancer cells.

#### 3.8.2 Targeting cancer progression through signaling, immunity, and epigenetic regulation

Molecular signaling pathways central to cancer cell survival are major targets of flavonols. Quercetin is exemplary in interfering with pro-oncogenic signaling: it inhibits the PI3K/Akt/mTOR and MAPK/ERK pathways, thereby suppressing downstream survival signals and proliferative drivers. By inhibiting Akt and ERK phosphorylation, quercetin triggers apoptosis and growth arrest selectively in tumor cells. Flavonols also block the continuous activation of transcription factors like NF-κB and STAT3 that promote tumor progression. Quercetin has been shown to downregulate NF-κB activity, leading to reduced expression of inflammatory cytokines and survival genes, and to modulate JAK/STAT signaling—enhancing immune recognition of cancer cells while diminishing immunosuppressive signals (164). Notably, a recent study in hepatocellular carcinoma cells found that quercetin induced robust apoptosis by inhibiting the PI3K/Akt/mTOR axis (via downregulation of P4HA2, a proline hydroxylase) and activating p53-dependent death pathways. Many flavonols (e.g., kaempferol and isorhamnetin) similarly target multiple nodes in signaling networks to shift the balance toward cancer cell death and away from survival (163). In addition, flavonols can inhibit tumor angiogenesis by downregulating VEGF, HIF-1α, and other angiogenic factors, starving tumors of blood supply. Quercetin and kaempferol have each been reported to reduce VEGF secretion and disrupt new blood vessel formation in tumor models (166). They also suppress metastasis through anti-invasive and anti-migratory effects—for instance, by inhibiting matrix metalloproteinases (MMP-2, MMP-9) and reversing EMT. Through attenuation of NF-κB and AP-1 signaling, flavonols reduce MMP production, thereby impeding the degradation of the extracellular matrix required for metastasis (167).

Some flavonols (e.g., fisetin) are reported to upregulate E-cadherin and tight junction proteins, counteracting EMT and metastatic spread. Moreover, flavonols exert immunomodulatory effects that enhance antitumor immunity. By curbing chronic inflammation (via NF-κB inhibition) and modulating the tumor microenvironment, they create conditions more favorable for immune cell attack (164). Quercetin's suppression of STAT3, for example, can boost cytotoxic T-cell activity and reduce immune evasion, since STAT3 in tumors often drives the production of immunosuppressive factors. Flavonols have also been noted to influence macrophage polarization and enhance the efficacy of immune checkpoint therapies in preclinical studies (163). Finally, flavonols may induce epigenetic modifications in cancer cells. Some act as epigenetic modulators by inhibiting HDACs or DNA methyltransferases or by altering microRNA expression, thereby reactivating silenced tumor suppressor genes. For instance, quercetin has been identified as a cancer epigenetic regulator that can restore p53 activity by disrupting oncogenic protein interactions (e.g., blocking the YY1–p53 complex) and increasing pro-apoptotic gene expression (168). Through this multifaceted array of actions—antioxidant, pro-apoptotic, anti-proliferative, anti-angiogenic, anti-metastatic, immunomodulatory, and epigenetic—flavonols demonstrate significant potential in cancer prevention and therapy. Notably, their relatively low toxicity and presence in everyday foods make them attractive as chemopreventive agents. Ongoing research, including nanocarrier-based delivery of quercetin, aims to overcome bioavailability issues and translate flavonols into effective adjuvant cancer treatments. The accumulating evidence supports flavonols as key functional food ingredients that can target multiple hallmarks of cancer simultaneously, contributing to reduced tumor growth and enhanced responses to conventional therapies (163).

#### 3.8.3 Flavonols modulate competing endogenous RNA networks

Quercetin demonstrates broad-spectrum miRNA effects across multiple cancer types. In cervical cancer (HeLa cells), quercetin upregulates tumor suppressor miRNAs miR-26b, miR-126, and miR-320a, with miR-320a directly targeting β-catenin (CTNNB1) to inhibit the Wnt pathway. Clinical validation studies in 264 lung cancer patients showed a correlation between dietary quercetin and higher expression of the let-7 family and miR-146a (169). Kaempferol and fisetin exhibit synergistic miRNA modulation. Kubina et al. identified IC50 values of 38.85 μM (SCC-9) and 62.34 μM (SCC-25) in head and neck cancer cells, demonstrating dose-dependent apoptosis induction and G1 phase cell cycle arrest (170). The mechanism involves modulation of the ERK signaling pathway and inhibition of the NF-κB pathway. Myricetin shows consistent anti-neoplastic activity with 9.62-9.74% absolute bioavailability, demonstrating colorectal cancer prevention through the inhibition of tumorigenesis and reduction of polyp size. The mechanism involves activation of the apoptosis pathway, anti-angiogenic effects, and inhibition of matrix metalloproteinases (171).

### 3.9 Anticancer mechanisms of amides

#### 3.9.1 Amide-bearing phytochemicals as mitochondrial apoptosis inducers in cancer

Amide-bearing compounds from edible plants—particularly capsaicinoids and related alkylamides—have emerged as potent anticancer agents. The most prominent example is capsaicin (trans-8-methyl-N-vanillyl-6-nonenamide), the spicy component of chili peppers (*Capsicum* species). Another is piperine, the pungent alkaloid in black pepper (*Piper nigrum*), along with analogs like piperlongumine from long pepper. These dietary amides exhibit multifaceted antitumor activities. Capsaicin, in particular, has been widely studied and influences numerous cancer hallmarks. One major mechanism is the induction of apoptosis. Capsaicin can trigger programmed cell death via both extrinsic and intrinsic pathways. It binds to and activates the TRPV1 vanilloid receptor—a calcium-permeable ion channel—on cancer cells, leading to a surge in intracellular Ca^2+^. This Ca^2+^ overload precipitates mitochondrial dysfunction: capsaicin causes a loss of mitochondrial membrane potential, opening of the permeability transition pore, and release of cytochrome c, thereby activating the caspase cascade and resulting in apoptotic cell death (172).

In human anaplastic thyroid carcinoma and glioma cells, capsaicin-induced Ca2+ influx through TRPV1 has been shown to initiate p38 MAPK signaling and mitochondrial apoptosis, effects abrogated by TRPV1 antagonists (173). Capsaicin can also induce apoptosis independently of TRPV1 through direct modulation of stress and survival kinases. It has been found to activate the pro-apoptotic kinase AMPK and the tumor suppressor p53, as well as c-Jun N-terminal kinase (JNK), leading to apoptosis even when TRPV1 is blocked (174). Additionally, capsaicin directly interacts with the electron transport chain in mitochondria, enhancing ROS generation and promoting a change in the Bax/Bcl-2 ratio in favor of apoptosis (175). For example, in pancreatic cancer models, capsaicin raised intracellular ROS and upregulated Bax while downregulating Bcl-2, culminating in caspase-dependent apoptosis *in vitro* and *in vivo* (172). Piperine and related pepper alkaloids likewise induce apoptosis by increasing mitochondrial oxidative stress and activating executioner caspases (103). These amides often cause cancer cells to undergo morphological apoptosis (cell shrinkage, chromatin condensation) without harming normal cells at equivalent doses (176).

#### 3.9.2 Amide compounds induce cell cycle arrest to suppress cancer cell proliferation

Cell cycle arrest is another anticancer mechanism of amide compounds. Capsaicin has demonstrated the ability to halt cell cycle progression in multiple cancer types, preventing proliferation. In breast and oral carcinoma cells, capsaicin treatment has been shown to cause a G1-phase arrest by downregulating cyclin D/CDK4/6 and upregulating the CDK inhibitor p21, whereas in other models (e.g., KB nasopharyngeal carcinoma, MCF-7 breast cancer), it induced a G2/M arrest associated with reduced Cyclin B and CDK1 levels (172). The molecular targets underlying capsaicin's cell cycle effects include the E2F–cyclin pathway and sirtuin signaling. Capsaicin has been reported to inhibit oncogenic tNOX (ENOX2), leading to decreased NAD+ and SIRT1 activity, which in turn stabilized p53 and c-Myc—enforcing the G1/S checkpoint and blocking cell cycle progression (165). In bladder cancer cells, capsaicin similarly downregulated SIRT1 and upregulated p53, contributing to a sustained arrest and subsequent apoptosis (177). Through such modulation of cell cycle regulators and checkpoint control, capsaicin and piperine limit the proliferative capacity of cancer cells.

#### 3.9.3 Dietary amides as antioxidant, anti-inflammatory, and immune-modulating agents in cancer prevention

Dietary amides also exhibit significant antioxidant and anti-inflammatory actions that contribute to cancer prevention. At lower doses, capsaicin functions as an antioxidant in normal tissues; it can directly neutralize free radicals (e.g., ABTS and DPPH radicals in chemical assays) and elevate cellular antioxidant enzymes. Capsaicin has been shown to induce the Nrf2/ARE pathway, leading to increased expression of heme oxygenase-1, glutathione-S-transferase, superoxide dismutase, and catalase (178, 179). This Nrf2-mediated antioxidant response helps protect cells from DNA damage caused by carcinogens and ROS. In fact, capsaicin's chemopreventive effects *invivo* have been linked to its ability to inhibit carcinogen activation (e.g., by suppressing CYP450 enzymes that produce reactive metabolites) and to mitigate oxidative stress and inflammation in tissues (180). On the other hand, within established tumors, capsaicin often acts as a pro-oxidant and anti-inflammatory agent to damage cancer cells and their microenvironment. It inhibits chronic inflammatory signaling by targeting NF-κB, a master regulator of inflammation and cell survival.

Capsaicin can block the nuclear translocation of NF-κB and downregulate NF-κB–controlled genes (COX-2, TNF-α, IL-6), thereby reducing the pro-inflammatory environment that fosters tumor growth (167). For example, in *H. pylori*-associated gastric tumorigenesis, capsaicin's known inhibition of NF-κB led to lower iNOS and cytokine levels, alleviating inflammation and mucosal damage (181). By quelling inflammation, capsaicin not only directly impairs tumor cell proliferation but also may restore immune surveillance. Notably, capsaicin has been identified as a selective inhibitor of STAT3 signaling in tumors (182). It promotes the lysosomal degradation of STAT3 protein, as recently demonstrated *in vitro*, thereby shutting off a pathway that tumors use for immune evasion and survival (183). Through STAT3 inhibition, capsaicin can reduce the expression of immunosuppressive factors and increase cancer cell visibility to the immune system. This aligns with reports that capsaicin's antitumor efficacy involves enhancing cytotoxic T lymphocyte activity and promoting an immunogenic tumor microenvironment (184). Capsaicin and piperine may thus act as immune modulators, reducing the chronic inflammation that drives many cancers while boosting antitumor immune responses.

#### 3.9.4 Amide phytochemicals suppress angiogenesis and metastasis in cancer

In addition, amide phytochemicals interfere with angiogenesis and metastasis, which are critical processes for cancer progression. Capsaicin has shown remarkable anti-angiogenic activity by inhibiting the formation of new blood vessels that supply tumors. In both cultured endothelial cells and animal tumor models, capsaicin treatment reduced VEGF production and blocked angiogenic signaling (185). One study found that capsaicin activated a p53-SMAR1 autoregulatory loop in lung cancer cells, leading to the downregulation of VEGF and subsequent impairment of angiogenesis (185). Correspondingly, capsaicin suppressed *in vitro* tube formation of endothelial cells and *in vivo* microvessel sprouting in implanted tumors (182). This results in nutrient deprivation of the tumor and inhibited growth of secondary lesions. These amides also impede metastatic spread. Capsaicin targets multiple steps of the metastatic cascade by reducing cancer cell motility, invasion, and colony formation at distant sites. Mechanistically, capsaicin downregulates MMP-9 and MMP-2, enzymes that degrade extracellular matrix barriers, by suppressing key signaling pathways such as AMPK–NF-κB, EGFR–FAK/Akt, PKC/Raf/ERK, and p38 MAPK/AP-1 (186). In melanoma and thyroid carcinoma models, capsaicin's inhibition of PI3K/Akt/Rac1 signaling was identified as a primary reason for its anti-migratory effect (187). Capsaicin also upregulates the tight junction protein claudin-3 and reverses EMT markers in aggressive esophageal cancer cells, thereby restoring epithelial characteristics and reducing invasion (166). *In vivo* evidence from an orthotopic prostate cancer model demonstrated that capsaicin treatment significantly reduced metastatic tumor burden, corroborating its anti-metastatic efficacy in whole organisms (166). Piperine has similarly shown anti-metastatic effects, for instance by inhibiting TGF-β-induced EMT and reducing metastatic lung nodules in breast cancer models (103).

#### 3.9.5 Amides target specific cancer-related miRNA pathways

Capsaicin demonstrates direct miRNA restoration in cancer cells. Multiple studies confirm the upregulation of miR-449a in prostate cancer cells (C4-2 and LNCaP lines), where capsaicin treatment directly increases miR-449a expression. The mechanism involves miR-449a targeting of the androgen receptor (AR)3'UTR, leading to AR degradation and enhanced sensitivity to treatment (102). Piperine exhibits potent epigenetic miRNA regulation. Recent combination studies show that piperine plus thymoquinone treatment increases miR-29c expression 53–58-fold in HepG2 and MDA-MB-231 cells, with target validation showing the downregulation of DNMT3B and HDAC3 (188). The mechanism involves the inhibition of the Wnt/β-catenin pathway and modulation of PI3K/Akt/mTOR signaling (189). Piperlongumine affects multiple miRNAs through ROS-dependent mechanisms. Key targets include the downregulation of miR-27a, miR-20a, and miR-17 through ROS-dependent cMyc repression, leading to the induction of ZBTB10 and ZBTB4 transcriptional repressors(190). In lung cancer studies, miR-34b-3p upregulation targets TGFBR1 (transforming growth factor beta type I receptor) in A549 and H1299 cell lines (191) (Table 1).

**Table 1:** Major classes of functional food ingredients and their principal anti-cancer mechanisms.

**Ingredient class**	**Typical food sources/signature compounds**	**Key molecular targets → principal anticancer actions**	**Representative cancer models**
Polyphenols	Turmeric: curcumin; grapes: resveratrol	NF-κB, IL-6, COX-2 ↓; Bax/Bcl-2 ↑ and caspase cascade → mitochondrial apoptosis; PI3K/Akt/mTOR inhibition → autophagy; reprogramming of the TME (↓ TAM polarization, CAF clearance) (12–18)	Breast, pancreatic; mouse xenografts
Carotenoids	Tomato: lycopene; kelp: fucoxanthin	Redox modulation (selective ROS scavenging/production); NF-κB suppression → anti-inflammatory TME; p21/p27 ↑+ Cyclin ↓ → cell cycle arrest; p53-GADD45α ↑ → DNA repair (31–44)	Prostate, lung, gastric
ω-3 PUFAs	Marine fish oil: EPA/DHA	NF-κB/COX-2 ↓ → inflammation control; lipid-raft remodeling → EGFR-RAS/MAPK and PI3K/Akt inactivation; Akt/ERK phosphorylation ↓ → apoptosis; exosomal miR-21 ↓ → anti-angiogenesis (50, 51, 58–60, 63)	Breast, non-small-cell lung
Phytosterols	Nuts/legumes: β-sitosterol	Membrane-cholesterol depletion → raft signaling blockade; Bax/Bcl-2 ratio ↑ and caspase-3/9 activation → mitochondrial apoptosis; ERK1/2 and NF-κB inhibition (78–83, 92, 93, 97)	Breast, liver
Alkaloids	Coptis rhizome: berberine; yew: paclitaxel	AMPK activation/STAT1–IDO1 and PD-L1 ↓ → immune evasion reversal; β-Tubulin binding → G2/M block; NF-κB inhibition → ROS-driven apoptosis (85, 98–100)	Colorectal, pancreatic, multiple solid tumors
Isothiocyanates (ITCs)	Broccoli: sulforaphane; watercress: PEITC	Covalent BID modification → mitochondrial outer-membrane permeabilization; caspase cascade; G2/M block; NRF2-Keap1 modulation → oxidative-stress response (108, 109, 112, 113)	Colorectal, prostate
Amides	Chili: capsaicin; black pepper: piperine	TRPV1-mediated Ca^2+^ surge and ROS ↑ → Bax/Bcl-2 shift, cytochrome-c release; AMPK/JNK/p53 activation; G1 or G2/M arrest (172–175, 177)	Prostate, pancreatic, glioma

## 4 Synergistic effects of functional food active ingredients and potential for clinical applications

### 4.1 Combination with radiotherapy or chemotherapy: improving efficacy and reducing side effects

Several studies have shown that active ingredients in functional foods can increase tumor sensitivity to radiotherapy and chemotherapy, thereby enhancing therapeutic efficacy (Table 2). Polyphenolic compounds have antioxidant and signaling pathway modulation effects, which can inhibit carcinogen activation, tumor proliferation and metastasis, and downregulate drug resistance-related proteins in cancer cells, thus enhancing the effects of chemotherapeutic drugs and overcoming drug resistance (192). Experiments have shown that combining two polyphenols can produce synergistic effects: When EGCG and curcumin are combined in prostate cancer-resistant cells, they can upregulate the cell cycle blocking protein p21 and induce antitumor responses, which were not observed when either compound was used alone (193). In addition, omega-3 polyunsaturated fatty acids can improve tumor response to treatment by altering cell membrane composition and signaling; in breast cancer models, only when omega-3 fatty acid supplementation is used in combination with chemotherapeutic agents can tumor growth and metastasis be significantly inhibited, resulting in better tumor control compared to the use of drugs alone (194). For carotenoids, some studies have found that the higher the blood levels of carotenoids in head and neck cancer patients treated with radiotherapy, the longer their progression-free survival, suggesting that the intake of carotenoid-rich foods may help improve long-term outcomes after treatment (195).

**Table 2:** Synergistic combinations between functional-food bioactives and conventional cancer therapies.

**Functional bioactive (class)**	**Partner therapy (chemo/RT/immuno)**	**Key sensitization/protection mechanism**	**Representative outcome (model/study)**
Curcumin (polyphenol)	5-Fluorouracil	NF-κB suppression → Bax/Bcl-2 shift; thymidylate-synthase downregulation	IC_50_ of 5-FU ↓≈ 8-fold in HCT-116 xenografts; tumor volume ↓ 75 %
EGCG (green-tea catechin)	5-Fluorouracil	MDR1 and MRP2 downregulation; autophagy induction	Multidrug-resistant HT-29 cells resensitized (viability ↓> 60 %)
Sulforaphane (ITC)	Docetaxel	CSC depletion (ALDH^1+^, CD44^+^/α2β1^+^ ↓); NF-κB inhibition	DU-145 xenograft growth delay doubled vs. docetaxel alone
Resveratrol (polyphenol)	Doxorubicin	Topo-IIα stabilisztion; ROS buffering → cardioprotection while promoting tumor ROS burst	MCF-7 tumors: apoptosis ↑ 2 × ; rat hearts: CK-MB leakage ↓ 50 %
DHA/EPA (ω-3 PUFAs)	Cisplatin and paclitaxel	Lipid-raft remodeling → EGFR/PI3K-Akt inactivation; oxidative DNA damage ↑	A549 xenografts: cisplatin tumor inhibition ↑ 45 %, nephrotoxicity unchanged
Lycopene (carotenoid)	Radiotherapy (γ-ray)	ROS amplification and DNA double-strand breaks; G2/M arrest	TRAMP mice: PSA-normalized tumor weight ↓ 40 % with 2 Gy fractions (195, 196)
Gallic acid (phenolic acid)	γ-Irradiation	p53 and JNK activation → mitochondrial apoptosis; GADD45α ↑	HepG2 cells: combined treatment apoptosis rate ↑ 3 × vs. either agent (161)
Piperine (alkaloid)	TRAIL ligand	Death receptor upregulation (DR4/DR5); caspase-8 activation	HeLa xenograft regression (complete in 4/10 mice); normal cells spared (103)

Functional food components also have the ability to mitigate the toxic side effects of radiotherapy. The mechanisms include antioxidant, anti-inflammatory, and normal cell protection. Omega-3 fatty acids have anti-inflammatory effects, and in a clinical trial of lung cancer patients, omega-3 supplementation significantly reduced the levels of inflammatory factors such as CRP and IL-6 in the blood of patients during chemotherapy while improving body weight, albumin, and other nutritional indicators, which helped alleviate the systemic inflammatory response and malnutrition caused by chemotherapy (63). The powerful antioxidant properties of polyphenols and carotenoids can scavenge excess free radicals generated by radiotherapy and reduce oxidative stress, thereby protecting normal tissue cells from damage (196). These effects have been demonstrated clinically: The use of EGCG gargle in patients undergoing radiotherapy for head and neck cancer significantly reduced radiation therapy-induced inflammation and ulcerative damage to the oral mucosa (197). Another example is a study of 540 patients with head and neck cancer, which found that those with lower dietary β-carotene intake during radiotherapy were more likely to experience severe side effects and tumor recurrence; on the contrary, the high intake group had a significantly lower incidence of severe acute side effects (OR = 0.61) and a lower risk of regional tumor recurrence (196). It can be seen that the combined application of these functional food components with radiotherapy can enhance tumor treatment and improve efficacy, as well as help alleviate treatment-related adverse effects and protect the patient's body functions.

### 4.2 Cancer rehabilitation and long-term prevention: long-term benefits of dietary interventions

During the rehabilitation phase after tumor treatment, rational dietary interventions and functional food intake are crucial for reducing the risk of cancer recurrence and improving patient survival quality. Studies have shown that cancer survivors who maintain high-quality dietary patterns have a significantly better long-term prognosis than those with poor dietary quality. A large systematic review and meta-analysis summarizing the results of 117 studies found that cancer survivors with the highest adherence to the Mediterranean diet had a risk of all-cause mortality that was ~25% lower than those with the lowest adherence (RR = 0.75) (198). Studies of breast cancer survivors have reached similar conclusions: adherence to the Mediterranean diet significantly reduces mortality in breast cancer patients (HR ≈ 0.78) (199). Additionally, dietary interventions may also help reduce tumor recurrence. In the wellknown WINS randomized controlled trial, early-stage breast cancer patients who received low-fat dietary interventions experienced an approximately 24% reduction in cancer recurrence compared to the control group (200). In contrast, another trial that only increased fiber intake from fruits and vegetables without controlling calories (the WHEL study) did not observe a significant reduction in relapse risk, possibly due to the lack of weight change in this intervention (201). These results suggest that nutritional interventions are more effective when combined with measures such as weight management—in the WINS trial, patients in the diet group experienced an average weight decrease, while no weight change was seen in the WHEL trial, which is considered a key factor in the difference between the two outcomes. Therefore, the current consensus is to combine healthy eating with physical activity as a comprehensive lifestyle intervention during cancer recovery, aiming to simultaneously reduce the risk of recurrence and improve patients' quality of life (202). By promoting a healthy weight and enhancing metabolism through a controlled diet, patients often experience reduced fatigue and improved body functioning, thereby enhancing both physical and mental health.

Long-term adherence to functional foods and holistic dietary patterns in healthy populations can help reduce the risk of many cancers. Nutritional epidemiology studies have shown that anti-inflammatory diets, such as the Mediterranean-style diet—which is rich in vegetables, fruits, whole grains, and unsaturated fats—may play a role in cancer prevention by reducing chronic inflammation and oxidative stress. The latest systematic review, which combined data from millions of people, found that cancer incidence and mortality rates were significantly lower in people with high adherence to the Mediterranean diet compared to those with low adherence. Specifically, the overall risk of cancer death was 13% lower in the group with the highest compliance with the Mediterranean diet than in the group with the lowest compliance (RR ≈ 0.87), and the risk reduction was more pronounced for certain gastrointestinal tumors, such as colorectal cancer (approximately 17%), gastric cancer (30%), and liver cancer (36%) (198). Such diets are rich in polyphenolic antioxidants and dietary fiber, which help scavenge potentially cancer-causing free radicals and regulate intestinal flora, thus explaining their benefits for cancer prevention. Additionally, following a healthy diet is often associated with a healthier lifestyle, which can reduce the occurrence of cancer risk factors such as obesity. In summary, sustained dietary interventions are not only beneficial for cancer survivors but are also an important strategy for long-term cancer prevention in the general population. Authoritative guidelines have recommended a plant-based dietary pattern, represented by the Mediterranean diet, to reduce the risk of cancer and other chronic diseases.

### 4.3 Precision nutrition in cancer therapy: genomic-, microbiome-, and multi-omic-guided dietary interventions

#### 4.3.1 Genetic profiling drives personalized dietary recommendations through validated clinical pathways

The LIBRE-1 study provides the most comprehensive evidence for genetically informed nutritional interventions, involving 366 BRCA1/2 mutation carriers in a multicenter randomized controlled trial (203). Participants following Mediterranean diet education showed significant reductions in IGF-I levels and improved adherence to protective dietary patterns, with reduced consumption of red meat and processed foods while increasing whole grains, legumes, and nuts (203). This first large-scale intervention demonstrated the clinical feasibility of genetic risk-based nutritional recommendations for cancer prevention. MTHFR gene polymorphisms have emerged as critical biomarkers for precision nutrition, with the C677T variant present in 47% of Hispanic and 36% of European populations (204). Individuals with the TT genotype require 1.5–2 times higher folate intake (600–800 μg daily) due to a 70% reduction in enzyme activity (205), as validated in the China Stroke Primary Prevention Trial involving 16,413 participants. The clinical significance extends beyond supplementation, with MTHFR expression levels correlating with immune infiltration and checkpoint blockade response across 44 cancer types (206). TP53 tumor suppressor gene interactions represent another validated target for personalized nutrition. The rs1042522 G allele is associated with a significantly increased breast cancer risk (OR = 2.98, *p* < 0.001), while carriers show differential responses to antioxidant supplementation (204). Ongoing clinical trials are testing selenium and vitamin E combinations specifically in TP53 variant carriers, demonstrating the movement from association studies to targeted therapeutic interventions.

#### 4.3.2 Microbiome analysis enables precision dietary interventions with measurable clinical outcomes

The largest multicenter microbiome study in cancer involved 438 melanoma patients across the UK, Netherlands, and Spain, identifying three bacterial species consistently associated with enhanced immunotherapy response: *Bifidobacterium pseudocatenulatum, Roseburia* spp., and *Akkermansia muciniphila* (207). Survival outcomes nearly doubled between high vs. low beneficial microbe subgroups, with microbiome composition influenced by dietary fiber intake, proton pump inhibitor use, and individual constitutional factors. Strain-level microbiome analysis has proven superior to species-level profiling for clinical applications. A Phase 2 trial with 106 diverse cancer patients demonstrated that strain-resolved analysis improved machine learning predictions of immunotherapy response and 12-month progression-free survival compared to conventional taxonomic approaches (208, 209). This precision enables cancer type-agnostic but therapy-specific treatment selections based on individual microbial signatures. High-fiber dietary interventions show remarkable specificity in their microbiome effects. Studies demonstrate that a daily fiber intake of ≥20 g without commercial probiotics produces optimal immunotherapy response rates through enhanced short-chain fatty acid production (210). Fiber-derived butyrate, propionate, and acetate enhance antitumor T-cell responses through SCFA-mediated histone deacetylase inhibition, providing mechanistic validation for microbiome-guided dietary recommendations (211, 212).

#### 4.3.3 Emerging technologies integrate AI and multi-omic data for personalized interventions

Artificial intelligence applications in precision nutrition have achieved remarkable sophistication, with deep learning architectures using convolutional neural networks with up to 295 layers achieving 86% accuracy in food recognition and nutritional analysis (213, 214). The Diet Engine platform combines computer vision, natural language processing, and continuous glucose monitoring for real-time personalized dietary recommendations tailored to cancer treatment protocols. Multi-omic data integration platforms represent the cutting edge of precision nutrition technology. The MintTea platform uses canonical correlation analysis extensions to identify disease-associated modules that combine genomics, microbiome, and metabolomics data, demonstrating effectiveness in colorectal cancer studies. These platforms achieve high predictive power through significant cross-omic correlations, enabling unprecedented personalization of nutritional interventions (215). The European Union's ONCOBIOME network exemplifies international collaboration in data integration, combining gut microbiome, metabolism, and immune response data across multiple cohorts and cancer types. This infrastructure supports the development of oncomicrobiome signatures for diagnosis, prognosis, and treatment response prediction (216).

#### 4.3.4 Gene–diet interactions reveal specific therapeutic targets with clinical applications

BRCA-associated nutrient interactions demonstrate clinically actionable gene–diet relationships. Moderate vitamin B12 supplementation may provide a 20–40% reduction in breast cancer risk for BRCA mutation carriers, with genetic testing guiding supplementation recommendations. Components of olive oil suppress HER2 expression through epigenetic mechanisms, particularly benefiting BRCA carriers at risk for HER2-positive breast cancer through Mediterranean diet patterns that include 3–4 tablespoons of daily extra virgin olive oil. The GECCO Consortium analysis of 9,287 colorectal cancer cases identified the rs4143094 variant near the GATA3 gene as significantly modifying processed meat cancer risk (217). This discovery enables risk stratification for dietary counseling based on genotype, demonstrating how large-scale genetic studies translate into personalized dietary recommendations. Immunotherapy–nutrition interactions represent an emerging area of precision application. Studies in melanoma and lung cancer patients focus on fatty acid metabolism genes (FASN, FADS1/2), with omega-3 fatty acid supplementation and fiber intake optimization based on genetic variants enhancing the efficacy of immune checkpoint inhibitors. These findings support the integration of precision nutrition with immunotherapy protocols.

#### 4.3.5 Machine learning enhances precision through predictive algorithms and real-time optimization

Advanced machine learning models integrate clinical, microbiome, and dietary data for treatment selection with impressive accuracy. Autonomous AI agents achieve 87.5% accuracy in using appropriate clinical tools for oncology decision-making, while neural network models predict disease risk from 168 circulating metabolic markers in large population studies (214). The Food4Me European study extension demonstrates practical AI implementation, involving 1,607 participants across seven countries with genetic testing for FTO, APOE, MTHFR, and TCF7L2 variants (218). Genotype-based recommendations resulted in 1.5–2 times greater dietary behavior changes compared to general advice, validating the clinical utility of AI-driven personalized nutrition. Real-time biomarker monitoring through wearable devices enables continuous optimization of interventions. Kaiku Health's platform serves over 64,000 patients across more than 40 European cancer clinics, achieving patient recommendation rates of 95–99% and improved care perception of 86–90%. The platform's machine learning algorithms predict symptom onset and progression while reducing the healthcare system's phone call burden by 57%.

## 5 Opportunities and challenges

### 5.1 Bioavailability and pharmacokinetic limitations

#### 5.1.1 Fundamental bioavailability barriers limiting functional food efficacy

The primary barriers restricting bioactive compound absorption operate through interconnected mechanisms that significantly reduce therapeutic potential. Five critical limitations dominate across compound classes: extensive first-pass metabolism, poor solubility characteristics, gastrointestinal absorption barriers, chemical instability, and active efflux transport. First-pass metabolism presents the most significant barrier for most bioactive compounds. Polyphenols undergo rapid glucuronidation via UDP-glucuronosyltransferases (UGT1A1, UGT1A8, UGT1A9), with up to 95% of absorbed compounds being conjugated before reaching systemic circulation (219). This process occurs both in intestinal enterocytes and hepatic tissue, creating dual metabolic barriers (220). Recent studies demonstrate that men show four-fold higher UGT2B17 expression than women, explaining significant gender differences in bioavailability patterns (221).

Solubility limitations create dissolution rate bottlenecks that constrain absorption. Most polyphenol aglycones exhibit water solubility below 20 μg/mL (222), while carotenoids require micellization with dietary fats for absorption, showing three- to five-fold higher bioavailability when consumed with fatty meals (223). Phytosterols face dual solubility challenges, being poorly soluble in both water and oils despite structural similarities to cholesterol, which achieves 56% absorption compared to phytosterols' 0.5–2% (224). Gastrointestinal absorption barriers include limitations of tight junctions, diffusion constraints of the mucus layer, and pH-dependent degradation. The colon mucus layer reaches a thickness of 830 μm, creating significant diffusion barriers for large molecules. Additionally, anthocyanins demonstrate particular instability at neutral pH, losing both color and bioactivity through rapid degradation mechanisms. Efflux pump interactions, particularly through P-glycoprotein (ABCB1/MDR1), actively limit compound absorption by recognizing a wide range of hydrophobic bioactive substances and using ATP-dependent transport to expel them from enterocytes back into the intestinal lumen. ABCG5/G8 transporters specifically target phytosterols, explaining their extremely low bioavailability despite their structural similarity to readily absorbed cholesterol (225). Genetic polymorphisms, particularly rs1045642 (3435T>C), significantly affect pump activity, with TT genotypes showing higher extrusion capacity.

#### 5.1.2 Nanodelivery technologies revolutionizing bioactive compound absorption

Nanodelivery systems address bioavailability limitations through sophisticated mechanisms that enhance solubility, protect compounds from degradation, improve intestinal absorption, and bypass first-pass metabolism (226). Four primary technologies dominate current applications: liposomes, nanoparticles, nano-emulsions, and emerging nanovesicular systems. Liposomes provide versatile encapsulation platforms through phospholipid vesicles that accommodate both hydrophilic and lipophilic compounds. Recent advances include enhanced PEGylation strategies for improved circulation time, stimuli-responsive systems for targeted release, and novel surface modifications using ligand conjugation for active targeting. Encapsulation occurs through passive loading during formation, active loading using pH gradients, or direct lipid bilayer integration for lipophilic compounds. The enhanced permeation and retention (EPR) effect enables passive targeting through leaky vasculature, while active targeting utilizes antibody or peptide recognition systems.

Solid lipid nanoparticles (SLNs) and nanostructured lipid carriers (NLCs) offer controlled release capabilities with superior stability profiles. SLNs utilize solid lipid cores stabilized by surfactants, achieving 28–81% encapsulation efficiency for compounds like curcumin. NLCs improve upon SLN technology by incorporating liquid lipids that create imperfect crystal structures, enabling higher drug loading capacity and reduced compound expulsion during storage. Recent innovations include supercritical fluid production methods and hybrid polymer–lipid systems that combine the benefits of both platforms. Nano-emulsions deliver exceptional stability and enhance bioavailability through systems typically ranging from 10 to 100 nm in particle size (227). Oil-in-water formulations dominate food applications, while Pickering emulsions utilize solid particle stabilization for enhanced stability (228). Recent formulation advances include OSA-modified starch as novel emulsifiers and ultrasonic-assisted formation techniques that increase encapsulation efficiency by 15–20%. These systems enhance bioavailability through multiple mechanisms: particle size reduction increases surface area-to-volume ratios, amorphous state formation prevents crystallization, and surface modification improves wettability.

Protection from degradation occurs through physical barrier effects, antioxidant co-incorporation, controlled microenvironment maintenance, and thermal stability during processing (229). Improved intestinal absorption results from enhanced permeation through tight junction modulation, lymphatic targeting via lipid-based systems, receptor-mediated endocytosis, and size-dependent paracellular transport. Bypassing first-pass metabolism utilizes lymphatic delivery for direct access to systemic circulation, targeted release for site-specific delivery, and sustained release to reduce metabolic clearance.

#### 5.1.3 Eutectic and co-crystal innovations enhancing compound solubility

Eutectic systems and co-crystal technologies provide alternative approaches for bioavailability enhancement through molecular-level modifications that improve solubility, stability, and dissolution characteristics (230). Deep eutectic solvents (DES) have emerged as particularly promising platforms, formed by combining hydrogen bond acceptors with donors at specific molar ratios to create green, sustainable formulation systems (231). DES enhance bioavailability through sophisticated molecular mechanisms. Extensive hydrogen bonding networks solubilize hydrophobic bioactive compounds through multiple interaction types, including ionic, hydrogen bonding, and van der Waals forces (232). Polarity modulation allows for adjustments in the HBA/HBD ratio and water content to match specific compound requirements (233). Matrix stabilization maintains amorphous forms to prevent crystallization, while permeability enhancement occurs through membrane fluidity modulation.

Recent applications demonstrate remarkable effectiveness across compound classes. Choline chloride-based DES systems achieve two-fold higher quercetin extraction yields compared to conventional solvents. Hydrophobic DES using DL-Menthol and camphor reached 163.5 ± 1.1 mg/100 g carotenoid extraction from orange peels (234). β-Carotene encapsulation in whey protein concentrate using choline chloride-butanediol DES achieved optimal loading capacity, while astaxanthin ester solubility reached 473.63 mg/mL in DES-based microemulsions (234). Co-crystal technologies utilize multi-component crystalline materials formed through non-covalent interactions between bioactive compounds and Generally Recognized as Safe (GRAS) co-formers (235). Formation occurs through hydrogen bonding, π-π stacking interactions, van der Waals forces, and halogen bonding. These systems enhance bioavailability through solubility modulation, supersaturation generation via “spring and parachute” effects, simultaneous improvements in solubility and permeability, and protection of chemical stability (236).

Recent quercetin co-crystal developments include four new systems: quercetin:caffeine, quercetin:caffeine:methanol, quercetin:isonicotinamide, and quercetin:theobromine dihydrate, each demonstrating superior pharmacokinetic properties (237). Machine learning approaches now enable the prediction of flavonoid co-crystal formation, while production using food-grade co-formers has expanded significantly. Solid dispersions represent advanced fourth-generation systems that incorporate both solubility enhancement and controlled release properties. Novel preparation methods include hot melt extrusion with improved parameters, spray drying with optimized carriers, supercritical anti-solvent processes, and electrospraying using DES solvents. Combination carriers like HPMC 4000 with PEG 6000 provide swelling-based dissolution enhancement, while surface-active carriers, including Gelucires and Poloxamers, improve performance characteristics (238) (Table 3).

**Table 3:** Bioavailability barriers of food-derived anticancer agents and proven nano/eutectic solutions.

**Major barrier**	**Key affected compounds (class)**	**Root cause**	**Validated delivery strategy (outcome)**
Poor aqueous solubility and precipitation in the gut	Curcumin (polyphenol); lycopene (carotenoid)	Hydrophobic crystals limit dissolution	•Self-nano-emulsifying formulation of curcumin → 18–30 × higher Cmax •O/W nano-emulsion of lycopene → 5 × oral bioavailability (201–204)
Extensive phase-II conjugation (UGT/SULT first-pass)	Resveratrol (polyphenol); EGCG (catechin)	Rapid glucuronidation/sulfation in enterocytes and liver	•PEG–PLGA nanoparticles of resveratrol → 9 × systemic AUC •EGCG–phospholipid phytosome → 3 × plasma level (205–208)
ABCB1 (P-gp) efflux	Berberine (alkaloid); quercetin (flavonol)	Active pumping back to intestinal lumen	•Berberine solid–lipid nanoparticles bypass efflux → AUC ↑ 6 × •Quercetin nanocrystals (PVP) → efflux ratio ↓ 50% (209–212)
Chemical/enzymatic instability in the GI tract	Sulforaphane (ITC); DHA/EPA (ω-3 PUFAs)	Isothiocyanate hydrolysis; lipid oxidation	•Chitosan–alginate nanocapsules protect sulforaphane (≈ 90% retained after 2 h gastric) •Alginate–maltodextrin microcapsules cut ω-3 peroxide formation by 80% (213–216)
Rapid systemic clearance/short half-life	Curcumin; capsaicin (amide)	High hepatic clearance, wide tissue distribution	•PEGylated polymeric micelles: curcumin t$\frac{1}{2}$ 4 → 12 h •Liposome-encapsulated capsaicin: t$\frac{1}{2}$ 1.5 → 6 h (217–219)
Low dissolution of crystalline solids	Curcumin; quercetin	Poor lattice disintegration in GI fluids	•Natural deep-eutectic solvent (choline-glycerol) solubilizes curcumin > 700-fold •Quercetin–menthol eutectic nanoparticles double apparent solubility (220–222)

### 5.2 Dose effect and safety issues

#### 5.2.1 Dose-dependent safety concerns for functional food active ingredients

Emerging evidence reveals significant population-specific toxicity risks for several widely consumed functional food ingredients, with genetic factors, smoking status, and concurrent medications dramatically altering safety profiles (239). Critical safety thresholds have been established for compounds like EGCG (800 mg/day limit) (240) and β-carotene (contraindicated in smokers), while hepatotoxicity cases linked to curcumin formulations have prompted regulatory warnings across multiple jurisdictions. The landscape of functional food ingredient safety has shifted substantially, with precision medicine approaches now essential for risk assessment. Notable developments include identification of HLA-B35:01 genetic susceptibility for hepatotoxicity (241), establishment of regulatory limits based on pharmacogenetic data, and documentation of serious adverse drug–nutrient interactions in vulnerable populations. These findings challenge the traditional “natural equals safe” paradigm and necessitate evidence-based dosing recommendations.

#### 5.2.2 Compound-specific toxicity thresholds and safety profiles

##### 5.2.2.1 Curcumin: hepatotoxicity emergence and bioavailability concerns

Recent evidence has highlighted emerging hepatotoxicity concerns associated with curcumin, particularly in the context of enhanced bioavailability formulations. While conventional curcumin has demonstrated a favorable safety profile at doses up to 8,000 mg/day (242), hepatotoxic events have been reported at significantly lower doses when co-administered with absorption enhancers such as piperine. The European Food Safety Authority (EFSA) has established an acceptable daily intake (ADI) of 3 mg/kg body weight (~210 mg/day for adults) as a conservative threshold (243). Notably, individuals carrying the HLA-B^*^35:01 allele—found in 10–15% of the population—appear to be genetically predisposed to curcumin-induced liver injury. These individuals may experience abrupt elevations in alanine aminotransferase (ALT) levels exceeding 1,000 U/L within 1–4 months of use, often accompanied by autoimmune hepatitis-like features. The hepatotoxicity is typically reversible upon drug withdrawal, supporting an immune-mediated mechanism rather than dose-dependent toxicity (244). Mechanistically, the risk is attributed to the pharmacokinetic impact of piperine, which inhibits CYP3A4 and disrupts glucuronidation, increasing systemic curcumin exposure by over 20-fold and potentially converting otherwise safe doses into hepatotoxic levels in susceptible individuals.

##### 5.2.2.2 EGCG: established hepatotoxicity with regulatory response

Emerging hepatotoxicity concerns have prompted regulatory action on epigallocatechin gallate (EGCG), particularly in concentrated supplement form. In 2023, the European Union established a mandatory upper limit of 800 mg/day for EGCG in dietary supplements, following safety evaluations that revealed transaminase elevations in nearly a quarter of intervention trials at doses ≥800 mg/day. The European Food Safety Authority (EFSA) further set a conservative acceptable daily intake (ADI) of 322 mg/day for adults. Genetic susceptibility plays a critical role, with HLA-B\^*^35:01 carriers accounting for 72% of reported liver injury cases compared to just 10–15% in the general population. Data from the Minnesota Green Tea Trial showed a 6.7% incidence of ALT elevation in postmenopausal women consuming 843 mg/day EGCG, with polymorphisms in the COMT and UGT1A4 genes contributing to interindividual variability in toxicity (245). Mechanistically, EGCG promotes mitochondrial dysfunction and ROS accumulation in hepatocytes, depleting intracellular glutathione and triggering apoptosis via caspase-3 activation. Notably, ingestion in the fasting state markedly increases EGCG bioavailability and amplifies hepatotoxic risk, prompting regulatory mandates for warning labels on supplement packaging.

##### 5.2.2.3 β-carotene: smoking-specific lung cancer risk

β-Carotene supplementation has been associated with increased cancer risk in specific populations, particularly current smokers. Meta-analyses published between 2022 and 2024 consistently report an 18–24% elevation in lung cancer incidence among smokers receiving β-carotene supplements, a finding corroborated by large-scale trials such as ATBC and CARET (246). Notably, the observed harm appears independent of dosage—typically 20–30 mg/day—suggesting a threshold-independent mechanism. This phenomenon is explained by the Burton–Ingold hypothesis, whereby β-carotene shifts from an antioxidant to a pro-oxidant under high oxygen tension, as found in the pulmonary environment of smokers. This mechanistic switch leads to oxidative DNA damage and enhanced carcinogenesis, accounting for the population-specific toxicity (246). In response, current clinical guidelines strictly contraindicate β-carotene supplementation in active smokers and individuals with a history of asbestos exposure (246). Evidence for former smokers remains inconclusive, with some studies indicating a persistent elevated risk, underscoring the need for cautious individualized assessment.

##### 5.2.2.4 Omega-3 fatty acids: bleeding risk reassessment

Recent safety reassessments have refined the bleeding risk profile of omega-3 fatty acid supplementation. A 2024 meta-analysis encompassing 120,643 patients found no significant increase in overall bleeding risk with standard omega-3 formulations (relative risk 1.09; 95% CI 0.91–1.31), reinforcing the safety of conventional EPA/DHA combinations up to 4,000 mg/day (247). However, high-dose purified EPA formulations (≥4,000 mg/day EPA alone) were associated with a 50% relative increase in bleeding events, though this translated to a modest absolute risk increase of only 0.6% (247). Mechanistically, EPA and DHA integrate into platelet membranes and displace arachidonic acid, thereby shifting the eicosanoid balance toward increased prostacyclin (PGI_2_) and reduced thromboxane A_2_ (TXA_2_) synthesis (248). This creates modest antiplatelet effects similar to low-dose aspirin but generally without clinically significant bleeding at therapeutic doses. Nonetheless, in patients receiving anticoagulants or antiplatelet agents, high-dose EPA formulations warrant enhanced monitoring to mitigate potential additive bleeding risk.

#### 5.2.3 Current regulatory frameworks and safety assessments

The European Union has implemented the most comprehensive regulatory framework for functional food ingredients. Commission Regulation EC 2023/2006 establishes mandatory Good Manufacturing Practice requirements for food contact materials, while updated EFSA tolerance frameworks provide systematic approaches to determining upper intake levels. EFSA scientific opinions from 2022 to 2025 have established ADI values for multiple compounds: curcumin (3 mg/kg body weight) (243), resveratrol (150 mg/day maximum), and tetrahydrocurcuminoids (2 mg/kg body weight). These assessments incorporated margin of exposure calculations that consider vulnerable populations, particularly pregnant women and children. Harmonized labeling requirements now mandate warnings for EGCG-containing products, including contraindications for pregnancy, lactation, children under 18, and fasting consumption. These evidence-based warnings represent a paradigm shift toward population-specific safety communication.

The FDA has expanded qualified health claims for omega-3 fatty acids related to hypertension and coronary heart disease risk reduction while maintaining GRAS status for most functional ingredients (243). However, warning letters continue to be issued for unauthorized disease claims, particularly for curcumin-based products. Small Entity Compliance Guides provide enhanced clarity for omega-3 nutrient content claims, while discussions on modernizing 21 CFR Part 111 dietary supplement regulations are ongoing. The FDA has not established specific maximum daily intake limits for most compounds, relying instead on GRAS provisions and post-market surveillance. Health Canada's Supplemented Foods Regulations entered a transition phase through December 2025, requiring premarket assessment for novel ingredients. The TMAL system phase-out signifies a move toward more rigorous safety evaluation protocols. FSANZ updated dietary exposure assessment principles in 2024, emphasizing the integration of food chemical concentration and consumption data for regulatory decision-making. These frameworks enable more sophisticated risk assessments that incorporate population-specific consumption patterns.

### 5.3 The challenge of clinical translation

#### 5.3.1 Lack of large-scale randomized controlled trials (RCTs)

The potential of functional food active ingredients in combating conditions such as cancer has received widespread attention at the *in vitro* and animal levels, but high-quality human evidence is relatively scarce. Many studies remain at the stage of small-sample clinical trials or observational studies, and there is a lack of rigorous large-scale RCTs to validate their efficacy. In the case of curcumin, for example, although basic research has been fruitful, clinical trial results have not been adequately followed up. A review investigated curcumin-related studies in the ClinicalTrials database and found that < 10% of the nearly 300 clinical studies registered had published results, and these trials generally suffered from small sample sizes, a single disease type, and inconsistent intervention protocols. More importantly, few studies published results, meaning that many trials failed to produce clear conclusions (249). Overall, there is still a lack of convincing evidence for the clinical efficacy of functional food actives, and large-scale, high-quality RCTs are urgently needed to facilitate the translation of these actives from the laboratory to clinical applications.

#### 5.3.2 Regulatory approvals and standardization issues

Functional foods and dietary supplements are regulated differently across countries and regions, which significantly impacts their marketability and clinical application. Inconsistency in regulatory frameworks and the lack of standards may result in uneven product quality and exaggerated claims. In the United States, such products are categorized as dietary supplements and are not subject to the same rigorous validation of efficacy as drugs, with regulation focusing on safety rather than efficacy. This relatively lax regulatory framework has facilitated commercialization but also raised quality concerns (250). On the contrary, Japan, Korea, and other countries have established specialized approval systems for functional foods (e.g., FOSHU certification for “Foods for Specific Health Uses”), requiring the submission of sufficient scientific evidence to support health claims and implementing strict controls before and after marketing. The European Union has also formulated guidelines for the safety assessment of plant extracts as food additives and audited the functional claims, raising the threshold for market entry while ensuring safety (251). As regulations and standards vary from country to country, companies face approval and compliance challenges when promoting such products in different markets. A survey found that about 59% of curcumin supplements marketed in the U.S. did not meet active ingredient content standards, and many contained residual solvents, reflecting the difficulty of maintaining consistent product quality under the current regulatory system. Therefore, to promote the clinical translation of functional food active ingredients, not only is more scientific evidence needed to support their efficacy, but there is also a call for unified standardized quality standards and regulatory policies to ensure safety and effectiveness while accelerating product compliance in the market.

## 6 Future directions

### 6.1 Novel drug delivery systems (nanodelivery, co-delivery systems)

#### 6.1.1 Nanodelivery technology to enhance bioavailability

Functional food active ingredients often suffer from poor water solubility, easy degradation, and low bioavailability. In recent years, nanotechnology has provided new methods for their delivery. By constructing nanocarriers, active ingredients can be protected from damage in the digestive tract environment, reducing toxicity and increasing their absorption rate in the gastrointestinal tract, thus significantly improving bioavailability. Some studies have encapsulated phenolic antioxidants in nanoliposomes, which not only effectively maintain the stability of the active ingredients but also improve their bioavailability (252). Similarly, the delivery of unstable nutrients using nanostructured carriers has yielded good results in enhancing their stability in food systems and extending shelf life (253). Notably, novel materials such as plant proteins are also being used as nanocarriers to develop edible and safe delivery systems. However, there are still concerns about the large-scale application of nanodelivery in food, as the long-term safety of nanomaterials *in vivo* remains unclear (254). Future research needs to focus on the safety assessment and biocompatibility improvement of nanocarriers to ensure that efficacy is enhanced without introducing new safety risks.

#### 6.1.2 Co-delivery of functional ingredients and drugs

Delivering functional food active ingredients together with traditional chemotherapeutic drugs via nanocarriers is another innovative strategy to improve anticancer efficacy. Studies have demonstrated that the combination of natural products and chemotherapeutic drugs can exert synergistic antitumor effects through multiple mechanisms: in addition to directly inhibiting tumor cell proliferation and inducing apoptosis, it can also overcome multidrug resistance (MDR) and reduce the toxic side effects of chemotherapy. Nanodelivery co-delivery system (NDCDS) allows two active substances to be encapsulated in the same nanocarrier, delivered simultaneously to the tumor site, and released in a designed ratio and time sequence, resulting in synergistic effects within the tumor cells (255). Co-loading plant anticancer components such as quercetin and curcumin with multiple chemotherapeutic agents in nanocarriers significantly enhanced apoptosis induction and proliferation inhibition of tumor cells. This type of nano-co-delivery strategy has demonstrated superior anticancer activity over single drugs *in vitro* and in animal models. Looking forward, combined nanodelivery of functional food active ingredients with chemotherapeutic agents is considered a promising anticancer modality that can simultaneously improve efficacy and reduce the occurrence of drug resistance and toxicity. According to the review, nano-co-delivery of natural products with chemotherapeutic agents is expected to be the basis of new tumor therapeutic strategies that facilitate the development and translation of anticancer drugs (256). Although currently mostly in preclinical studies, this co-delivery strategy is expected to gradually move toward clinical application as delivery systems improve and more pharmacokinetic/toxicological data accumulate (Table 4).

**Table 4:** Key translational barriers for functional-food anticancer agents and practical paths forward.

**Challenge**	**Clinical/commercial impact**	**Actionable solution(s)**	**Current progress/outlook**
Poor oral bioavailability (low solubility, rapid metabolism, efflux)	High: limits efficacious plasma/tumor levels	• Nano-emulsions, polymeric micelles, phytosomes • Natural deep-eutectic solvents • Co-crystal and self-emulsifying tablets	Multiple phase-I nutraceuticals with ≥10-fold AUC gains; two nano-curcumin products in pilot oncology trials (IND stage) (223–226)
Batch-to-batch phytochemical variability (source, season, processing)	High: undermines dosing accuracy and reproducibility	• GMP-grade botanical extracts with chemoprofiling (HPLC-MS) • Barcode genomics for raw material authentication	ISO 19614 phytochemical QC adopted in EU and China; NIH NCCIH Herbal Chemical Marker List expanding (227–229)
Safety signals and drug–nutrient interactions	Moderate–High: risk of off-target toxicity and chemotherapy interference	• Population-stratified dosing • Therapeutic drug-monitoring dashboards • *In silico* PK/PD interaction modeling integrated into EHR	FDA Botanical Guidance v2 (2023) emphasizes interaction data; early-phase trials increasingly include CYP and transporter panels (230–232)
Regulatory fragmentation across regions	Moderate: slows global market entry	• Harmonized monograph dossiers (ICH M4Q-like) • Mutual recognition of safety/effectiveness evidence	ASEAN TMHS Act (2024) moving toward common standard; WHO “Herbal Medicine Priority List” in draft (233, 234)
Cost-effectiveness/scalability	Moderate: large-scale extraction and nano-formulations raise price	• Agro-industrial by-product valorization • Continuous-flow nano-encapsulation • Public–private manufacturing consortia	Swiss RCT (2022) showed CHF 129 nutritional support averts CHF 1 788 complications; continuous-flow lines now cGMP-ready (235–237)
Patient compliance and diet-lifestyle fit	Moderate: long-term adherence dictates outcome	• Once-daily high-load capsules/functional food bars • Digital adherence apps with symptom tracking • Personalized nutrition counseling	Two ω-3 “medical food” bars received US CMS reimbursement (2025); app-linked adherence ↑ 30 % in pilot (238, 239)
Heterogeneous clinical evidence and small sample sizes	High: weakens guideline adoption	• Multicenter adaptive platform trials (basket designs) • Real-world data registries linking diet logs + outcomes • Bayesian evidence synthesis across preclinical and clinical data	First adaptive trial on sulforaphane + docetaxel launched 2024 (*N* = 400, five tumor baskets); NIH Nutrition for Precision Oncology registry live (240–242)
Limited intellectual-property incentives	Moderate: deters pharma-scale investment	• Composition-of-matter patents on nanodelivery systems • Regulatory data exclusivity for “novel-food” indications • Public grant matchmaking with SME formulators	2023 EU Orphan Nutritionals scheme offers 10-year data exclusivity; >50 nano-phytochemical patents filed 2022–25 (243, 244)

High = barrier is predicted to preclude, delay, or severely limit clinical adoption unless specifically mitigated; moderate = barrier is expected to slow or narrow adoption but can be addressed with focused R&D, regulatory, or implementation efforts.

AUC, area under the plasma concentration–time curve; ASEAN, Association of Southeast Asian Nations; CMS, Centers for Medicare and Medicaid Services; EHR, electronic health record; FDA, U.S. Food and Drug Administration; GMP, good manufacturing practice; HPLC–MS, high-performance liquid chromatography–mass spectrometry; ICH, International Council for Harmonization of Technical Requirements for Pharmaceuticals for Human Use; IND, investigational new drug; ISO, International Organization for Standardization; NCCIH, National Center for Complementary and Integrative Health; NIH, National Institutes of Health; PCC, Pharmaceutical Cooperation Committee; PK/PD, pharmacokinetic/pharmacodynamic; RCT, randomized controlled trial; SME, small- and medium-sized enterprise; WHO, World Health Organization.

### 6.2 Interdisciplinary collaboration to promote medicinal transformation of functional foods

#### 6.2.1 Multidisciplinary collaboration to accelerate clinical application

To move from experimental research to the clinical application of functional food active ingredients, close collaboration among the fields of biomedicine, food science, nutrition, pharmacology, and nanotechnology is essential. In fact, the concept of functional food itself is a product of multidisciplinary intersection, integrating the principles of food science, nutrition, and pharmacology to achieve comprehensive health interventions. In future development, interdisciplinary teams can showcase their respective strengths: food science and nanotechnology can provide new carriers and preparation processes to enhance the stability and targeting of the active ingredients; biomedical research can elucidate the molecular mechanisms of action of the active ingredients and their targets; and nutrition and clinical medicine can translate these findings into implementable dietary recommendations or complementary therapeutic programs, which can then be verified in clinical trials. This kind of collaborative innovation “from lab to clinic” will effectively promote the transformation of functional foods into medicinal value. A typical example is the recently proposed concept of “Dietary Oncopharmacognosy”, which integrates resources and methods from natural product chemistry, drug discovery, big data, and nutritional sciences, utilizing multiple sources such as natural product databases, anticancer drug databases, and food histology. The strategy combines resources and methods from natural product databases, anticancer drug databases, and food genomics, using artificial intelligence to design individualized anticancer dietary patterns for clinical testing. Accordingly, the researchers constructed an “anticancer food atlas” to quantify the number of active anticancer molecules contained in each food, which can be used to formulate precise nutritional programs for cancer patients (257). This novel interdisciplinary practice demonstrates that it is possible to make the active ingredients in functional foods into “edible anticancer prescriptions” and accelerate their clinical application through collaboration across different fields.

#### 6.2.2 Potential of active ingredients in future drug development

The active ingredients of functional foods are not only used for dietary interventions but also hold great potential to become lead compounds for new drugs. In the history of anticancer drug development, many landmark drugs (e.g., vincristine, paclitaxel, and artemisinin) were derived from natural products such as plants. This suggests that bioactive molecules from food may be transformed into effective anticancer drugs through established drug development pathways. In recent years, with advances in computational biology and medicinal chemistry, researchers have begun using computational simulation, virtual screening, and artificial intelligence techniques to identify anticancer candidates from natural products (258). These technologies can accelerate the identification of functional food ingredients with pharmacological activity and optimize their structures to enhance activity or reduce toxicity. Through molecular docking and machine learning model screening, new molecules with promising anticancer properties have been identified from natural compounds and validated for activity in cellular and animal models (258). Some functional ingredients have also been used as drug carriers or adjuvants to enhance the efficacy of existing anticancer drugs or mitigate their side effects, further expanding the role of functional food ingredients in the drug system. In summary, multidisciplinary collaboration not only promotes the clinical application of functional food active ingredients but also provides a steady stream of ideas and resources for new drug development. It is foreseeable that more active molecules derived from natural diets will emerge in future drug development pipelines, enriching our stockpile of anticancer weapons.

## 7 Conclusion

Functional food-derived bioactive compounds offer a mechanistically rich, low-toxicity adjunct to mainstream oncology care. This review has mapped their pleiotropic actions—ranging from redox buffering and immune modulation to epigenetic reprogramming—and highlighted encouraging preclinical synergy with radiotherapy, chemotherapy, and immunotherapy. Yet, enthusiasm generated “from bench to mouse” has not been matched by confirmatory evidence “from ward to wallet”. Bridging that translational gulf demands a sharper focus on three interlocking realities: cost-effectiveness, real-world scalability, and sustained patient adherence.

First, future trials must embed formal health-economic evaluations rather than treating them as *post-hoc* add-ons. Randomized designs powered for both clinical and economic endpoints are required to determine whether adding high-purity omega-3 or sulforaphane supplements to standard regimens genuinely lowers total care costs through fewer adverse events, shorter admissions, or reduced supportive-drug use. Without such data, payers are unlikely to reimburse, regardless of biochemical plausibility. The stark shortage of large, well-controlled human studies flagged in this review's Challenges section already signals the urgency of this agenda.

Second, scalability hinges on solving formulation and regulatory bottlenecks. Low aqueous solubility, extensive first-pass metabolism, and active efflux sharply curtail systemic exposure to many phytochemicals, inflating required doses and manufacturing costs. Concurrently, fragmented global regulations permit variable quality and unsubstantiated claims, eroding clinician and consumer trust. Harmonized pharmacopeial monographs, validated biomarker-based batch testing, and adaptive regulatory pathways are prerequisites for mass production that does not compromise safety, potency, or affordability.

Third, even the most elegantly engineered nutraceutical will fail if patients cannot or will not take it daily over months or years. Evidence from Mediterranean-diet cohorts shows that adherence correlates strongly with cancer outcomes but wanes without behavioral support. Digital nutrition platforms, taste-masked formulations, and culturally tailored counseling, therefore, need to accompany efficacy trials, so that “effective in principle” translates into “effective in practice”.

None of these hurdles can be cleared by oncologists working in isolation. We advocate a deliberately interdisciplinary research model that recruits nutrition scientists to optimize dietary matrices, pharmacologists and chemical engineers to refine delivery systems, oncologists to design pragmatic trials, health economists to model budget impact, and behavioral scientists to engineer adherence. Regulatory scientists and industry partners must be engaged early to ensure scale-up and global dissemination. In sum, functional food active ingredients are poised to evolve from “promising supplements” to evidence-based components of integrated cancer care—but only if the next research wave couples mechanistic depth with pragmatic, system-level questions. By aligning interdisciplinary expertise around rigorous clinical-economic endpoints, the field can move decisively from laboratory insight to cost-effective, scalable, and patient-centered oncology practice.

## References

[1] Fogacci F Borghi C Cicero AFG. Functional foods and nutraceuticals to reduce the risk of cardiometabolic disease: where we are, and where we are going. Nutrients. (2024) 16:3152. 10.3390/nu16183152 39339749 PMC11434755

[2] Damián MR Cortes-Perez NG Quintana ET Ortiz-Moreno A Garfias Noguez C Cruceño-Casarrubias CE. Functional foods, nutraceuticals and probiotics: a focus on human health. Microorganisms. (2022) 10:1065. 10.3390/microorganisms10051065 35630507 PMC9143759

[3] Bray F Laversanne M Sung H Ferlay J Siegel RL Soerjomataram I. Global cancer statistics 2022: GLOBOCAN estimates of incidence and mortality worldwide for 36 cancers in 185 countries. CA Cancer J Clin. (2024) 74:229–63. 10.3322/caac.21834 38572751

[4] Marino P Mininni M Deiana G Marino G Divella R Bochicchio I. Healthy lifestyle and cancer risk: modifiable risk factors to prevent cancer. Nutrients. (2024) 16:800. 10.3390/nu16060800 38542712 PMC10974142

[5] Plym A Zhang Y Stopsack KH Delcoigne B Wiklund F Haiman C. A healthy lifestyle in men at increased genetic risk for prostate cancer. Eur Urol. (2023) 83:343–51. 10.1016/j.eururo.2022.05.008 35637041 PMC10279925

[6] Botteri E Peveri G Berstad P Bagnardi V Chen SLF Sandanger TM. Changes in lifestyle and risk of colorectal cancer in the european prospective investigation into cancer and nutrition. Am J Gastroenterol. (2023) 118:702–11. 10.14309/ajg.0000000000002065 36227801

[7] Ang BH Teo SH Ho WK. Systematic review and meta-analysis of lifestyle and reproductive factors associated with risk of breast cancer in Asian women. Cancer Epidemiol Biomarkers Prev. (2024) 33:1273–85. 10.1158/1055-9965.EPI-24-0005 39018331 PMC7617425

[8] Mertens E Barrenechea-Pulache A Sagastume D Vasquez MS Vandevijvere S Peñalvo JL. Understanding the contribution of lifestyle in breast cancer risk prediction: a systematic review of models applicable to Europe. BMC Cancer. (2023) 23:687. 10.1186/s12885-023-11174-w 37480028 PMC10360320

[9] González-Palacios Torres C Barrios-Rodríguez R Muñoz-Bravo C Toledo E Dierssen T Jiménez-Moleón JJ. Mediterranean diet and risk of breast cancer: An umbrella review. Clin Nutr. (2023) 42:600–8. 10.1016/j.clnu.2023.02.012 36893621

[10] Monllor-Tormos A García-Vigara A Morgan O García-Pérez MÁ Mendoza N Tarín JJ. Mediterranean diet for cancer prevention and survivorship. Maturitas. (2023) 178:107841. 10.1016/j.maturitas.2023.107841 37660598

[11] Godos J Guglielmetti M Ferraris C Frias-Toral E Domínguez Azpíroz I Lipari V. Mediterranean diet and quality of life in adults: a systematic review. Nutrients. (2025) 17:577. 10.3390/nu17030577 39940436 PMC11819740

[12] Sufianova G Gareev I Beylerli O Wu J Shumadalova A Sufianov A. Modern aspects of the use of natural polyphenols in tumor prevention and therapy. Front Cell Dev Biol. (2022) 10:1011435. 10.3389/fcell.2022.1011435 36172282 PMC9512088

[13] Zoi V Galani V Lianos GD Voulgaris S Kyritsis AP Alexiou GA. The role of curcumin in cancer treatment. Biomedicines. (2021) 9:1086. 10.3390/biomedicines9091086 34572272 PMC8464730

[14] Li J Fan Y Zhang Y Liu Y Yu Y Ma M. Resveratrol induces autophagy and apoptosis in non-small-cell lung cancer cells by activating the NGFR-AMPK-mTOR pathway. Nutrients. (2022) 14:2413. 10.3390/nu14122413 35745143 PMC9228598

[15] Sharma E Attri DC Sati P Dhyani P Szopa A Sharifi-Rad J. Recent updates on anticancer mechanisms of polyphenols. Front Cell Dev Biol. (2022) 10:1005910. 10.3389/fcell.2022.1005910 36247004 PMC9557130

[16] Zoi V Kyritsis AP Galani V Lazari D Sioka C Voulgaris S. The role of curcumin in cancer: a focus on the PI3K/Akt pathway. Cancers. (2024) 16:1554. 10.3390/cancers16081554 38672636 PMC11048628

[17] Zhang W Zhang R Chang Z Wang X. Resveratrol activates CD8(+) T cells through IL-18 bystander activation in lung adenocarcinoma. Front Pharmacol. (2022) 13:1031438. 10.3389/fphar.2022.1031438 36339614 PMC9630476

[18] Jiang H Wang GT Wang Z Ma QY Ma ZH. Resveratrol inhibits pancreatic cancer proliferation and metastasis by depleting senescent tumor-associated fibroblasts. World J Gastrointest Oncol. (2024) 16:3980–93. 10.4251/wjgo.v16.i9.3980 39350997 PMC11438786

[19] Li D Cao D Cui Y Sun Y Jiang J Cao X. The potential of epigallocatechin gallate in the chemoprevention and therapy of hepatocellular carcinoma. Front Pharmacol. (2023) 14:1201085. 10.3389/fphar.2023.1201085 37292151 PMC10244546

[20] Li F Qasim S Li D Dou QP. Updated review on green tea polyphenol epigallocatechin-3-gallate as a cancer epigenetic regulator. Semin Cancer Biol. (2022) 83:335–52. 10.1016/j.semcancer.2020.11.018 33453404

[21] Romano A Martel F. The role of EGCG in breast cancer prevention and therapy. Mini Rev Med Chem. (2021) 21:883–98. 10.2174/1389557520999201211194445 33319659

[22] Li D Cao D Sun Y Cui Y Zhang Y Jiang J. The roles of epigallocatechin gallate in the tumor microenvironment, metabolic reprogramming, and immunotherapy. Front Immunol. (2024) 15:1331641. 10.3389/fimmu.2024.1331641 38348027 PMC10859531

[23] Zhang J Guo J Qian Y Yu L Ma J Gu B. Quercetin induces apoptosis through downregulating P4HA2 and inhibiting the PI3K/Akt/mTOR axis in hepatocellular carcinoma cells: an *in vitro* study. Cancer Rep. (2025) 8:e70220. 10.1002/cnr2.70220 40347062 PMC12065022

[24] Liu C Rokavec M Huang Z Hermeking H. Curcumin activates a ROS/KEAP1/NRF2/miR-34a/b/c cascade to suppress colorectal cancer metastasis. Cell Death Differ. (2023) 30:1771–85. 10.1038/s41418-023-01178-1 37210578 PMC10307888

[25] Ameer SF Mohamed MY Elzubair QA Sharif EAM Ibrahim WN. Curcumin as a novel therapeutic candidate for cancer: can this natural compound revolutionize cancer treatment? Front Oncol. (2024) 14:1438040. 10.3389/fonc.2024.1438040 39507759 PMC11537944

[26] Martino E D'Onofrio N Balestrieri A Colloca A Anastasio C Sardu C. Dietary epigenetic modulators: unravelling the still-controversial benefits of mirnas in nutrition and disease. Nutrients. (2024) 16:160. 10.3390/nu16010160 38201989 PMC10780859

[27] Cháirez-Ramírez MH de la Cruz-López KG García-Carrancá A. Polyphenols as antitumor agents targeting key players in cancer-driving signaling pathways. Front Pharmacol. (2021) 12:710304. 10.3389/fphar.2021.710304 34744708 PMC8565650

[28] Mierziak J Kostyn K Boba A Czemplik M Kulma A Wojtasik W. Influence of the bioactive diet components on the gene expression regulation. Nutrients. (2021) 13:3673. 10.3390/nu13113673 34835928 PMC8619229

[29] La X Zhang L Li Z Li H Yang Y. (-)-Epigallocatechin gallate (EGCG) enhances the sensitivity of colorectal cancer cells to 5-FU by inhibiting GRP78/NF-κB/miR-155-5p/MDR1 pathway. J Agric Food Chem. (2019) 67:2510–8. 10.1021/acs.jafc.8b06665 30741544

[30] Badehnoosh B Rajabpoor Nikoo N Asemi R Shafabakhsh R Asemi Z. MiRNAs: emerging agents for therapeutic effects of polyphenols on ovarian cancer. Mini Rev Med Chem. (2024) 24:440–52. 10.2174/1389557523666230816090138 37587814

[31] Baeza-Morales A Medina-García M Martínez-Peinado P Pascual-García S Pujalte-Satorre C López-Jaén AB. The antitumour mechanisms of carotenoids: a comprehensive review. Antioxidants. (2024) 13:1060. 10.3390/antiox13091060 39334719 PMC11428676

[32] Didier AJ Stiene J Fang L Watkins D Dworkin LD Creeden JF. Antioxidant and anti-tumor effects of dietary vitamins A, C, and E. Antioxidants. (2023) 12:632. 10.3390/antiox12030632 36978880 PMC10045152

[33] Bas TG. Bioactivity and bioavailability of carotenoids applied in human health: technological advances and innovation. Int J Mol Sci. (2024) 25:7603. 10.3390/ijms25147603 39062844 PMC11277215

[34] Karaköy Z Cadirci E Dincer B. A new target in inflammatory diseases: lycopene. Eurasian J Med. (2022) 54:23–8. 10.5152/eurasianjmed.2022.22303 36655441 PMC11163352

[35] Ba W Xu W Deng Z Zhang B Zheng L Li H. The antioxidant and anti-inflammatory effects of the main carotenoids from tomatoes via Nrf2 and NF-κB signaling pathways. Nutrients. (2023) 15:4652. 10.3390/nu15214652 37960305 PMC10650085

[36] Bohn T Balbuena E Ulus H Iddir M Wang G Crook N. Carotenoids in health as studied by omics-related endpoints. Adv Nutr. (2023) 14:1538–78. 10.1016/j.advnut.2023.09.002 37678712 PMC10721521

[37] DeBenedictis JN Murrell C Hauser D van Herwijnen M Elen B de Kok TM. Effects of different combinations of phytochemical-rich fruits and vegetables on chronic disease risk markers and gene expression changes: insights from the MiBLEND study, a randomized trial. Antioxidants. (2024) 13:915. 10.3390/antiox13080915 39199161 PMC11351619

[38] Terao J. Revisiting carotenoids as dietary antioxidants for human health and disease prevention. Food Funct. (2023) 14:7799–824. 10.1039/D3FO02330C 37593767

[39] Zhang X Zhao WE Hu L Zhao L Huang J. Carotenoids inhibit proliferation and regulate expression of peroxisome proliferators-activated receptor gamma (PPARγ) in K562 cancer cells. Arch Biochem Biophys. (2011) 512:96–106. 10.1016/j.abb.2011.05.004 21620794

[40] Hume S Grou CP Lascaux P D'Angiolella V Legrand AJ Ramadan K. The NUCKS1-SKP2-p21/p27 axis controls S phase entry. Nat Commun. (2021) 12:6959. 10.1038/s41467-021-27124-8 34845229 PMC8630071

[41] Zhuang D Kang J Luo H Tian Y Liu X Shao C. ARv7 promotes the escape of prostate cancer cells from androgen deprivation therapy-induced senescence by mediating the SKP2/p27 axis. BMC Biol. (2025) 23:66. 10.1186/s12915-025-02172-4 40022149 PMC11871636

[42] Gong X Smith JR Swanson HM Rubin LP. Carotenoid lutein selectively inhibits breast cancer cell growth and potentiates the effect of chemotherapeutic agents through ROS-mediated mechanisms. Molecules. (2018) 23:905. 10.3390/molecules23040905 29662002 PMC6017803

[43] Andrysik Z Espinosa JM. Harnessing p53 for targeted cancer therapy: new advances and future directions. Transcription. (2025) 2025:1–44. 10.1080/21541264.2025.2452711 40031988 PMC11970777

[44] Tamura RE de Vasconcellos JF Sarkar D Libermann TA Fisher PB Zerbini LF. GADD45 proteins: central players in tumorigenesis. Curr Mol Med. (2012) 12:634–51. 10.2174/156652412800619978 22515981 PMC3797964

[45] Zhang WL Zhao YN Shi ZZ Cong D Bai YS. Lutein inhibits cell growth and activates apoptosis via the PI3K/AKT/mTOR signaling pathway in A549 human non-small-cell lung cancer cells. J Environ Pathol Toxicol Oncol. (2018) 37:341–50. 10.1615/JEnvironPatholToxicolOncol.2018027418 30806240

[46] Eom JW Lim JW Kim H. Lutein induces reactive oxygen species-mediated apoptosis in gastric cancer AGS cells via NADPH oxidase activation. Molecules. (2023) 28:1178. 10.3390/molecules28031178 36770846 PMC9919728

[47] Mirahmadi M Azimi-Hashemi S Saburi E Kamali H Pishbin M Hadizadeh F. Potential inhibitory effect of lycopene on prostate cancer. Biomed Pharmacother. (2020) 129:110459. 10.1016/j.biopha.2020.110459 32768949

[48] Zhang Y Yang J Na X Zhao A. Association between β-carotene supplementation and risk of cancer: a meta-analysis of randomized controlled trials. Nutr Rev. (2023) 81:1118–30. 10.1093/nutrit/nuac110 36715090

[49] Shin J Song MH Oh JW Keum YS Saini RK. Pro-oxidant actions of carotenoids in triggering apoptosis of cancer cells: a review of emerging evidence. Antioxidants. (2020) 9:532. 10.3390/antiox9060532 32560478 PMC7346220

[50] Tojjari A Choucair K Sadeghipour A Saeed A Saeed A. Anti-inflammatory and immune properties of polyunsaturated fatty acids (PUFAs) and their impact on colorectal cancer (CRC) prevention and treatment. Cancers. (2023) 15:4294. 10.3390/cancers15174294 37686570 PMC10487099

[51] Han L Zhang Y Meng M Cheng D Wang C. Eicosapentaenoic acid induced SKOV-3 cell apoptosis through ERK1/2-mTOR-NF-κB pathways. Anticancer Drugs. (2016) 27:635–42. 10.1097/CAD.0000000000000373 27176035

[52] Calviello G Serini S Piccioni E. n-3 polyunsaturated fatty acids and the prevention of colorectal cancer: molecular mechanisms involved. Curr Med Chem. (2007) 14:3059–69. 10.2174/092986707782793934 18220742

[53] Chiang N Serhan CN. Structural elucidation and physiologic functions of specialized pro-resolving mediators and their receptors. Mol Aspects Med. (2017) 58:114–29. 10.1016/j.mam.2017.03.005 28336292 PMC5623601

[54] Calviello G Resci F Serini S Piccioni E Toesca A Boninsegna A. Docosahexaenoic acid induces proteasome-dependent degradation of beta-catenin, down-regulation of survivin and apoptosis in human colorectal cancer cells not expressing COX-2. Carcinogenesis. (2007) 28:1202–9. 10.1093/carcin/bgl254 17183061

[55] Liu Z Luo Y Ren J Yang L Li J Wei Z. Association between fish oil supplementation and cancer risk according to fatty fish consumption: a large prospective population-based cohort study using UK Biobank. Int J Cancer. (2022) 150:562–71. 10.1002/ijc.33819 34558660

[56] Calviello G Palozza P Piccioni E Maggiano N Frattucci A Franceschelli P. Dietary supplementation with eicosapentaenoic and docosahexaenoic acid inhibits growth of Morris hepatocarcinoma 3924A in rats: effects on proliferation and apoptosis. Int J Cancer. (1998) 75:699–705. 9495237 10.1002/(sici)1097-0215(19980302)75:5<699::aid-ijc7>3.0.co;2-u

[57] Fuentes NR Kim E Fan YY Chapkin RS. Omega-3 fatty acids, membrane remodeling and cancer prevention. Mol Aspects Med. (2018) 64:79–91. 10.1016/j.mam.2018.04.001 29627343 PMC6185832

[58] Turk HF Barhoumi R Chapkin RS. Alteration of EGFR spatiotemporal dynamics suppresses signal transduction. PLoS ONE. (2012) 7:e39682. 10.1371/journal.pone.0039682 22761867 PMC3384615

[59] Slaninova V Krafcikova M Perez-Gomez R Steffal P Trantirek L Bray SJ. Notch stimulates growth by direct regulation of genes involved in the control of glycolysis and the tricarboxylic acid cycle. Open Biol. (2016) 6:150155. 10.1098/rsob.150155 26887408 PMC4772804

[60] Montecillo-Aguado M Tirado-Rodriguez B Huerta-Yepez S. The involvement of polyunsaturated fatty acids in apoptosis mechanisms and their implications in cancer. Int J Mol Sci. (2023) 24:11691. 10.3390/ijms241411691 37511450 PMC10380946

[61] Oono K Ohtake K Watanabe C Shiba S Sekiya T Kasono K. Contribution of Pyk2 pathway and reactive oxygen species (ROS) to the anti-cancer effects of eicosapentaenoic acid (EPA) in PC3 prostate cancer cells. Lipids Health Dis. (2020) 19:15. 10.1186/s12944-019-1122-4 32005121 PMC6993438

[62] Dumont A de Rosny C Kieu TL Perrey S Berger H Fluckiger A. Docosahexaenoic acid inhibits both NLRP3 inflammasome assembly and JNK-mediated mature IL-1β secretion in 5-fluorouracil-treated MDSC: implication in cancer treatment. Cell Death Dis. (2019) 10:485. 10.1038/s41419-019-1723-x 31217433 PMC6584690

[63] Cheng M Zhang S Ning C Huo Q. Omega-3 fatty acids supplementation improve nutritional status and inflammatory response in patients with lung cancer: a randomized clinical trial. Front Nutr. (2021) 8:686752. 10.3389/fnut.2021.686752 34395492 PMC8362886

[64] LeMay-Nedjelski L Mason-Ennis JK Taibi A Comelli EM Thompson LU. Omega-3 polyunsaturated fatty acids time-dependently reduce cell viability and oncogenic microRNA-21 expression in estrogen receptor-positive breast cancer cells (MCF-7). Int J Mol Sci. (2018) 19:244. 10.3390/ijms19010244 29342901 PMC5796192

[65] Yetiv JZ. Clinical applications of fish oils. JAMA. (1988) 260:665–70. 10.1001/jama.1988.03410050085035 3292794

[66] Gorjao R Dos Santos CMM Serdan TDA Diniz VLS Alba-Loureiro TC Cury-Boaventura MF. New insights on the regulation of cancer cachexia by N-3 polyunsaturated fatty acids. Pharmacol Ther. (2019) 196:117–34. 10.1016/j.pharmthera.2018.12.001 30521881

[67] Clamon G Byrne MM Talbert EE. Inflammation as a therapeutic target in cancer cachexia. Cancers. (2022) 14:5262. 10.3390/cancers14215262 36358681 PMC9657920

[68] Therdyothin A Phiphopthatsanee N Isanejad M. The effect of omega-3 fatty acids on sarcopenia: mechanism of action and potential efficacy. Mar Drugs. (2023) 21:399. 10.3390/md21070399 37504930 PMC10381755

[69] Belury MA Cole RM Andridge R Keiter A Raman SV Lustberg MB. Erythrocyte long-chain ω-3 fatty acids are positively associated with lean mass and grip strength in women with recent diagnoses of breast cancer. J Nutr. (2021) 151:2125–33. 10.1093/jn/nxab109 34036350 PMC8349126

[70] Fernandes SA Angelidaki DD Nüchel J Pan J Gollwitzer P Elkis Y. Spatial and functional separation of mTORC1 signalling in response to different amino acid sources. Nat Cell Biol. (2024) 26:1918–33. 10.1038/s41556-024-01523-7 39385049 PMC11567901

[71] Cristofano MD Ferramosca A Giacomo MD Fusco C Boscaino F Luongo D. Mechanisms underlying the hormetic effect of conjugated linoleic acid: Focus on Nrf2, mitochondria and NADPH oxidases. Free Radic Biol Med. (2021) 167:276–86. 10.1016/j.freeradbiomed.2021.03.015 33753237

[72] Shahidi F Ambigaipalan P. Omega-3 polyunsaturated fatty acids and their health benefits. Annu Rev Food Sci Technol. (2018) 9:345–81. 10.1146/annurev-food-111317-095850 29350557

[73] Read JA Beale PJ Volker DH Smith N Childs A Clarke SJ. Nutrition intervention using an eicosapentaenoic acid (EPA)-containing supplement in patients with advanced colorectal cancer. Effects on nutritional and inflammatory status: a phase II trial. Support Care Cancer. (2007) 15:301–7. 10.1007/s00520-006-0153-3 17021855

[74] Ryan AM Reynolds JV Healy L Byrne M Moore J Brannelly N. Enteral nutrition enriched with eicosapentaenoic acid (EPA) preserves lean body mass following esophageal cancer surgery: results of a double-blinded randomized controlled trial. Ann Surg. (2009) 249:355–63. 10.1097/SLA.0b013e31819a4789 19247018

[75] Hosseini F Hemmati A Takabi FS Naeini F Shab Bidar S A. dose-response meta-analysis of randomized clinical trials investigating the effects of omega-3 supplementation on body weight in patients with cancer cachexia. Clin Nutr ESPEN. (2024) 59:378–86. 10.1016/j.clnesp.2023.12.150 38220400

[76] de Castro GS Andrade MF Pinto FCS Faiad JZ Seelaender M. Omega-3 fatty acid supplementation and its impact on systemic inflammation and body weight in patients with cancer cachexia-a systematic review and meta-analysis. Front Nutr. (2021) 8:797513. 10.3389/fnut.2021.797513 35174197 PMC8841833

[77] Solheim TS Laird BJA Balstad TR Stene GB Bye A Johns N. A randomized phase II feasibility trial of a multimodal intervention for the management of cachexia in lung and pancreatic cancer. J Cachexia Sarcopenia Muscle. (2017) 8:778–88. 10.1002/jcsm.12201 28614627 PMC5659068

[78] Ikeda I Tanaka K Sugano M Vahouny GV Gallo LL. Inhibition of cholesterol absorption in rats by plant sterols. J Lipid Res. (1988) 29:1573–82. 10.1016/S0022-2275(20)38403-0 2468730

[79] Awad AB Williams H Fink CS. Phytosterols reduce in vitro metastatic ability of MDA-MB-231 human breast cancer cells. Nutr Cancer. (2001) 40:157–64. 10.1207/S15327914NC402_12 11962251

[80] Wang H Wang Z Zhang Z Liu J Hong L. β-sitosterol as a promising anticancer agent for chemoprevention and chemotherapy: mechanisms of action and future prospects. Adv Nutr. (2023) 14:1085–110. 10.1016/j.advnut.2023.05.013 37247842 PMC10509430

[81] Hueber AO Bernard AM Herincs Z Couzinet A He HT. An essential role for membrane rafts in the initiation of Fas/CD95-triggered cell death in mouse thymocytes. EMBO Rep. (2002) 3:190–6. 10.1093/embo-reports/kvf022 11818332 PMC1083963

[82] Long J Zhang CJ Zhu N Du K Yin YF Tan X. Lipid metabolism and carcinogenesis, cancer development. Am J Cancer Res. (2018) 8:778–91. 29888102 PMC5992506

[83] Mukherjee S Banik SK Chakraborty S Das T Choudhury MD Tripathi A. Bryophyllum pinnatum Induces p53-dependent apoptosis of colorectal cancer cells via increased intracellular ROS and G2/M cell-cycle arrest in vitro and validated in silico by molecular docking. Cell Biol Int. (2025) 49:534–54. 10.1002/cbin.70004 39992739

[84] Rakhi SA Hara Y Islam MS Manome T Alam S Emon NU. Isolation of bioactive phytochemicals from *Crinum asiaticum* L. along with their cytotoxic and TRAIL-resistance abrogating prospect assessment. Heliyon. (2024) 10:e25049. 10.1016/j.heliyon.2024.e25049 38318065 PMC10838800

[85] Xi M Kia SH Shi H Dong X Shi Y Zhang L. Synthesis and characterization of berberine-loaded nanoliposome for targeting of MAPK pathway to induce apoptosis and suppression of autophagy in glioblastoma. Biomed Mater. (2025) 20:adb673. 10.1088/1748-605X/adb673 39951894

[86] Deng X Lei HY Ren YS Ai J Li YQ Liang S. A novel strategy for active compound efficacy status identification in multi-tropism Chinese herbal medicine (Scutellaria baicalensis Georgi) based on multi-indexes spectrum-effect gray correlation analysis. J Ethnopharmacol. (2023) 300:115677. 10.1016/j.jep.2022.115677 36064148

[87] Bashir R Ahmad Zargar O Hamid Dar A Yedukondalu N Parvaiz Q Hamid R. The modulation of PI3K/Akt pathway by 3β hydroxylup-12-en-28-oic acid isolated from Thymus linearis induces cell death in HCT-116 cells. Chem Biol Drug Des. (2022) 99:162–78. 10.1111/cbdd.13957 34558199

[88] Khaled AM Othman MS Obeidat ST Aleid GM Aboelnaga SM Fehaid A. Green-synthesized silver and selenium nanoparticles using berberine: a comparative assessment of in vitro anticancer potential on human hepatocellular carcinoma cell line (HepG2) cells. (2024) 13:287. 10.3390/cells13030287 38334679 PMC10854975

[89] Nazir MM Farzeen I Fasial S Ashraf A. Berberine in rheumatoid arthritis: a comprehensive review and meta-analysis of its anti-inflammatory and immunomodulatory mechanisms in animal models. Inflammopharmacology. (2025) 33:215–29. 10.1007/s10787-024-01612-x 39710763

[90] Tang X Yan T Wang S Liu Q Yang Q Zhang Y. Treatment with β-sitosterol ameliorates the effects of cerebral ischemia/reperfusion injury by suppressing cholesterol overload, endoplasmic reticulum stress, and apoptosis. Neural Regen Res. (2024) 19:642–9. 10.4103/1673-5374.380904 37721296 PMC10581587

[91] Wang G Cheng L Shao Z Li S Sun W Liu J. Treatment of delayed fracture healing with Bushen Tiansui decoction: Analysis of active agents and targets using bioinformatics and network pharmacology analysis. Int J Clin Pharmacol Ther. (2025) 63:114–38. 10.5414/CP204705 39810723

[92] Mathakala V Ullakula T Palempalli UMD. Seagrass as a potential nutraceutical to decrease pro-inflammatory markers. BMC Complement Med Ther. (2024) 24:260. 10.1186/s12906-024-04532-z 38987758 PMC11234661

[93] Sundstrøm T Prestegarden L Azuaje F Aasen SN Røsland GV Varughese JK. Inhibition of mitochondrial respiration prevents BRAF-mutant melanoma brain metastasis. Acta Neuropathol Commun. (2019) 7:55. 10.1186/s40478-019-0712-8 30971321 PMC6456988

[94] Kawano M Takagi R Kaneko A Matsushita S. Berberine is a dopamine D1- and D2-like receptor antagonist and ameliorates experimentally induced colitis by suppressing innate and adaptive immune responses. J Neuroimmunol. (2015) 289:43–55. 10.1016/j.jneuroim.2015.10.001 26616870

[95] Wang YX Pang WQ Zeng QX Deng ZS Fan TY Jiang JD. Synthesis and biological evaluation of new berberine derivatives as cancer immunotherapy agents through targeting IDO1. Eur J Med Chem. (2018) 143:1858–68. 10.1016/j.ejmech.2017.10.078 29133053

[96] Wang K Gu C Yu G Lin J Wang Z Lu Q. Berberine enhances the anti-hepatocellular carcinoma effect of NK92-MI cells through inhibiting IFN-gamma-mediated PD-L1 expression. Liver Res. (2022) 6:167–74. 10.1016/j.livres.2022.08.003 39958198 PMC11791859

[97] Barut Z Aslan M Çirçirli B Çeker T Yilmaz Ç. Antiproliferative effect of 7-ketositosterol in breast and liver cancer cells: possible impact on ceramide, extracellular signal-regulated kinases, and nuclear factor kappa b signaling pathways. Pharmaceuticals. (2024) 17:860. 10.3390/ph17070860 39065711 PMC11279788

[98] Hassan MS Awasthi N Ponna S von Holzen U. Nab-paclitaxel in the treatment of gastrointestinal cancers-improvements in clinical efficacy and safety. Biomedicines. (2023) 11:2000. 10.3390/biomedicines11072000 37509639 PMC10377238

[99] Alatise KL Gardner S Alexander-Bryant A. Mechanisms of drug resistance in ovarian cancer and associated gene targets. Cancers. (2022) 14:6246. 10.3390/cancers14246246 36551731 PMC9777152

[100] Tian Q Zhang P Wang Y Si Y Yin D Weber CR. A novel triptolide analog downregulates NF-κB and induces mitochondrial apoptosis pathways in human pancreatic cancer. Elife. (2023) 12:e85862. 10.7554/eLife.85862 37877568 PMC10861173

[101] Wang Y Liu Y Du X Ma H Yao J. The anti-cancer mechanisms of berberine: a review. Cancer Manag Res. (2020) 12:695–702. 10.2147/CMAR.S242329 32099466 PMC6996556

[102] Zheng L Chen J Ma Z Liu W Yang F Yang Z. Capsaicin causes inactivation and degradation of the androgen receptor by inducing the restoration of miR-449a in prostate cancer. Oncol Rep. (2015) 34:1027–34. 10.3892/or.2015.4055 26081756

[103] Mitra S Anand U Jha NK Shekhawat MS Saha SC Nongdam P. Anticancer applications and pharmacological properties of piperidine and piperine: a comprehensive review on molecular mechanisms and therapeutic perspectives. Front Pharmacol. (2021) 12:772418. 10.3389/fphar.2021.772418 35069196 PMC8776707

[104] Cui WQ Wang ST Pan D Chang B Sang LX. Caffeine and its main targets of colorectal cancer. World J Gastrointest Oncol. (2020) 12:149–72. 10.4251/wjgo.v12.i2.149 32104547 PMC7031145

[105] Romualdo GR Prata GB da Silva TC Evangelista AF Reis RM Vinken M. The combination of coffee compounds attenuates early fibrosis-associated hepatocarcinogenesis in mice: involvement of miRNA profile modulation. J Nutr Biochem. (2020) 85:108479. 10.1016/j.jnutbio.2020.108479 32795656

[106] Aune D Giovannucci E Boffetta P Fadnes LT Keum N Norat T. Fruit and vegetable intake and the risk of cardiovascular disease, total cancer and all-cause mortality-a systematic review and dose-response meta-analysis of prospective studies. Int J Epidemiol. (2017) 46:1029–56. 10.1093/ije/dyw319 28338764 PMC5837313

[107] Rakariyatham K Yang X Gao Z Song M Han Y Chen X. Synergistic chemopreventive effect of allyl isothiocyanate and sulforaphane on non-small cell lung carcinoma cells. Food Funct. (2019) 10:893–902. 10.1039/C8FO01914B 30694275 PMC6611553

[108] Arumugam A Abdull Razis AF. Apoptosis as a Mechanism of the Cancer Chemopreventive Activity of Glucosinolates: a Review. Asian Pac J Cancer Prev. (2018) 19:1439–48. 10.22034/APJCP.2018.19.6.1439 29936713 PMC6103590

[109] Yu R Mandlekar S Harvey KJ Ucker DS Kong AN. Chemopreventive isothiocyanates induce apoptosis and caspase-3-like protease activity. Cancer Res. (1998) 58:402–8. 9458080

[110] Sehrawat A Croix CS Baty CJ Watkins S Tailor D Singh RP. Inhibition of mitochondrial fusion is an early and critical event in breast cancer cell apoptosis by dietary chemopreventative benzyl isothiocyanate. Mitochondrion. (2016) 30:67–77. 10.1016/j.mito.2016.06.006 27374852 PMC5023488

[111] Shoaib S Tufail S Sherwani MA Yusuf N Islam N. Phenethyl isothiocyanate induces apoptosis through ros generation and caspase-3 activation in cervical cancer cells. Front Pharmacol. (2021) 12:673103. 10.3389/fphar.2021.673103 34393773 PMC8358204

[112] Dong X Yu X Lu M Xu Y Zhou L Peng T. Quantitative chemical proteomics reveals that phenethyl isothiocyanate covalently targets BID to promote apoptosis. Cell Death Discov. (2024) 10:456. 10.1038/s41420-024-02225-7 39472556 PMC11522290

[113] Na G He C Zhang S Tian S Bao Y Shan Y. Dietary isothiocyanates: novel insights into the potential for cancer prevention and therapy. Int J Mol Sci. (2023) 24:1962. 10.3390/ijms24031962 36768284 PMC9916827

[114] Wang Q Li D Liu L Shan Y Bao Y. Dietary isothiocyanates and anticancer agents: exploring synergism for improved cancer management. Front Nutr. (2024) 11:1386083. 10.3389/fnut.2024.1386083 38919393 PMC11196812

[115] Vanduchova A Anzenbacher P Anzenbacherova E. Isothiocyanate from broccoli, sulforaphane, and its properties. J Med Food. (2019) 22:121–6. 10.1089/jmf.2018.0024 30372361

[116] Liu P Zhang B Li Y Yuan Q. Potential mechanisms of cancer prevention and treatment by sulforaphane, a natural small molecule compound of plant-derived. Mol Med. (2024) 30:94. 10.1186/s10020-024-00842-7 38902597 PMC11191161

[117] Somers DJ Kushner DB McKinnis AR Mehmedovic D Flame RS Arnold TM. Epigenetic weapons in plant-herbivore interactions: Sulforaphane disrupts histone deacetylases, gene expression, and larval development in Spodoptera exigua while the specialist feeder Trichoplusia ni is largely resistant to these effects. PLoS ONE. (2023) 18:e0293075. 10.1371/journal.pone.0293075 37856454 PMC10586618

[118] Sailo BL Liu L Chauhan S Girisa S Hegde M Liang L. Harnessing sulforaphane potential as a chemosensitizing agent: a comprehensive review. Cancers. (2024) 16:244. 10.3390/cancers16020244 38254735 PMC10814109

[119] Lan F Yang Y Han J Wu Q Yu H Yue X. Sulforaphane reverses chemo-resistance to temozolomide in glioblastoma cells by NF-κB-dependent pathway downregulating MGMT expression. Int J Oncol. (2016) 48:559–68. 10.3892/ijo.2015.3271 26648123

[120] Asif Ali M Khan N Kaleem N Ahmad W Alharethi SH Alharbi B. Anticancer properties of sulforaphane: current insights at the molecular level. Front Oncol. (2023) 13:1168321. 10.3389/fonc.2023.1168321 37397365 PMC10313060

[121] Coutinho LL Junior TCT Rangel MC. Sulforaphane: An emergent anti-cancer stem cell agent. Front Oncol. (2023) 13:1089115. 10.3389/fonc.2023.1089115 36776295 PMC9909961

[122] Abbaoui B Lucas CR Riedl KM Clinton SK Mortazavi A. Cruciferous vegetables, isothiocyanates, and bladder cancer prevention. Mol Nutr Food Res. (2018) 62:e1800079. 10.1002/mnfr.201800079 30079608 PMC6196731

[123] Wang M Tang L Chen S Wang L Wu J Zhong C. ZNF217-activated Notch signaling mediates sulforaphane-suppressed stem cell properties in colorectal cancer. J Nutr Biochem. (2024) 125:109551. 10.1016/j.jnutbio.2023.109551 38134973

[124] Zhang F Wan X Zhan J Shen M Li R. Sulforaphane inhibits the growth of prostate cancer by regulating the microRNA-3919/DJ-1 axis. Front Oncol. (2024) 14:1361152. 10.3389/fonc.2024.1361152 38515566 PMC10955061

[125] Li X Zhao Z Li M Liu M Bahena A Zhang Y. Sulforaphane promotes apoptosis, and inhibits proliferation and self-renewal of nasopharyngeal cancer cells by targeting STAT signal through miRNA-124-3p. Biomed Pharmacother. (2018) 103:473–81. 10.1016/j.biopha.2018.03.121 29677532

[126] Zhang C Shu L Kim H Khor TO Wu R Li W. Phenethyl isothiocyanate (PEITC) suppresses prostate cancer cell invasion epigenetically through regulating microRNA-194. Mol Nutr Food Res. (2016) 60:1427–36. 10.1002/mnfr.201500918 26820911 PMC5495185

[127] Lin JF Tsai TF Lin YC Chen HE Chou KY Hwang TI. Benzyl isothiocyanate suppresses IGF1R, FGFR3 and mTOR expression by upregulation of miR-99a-5p in human bladder cancer cells. Int J Oncol. (2019) 54:2106–16. 10.3892/ijo.2019.4763 30942430

[128] Powolny AA Bommareddy A Hahm ER Normolle DP Beumer JH Nelson JB. Chemopreventative potential of the cruciferous vegetable constituent phenethyl isothiocyanate in a mouse model of prostate cancer. J Natl Cancer Inst. (2011) 103:571–84. 10.1093/jnci/djr029 21330634 PMC3071352

[129] Yuan JM Kensler TW Dacic S Hartman DJ Wang R Balogh PA. Randomized phase II clinical trial of sulforaphane in former smokers at high risk for lung cancer. Cancer Prev Res (Phila). (2025) 18:335–45. 10.1158/1940-6207.CAPR-24-0386 40041932 PMC13321263

[130] Alhakamy NA Saquib M Sanobar Khan MF Ansari WA Arif DO Irfan M. Natural product-inspired synthesis of coumarin-chalcone hybrids as potential anti-breast cancer agents. Front Pharmacol. (2023) 14:1231450. 10.3389/fphar.2023.1231450 37745072 PMC10511752

[131] Lu J He R Sun P Zhang F Linhardt RJ Zhang A. Molecular mechanisms of bioactive polysaccharides from Ganoderma lucidum (Lingzhi), a review. Int J Biol Macromol. (2020) 150:765–74. 10.1016/j.ijbiomac.2020.02.035 32035956

[132] Feng YY Ji HY Dong XD Liu AJ. An alcohol-soluble polysaccharide from Atractylodes macrocephala Koidz induces apoptosis of Eca-109 cells. Carbohydr Polym. (2019) 226:115136. 10.1016/j.carbpol.2019.115136 31582084

[133] Jin JO Yadav D Madhwani K Puranik N Chavda V Song M. Seaweeds in the oncology arena: anti-cancer potential of fucoidan as a drug-a review. Molecules. (2022) 27:6032. 10.3390/molecules27186032 36144768 PMC9506145

[134] Meng X Liang H Luo L. Antitumor polysaccharides from mushrooms: a review on the structural characteristics, antitumor mechanisms and immunomodulating activities. Carbohydr Res. (2016) 424:30–41. 10.1016/j.carres.2016.02.008 26974354

[135] Wang A Liu Y Zeng S Liu Y Li W Wu D. Dietary plant polysaccharides for cancer prevention: role of immune cells and gut microbiota, challenges and perspectives. Nutrients. (2023) 15:3019. 10.3390/nu15133019 37447345 PMC10347129

[136] Huang G Huang H. The derivatization and antitumor mechanisms of polysaccharides. Future Med Chem. (2017) 9:1931–8. 10.4155/fmc-2017-0132 29076350

[137] Lu X Qin L Guo M Geng J Dong S Wang K. A novel alginate from Sargassum seaweed promotes diabetic wound healing by regulating oxidative stress and angiogenesis. Carbohydr Polym. (2022) 289:119437. 10.1016/j.carbpol.2022.119437 35483850

[138] Ren F Wu K Yang Y Yang Y Wang Y Li J. Dandelion polysaccharide exerts anti-angiogenesis effect on hepatocellular carcinoma by regulating VEGF/HIF-1α expression. Front Pharmacol. (2020) 11:460. 10.3389/fphar.2020.00460 32322211 PMC7158757

[139] Jiao R Liu Y Gao H Xiao J So KF. The anti-oxidant and antitumor properties of plant polysaccharides. Am J Chin Med. (2016) 44:463–88. 10.1142/S0192415X16500269 27109156

[140] Li C Hu Y Yang H. Plant polysaccharides influence tumor development based on epigenetics: a review. Front Pharmacol. (2025) 16:1588857. 10.3389/fphar.2025.1588857 40395727 PMC12089089

[141] Steimbach L Borgmann AV Gomar GG Hoffmann LV Rutckeviski R de Andrade DP. Fungal beta-glucans as adjuvants for treating cancer patients - A systematic review of clinical trials. Clin Nutr. (2021) 40:3104–13. 10.1016/j.clnu.2020.11.029 33309412

[142] Hui MK Wu WK Shin VY So WH Cho CH. Polysaccharides from the root of Angelica sinensis protect bone marrow and gastrointestinal tissues against the cytotoxicity of cyclophosphamide in mice. Int J Med Sci. (2006) 3:1–6. 10.7150/ijms.3.1 16421623 PMC1332197

[143] Shen M Wang YJ Liu ZH Chen YW Liang QK Li Y. Inhibitory effect of astragalus polysaccharide on premetastatic niche of lung cancer through the S1PR1-STAT3 signaling pathway. Evid Based Complement Alternat Med. (2023) 2023:4010797. 10.1155/2023/4010797 36714534 PMC9883101

[144] Wu SY Wu AT Yuan KS Liu SH. Brown seaweed fucoidan inhibits cancer progression by dual regulation of mir-29c/ADAM12 and miR-17-5p/PTEN axes in human breast cancer cells. J Cancer. (2016) 7:2408–19. 10.7150/jca.15703 27994679 PMC5166552

[145] Tao X Zhang X Feng F. Astragalus polysaccharide suppresses cell proliferation and invasion by up-regulation of miR-195-5p in non-small cell lung cancer. Biol Pharm Bull. (2022) 45:553–60. 10.1248/bpb.b21-00634 35315366

[146] Yang Q Meng D Zhang Q Wang J. Advances in research on the anti-tumor mechanism of Astragalus polysaccharides. Front Oncol. (2024) 14:1334915. 10.3389/fonc.2024.1334915 38515577 PMC10955345

[147] Abdulmalek SA Saleh AM Shahin YR El Azab EF. Functionalized siRNA-chitosan nanoformulations promote triple-negative breast cancer cell death via blocking the miRNA-21/AKT/ERK signaling axis: in-silico and in vitro studies. Naunyn Schmiedebergs Arch Pharmacol. (2024) 397:6941–62. 10.1007/s00210-024-03068-w 38592437 PMC11422444

[148] Bakrim S El Omari N El Hachlafi N Bakri Y Lee LH Bouyahya A. Dietary phenolic compounds as anticancer natural drugs: recent update on molecular mechanisms and clinical trials. Foods. (2022) 11:3323. 10.3390/foods11213323 36359936 PMC9657352

[149] Agarwal C Tyagi A Agarwal R. Gallic acid causes inactivating phosphorylation of cdc25A/cdc25C-cdc2 via ATM-Chk2 activation, leading to cell cycle arrest, and induces apoptosis in human prostate carcinoma DU145 cells. Mol Cancer Ther. (2006) 5:3294–302. 10.1158/1535-7163.MCT-06-0483 17172433

[150] Pavlíková N. Caffeic acid and diseases-mechanisms of action. Int J Mol Sci. (2022) 24:588. 10.3390/ijms24010588 36614030 PMC9820408

[151] Ko EB Jang YG Kim CW Go RE Lee HK Choi KC. Gallic acid hindered lung cancer progression by inducing cell cycle arrest and apoptosis in A549 lung cancer cells via PI3K/Akt pathway. Biomol Ther. (2022) 30:151–61. 10.4062/biomolther.2021.074 34261818 PMC8902450

[152] Alam M Ahmed S Elasbali AM Adnan M Alam S Hassan MI. Therapeutic implications of caffeic acid in cancer and neurological diseases. Front Oncol. (2022) 12:860508. 10.3389/fonc.2022.860508 35359383 PMC8960963

[153] Rajendran P Abdelsalam SA Renu K Veeraraghavan V Ben Ammar R Ahmed EA. Polyphenols as potent epigenetics agents for cancer. Int J Mol Sci. (2022) 23:1712. 10.3390/ijms231911712 36233012 PMC9570183

[154] Sharifi-Rad J Seidel V Izabela M Monserrat-Mequida M Sureda A Ormazabal V. Phenolic compounds as Nrf2 inhibitors: potential applications in cancer therapy. Cell Commun Signal. (2023) 21:89. 10.1186/s12964-023-01109-0 37127651 PMC10152593

[155] Sanchez-Martin V Plaza-Calonge MDC Soriano-Lerma A Ortiz-Gonzalez M Linde-Rodriguez A Perez-Carrasco V. Gallic acid: a natural phenolic compound exerting antitumoral activities in colorectal cancer via interaction with G-quadruplexes. Cancers. (2022) 14:2648. 10.3390/cancers14112648 35681628 PMC9179882

[156] Qanash H Yahya R Bakri MM Bazaid AS Qanash S Shater AF. Anticancer, antioxidant, antiviral and antimicrobial activities of Kei Apple (*Dovyalis caffra*) fruit. Sci Rep. (2022) 12:5914. 10.1038/s41598-022-09993-1 35396383 PMC8990652

[157] Sweed NM Dawoud MHS Aborehab NM Ezzat SM. An approach for an enhanced anticancer activity of ferulic acid-loaded polymeric micelles via MicroRNA-221 mediated activation of TP53INP1 in caco-2 cell line. Sci Rep. (2024) 14:2073. 10.1038/s41598-024-52143-y 38267567 PMC10808409

[158] You Z Lei Y Yang Y Zhou Z Chao X Ju K. Therapeutic target genes and regulatory networks of gallic acid in cervical cancer. Front Genet. (2024) 15:1508869. 10.3389/fgene.2024.1508869 39902297 PMC11789760

[159] Huang CC Tsai MC Wu YL Lee YJ Yen AT Wang CJ. Gallic acid attenuates metastatic potential of human colorectal cancer cells through the miR-1247-3p-modulated integrin/FAK axis. Environ Toxicol. (2024) 39:2077–85. 10.1002/tox.24087 38100242

[160] Huang S Wang LL Xue NN Li C Guo HH Ren TK. Chlorogenic acid effectively treats cancers through induction of cancer cell differentiation. Theranostics. (2019) 9:6745–63. 10.7150/thno.34674 31660066 PMC6815948

[161] Patra S Bhol CS Panigrahi DP Praharaj PP Pradhan B Jena M. Gamma irradiation promotes chemo-sensitization potential of gallic acid through attenuation of autophagic flux to trigger apoptosis in an NRF2 inactivation signalling pathway. Free Radic Biol Med. (2020) 160:111–24. 10.1016/j.freeradbiomed.2020.06.022 32755671

[162] Min J Shen H Xi W Wang Q Yin L Zhang Y. Synergistic anticancer activity of combined use of caffeic acid with paclitaxel enhances apoptosis of non-small-cell lung cancer H1299 cells *in vivo* and *in vitro*. Cell Physiol Biochem. (2018) 48:1433–42. 10.1159/000492253 30064123

[163] Wendlocha D Kubina R Krzykawski K Mielczarek-Palacz A. Selected flavonols targeting cell death pathways in cancer therapy: the latest achievements in research on apoptosis, autophagy, necroptosis, pyroptosis, ferroptosis, and cuproptosis. Nutrients. (2024) 16:1201. 10.3390/nu16081201 38674891 PMC11053927

[164] Silva-Pinto PA de Pontes JTC Aguilar-Morón B Canales CSC Pavan FR Roque-Borda CA. Phytochemical insights into flavonoids in cancer: Mechanisms, therapeutic potential, and the case of quercetin. Heliyon. (2025) 11:e42682. 10.1016/j.heliyon.2025.e42682 40084006 PMC11904581

[165] Islam A Su AJ Zeng ZM Chueh PJ Lin MH. Capsaicin targets tNOX (ENOX2) to inhibit G1 Cyclin/CDK complex, as assessed by the cellular thermal shift assay (CETSA). Cells. (2019) 8:1275. 10.3390/cells8101275 31635402 PMC6830080

[166] Venier NA Yamamoto T Sugar LM Adomat H Fleshner NE Klotz LH. Capsaicin reduces the metastatic burden in the transgenic adenocarcinoma of the mouse prostate model. Prostate. (2015) 75:1300–11. 10.1002/pros.23013 26047020

[167] Lee GR Jang SH Kim CJ Kim AR Yoon DJ Park NH. Capsaicin suppresses the migration of cholangiocarcinoma cells by down-regulating matrix metalloproteinase-9 expression via the AMPK-NF-κB signaling pathway. Clin Exp Metastasis. (2014) 31:897–907. 10.1007/s10585-014-9678-x 25217963

[168] Guan H Zhang W Liu H Jiang Y Li F Wu M. Quercetin induces apoptosis in HepG2 cells via directly interacting with YY1 to disrupt YY1-p53 interaction. Metabolites. (2023) 13:229. 10.3390/metabo13020229 36837850 PMC9968089

[169] Asgharian P Tazekand AP Hosseini K Forouhandeh H Ghasemnejad T Ranjbar M. Potential mechanisms of quercetin in cancer prevention: focus on cellular and molecular targets. Cancer Cell Int. (2022) 22:257. 10.1186/s12935-022-02677-w 35971151 PMC9380290

[170] Kubina R Krzykawski K Dziedzic A Kabała-Dzik A. Kaempferol and fisetin-related signaling pathways induce apoptosis in head and neck cancer cells. Cells. (2023) 12:1568. 10.3390/cells12121568 37371038 PMC10297294

[171] Maggioni D Nicolini G Rigolio R Biffi L Pignataro L Gaini R. Myricetin and naringenin inhibit human squamous cell carcinoma proliferation and migration *in vitro*. Nutr Cancer. (2014) 66:1257–67. 10.1080/01635581.2014.951732 25256786

[172] Adetunji TL Olawale F Olisah C Adetunji AE Aremu AO. Capsaicin: a two-decade systematic review of global research output and recent advances against human cancer. Front Oncol. (2022) 12:908487. 10.3389/fonc.2022.908487 35912207 PMC9326111

[173] Amantini C Mosca M Nabissi M Lucciarini R Caprodossi S Arcella A. Capsaicin-induced apoptosis of glioma cells is mediated by TRPV1 vanilloid receptor and requires p38 MAPK activation. J Neurochem. (2007) 102:977–90. 10.1111/j.1471-4159.2007.04582.x 17442041

[174] Kida R Noguchi T Murakami M Hashimoto O Kawada T Matsui T. Supra-pharmacological concentration of capsaicin stimulates brown adipogenesis through induction of endoplasmic reticulum stress. Sci Rep. (2018) 8:845. 10.1038/s41598-018-19223-2 29339762 PMC5770457

[175] Zhang R Humphreys I Sahu RP Shi Y Srivastava SK. In vitro and in vivo induction of apoptosis by capsaicin in pancreatic cancer cells is mediated through ROS generation and mitochondrial death pathway. Apoptosis. (2008) 13:1465–78. 10.1007/s10495-008-0278-6 19002586

[176] Wong RS. Apoptosis in cancer: from pathogenesis to treatment. J Exp Clin Cancer Res. (2011) 30:87. 10.1186/1756-9966-30-87 21943236 PMC3197541

[177] Lin MH Lee YH Cheng HL Chen HY Jhuang FH Chueh PJ. Capsaicin inhibits multiple bladder cancer cell phenotypes by inhibiting tumor-associated NADH oxidase (tNOX) and Sirtuin1 (SIRT1). Molecules. (2016) 21:849. 10.3390/molecules21070849 27367652 PMC6272932

[178] Lavorgna M Orlo E Nugnes R Piscitelli C Russo C Isidori M. Capsaicin in hot chili peppers: *in vitro* evaluation of its antiradical, antiproliferative and apoptotic activities. Plant Foods Hum Nutr. (2019) 74:164–70. 10.1007/s11130-019-00722-0 30835044

[179] Joung EJ Li MH Lee HG Somparn N Jung YS Na HK. Capsaicin induces heme oxygenase-1 expression in HepG2 cells via activation of PI3K-Nrf2 signaling: NAD(P)H:quinone oxidoreductase as a potential target. Antioxid Redox Signal. (2007) 9:2087–98. 10.1089/ars.2007.1827 17979524

[180] Ahmad A Sakr WA Rahman KM. Anticancer properties of indole compounds: mechanism of apoptosis induction and role in chemotherapy. Curr Drug Targets. (2010) 11:652–66. 10.2174/138945010791170923 20298156

[181] Saha K Sarkar D Khan U Karmakar BC Paul S Mukhopadhyay AK. Capsaicin inhibits inflammation and gastric damage during *H pylori* Infection by targeting NF-kB-miRNA axis. Pathogens. (2022) 11:641. 10.3390/pathogens11060641 35745495 PMC9227394

[182] Bhutani M Pathak AK Nair AS Kunnumakkara AB Guha S Sethi G. Capsaicin is a novel blocker of constitutive and interleukin-6-inducible STAT3 activation. Clin Cancer Res. (2007) 13:3024–32. 10.1158/1078-0432.CCR-06-2575 17505005

[183] Zhang MQ Jia X Cheng CQ Wang YX Li YY Kong LD. Capsaicin functions as a selective degrader of STAT3 to enhance host resistance to viral infection. Acta Pharmacol Sin. (2023) 44:2253–64. 10.1038/s41401-023-01111-9 37311796 PMC10618195

[184] Talib WH AlHur MJ Al Naimat S Ahmad RE Al-Yasari AH Al-Dalaeen A. Anticancer effect of spices used in mediterranean diet: preventive and therapeutic potentials. Front Nutr. (2022) 9:905658. 10.3389/fnut.2022.905658 35774546 PMC9237507

[185] Chakraborty S Adhikary A Mazumdar M Mukherjee S Bhattacharjee P Guha D. Capsaicin-induced activation of p53-SMAR1 auto-regulatory loop down-regulates VEGF in non-small cell lung cancer to restrain angiogenesis. PLoS ONE. (2014) 9:e99743. 10.1371/journal.pone.0099743 24926985 PMC4057320

[186] Hwang YP Yun HJ Choi JH Han EH Kim HG Song GY. Suppression of EGF-induced tumor cell migration and matrix metalloproteinase-9 expression by capsaicin via the inhibition of EGFR-mediated FAK/Akt, PKC/Raf/ERK, p38 MAPK, and AP-1 signaling. Mol Nutr Food Res. (2011) 55:594–605. 10.1002/mnfr.201000292 21462327

[187] Xu S Zhang L Cheng X Yu H Bao J Lu R. Capsaicin inhibits the metastasis of human papillary thyroid carcinoma BCPAP cells through the modulation of the TRPV1 channel. Food Funct. (2018) 9:344–54. 10.1039/C7FO01295K 29185571

[188] El-Shehawy AA Elmetwalli A El-Far AH Mosallam SAE Salama AF Babalghith AO. Thymoquinone, piperine, and sorafenib combinations attenuate liver and breast cancers progression: epigenetic and molecular docking approaches. BMC Complement Med Ther. (2023) 23:69. 10.1186/s12906-023-03872-6 36870998 PMC9985300

[189] de Almeida GC Oliveira LFS Predes D Fokoue HH Kuster RM Oliveira FL. Piperine suppresses the Wnt/β-catenin pathway and has anti-cancer effects on colorectal cancer cells. Sci Rep. (2020) 10:11681. 10.1038/s41598-020-68574-2 32669593 PMC7363889

[190] Parama D Rana V Girisa S Verma E Daimary UD Thakur KK. The promising potential of piperlongumine as an emerging therapeutics for cancer. Explor Target Antitumor Ther. (2021) 2:323–54. 10.37349/etat.2021.00049 36046754 PMC9400693

[191] Lu X Xu C Xu Z Lu C Yang R Zhang F. Piperlongumine inhibits the growth of non-small cell lung cancer cells via the miR-34b-3p/TGFBR1 pathway. BMC Complement Med Ther. (2021) 21:15. 10.1186/s12906-020-03123-y 33413277 PMC7791704

[192] Farhan M. Insights on the role of polyphenols in combating cancer drug resistance. Biomedicines. (2023) 11:1709. 10.3390/biomedicines11061709 37371804 PMC10296548

[193] Eom DW Lee JH Kim YJ Hwang GS Kim SN Kwak JH. Synergistic effect of curcumin on epigallocatechin gallate-induced anticancer action in PC3 prostate cancer cells. BMB Rep. (2015) 48:461–6. 10.5483/BMBRep.2015.48.8.216 25441423 PMC4576954

[194] Theinel MH Nucci MP Alves AH Dias OFM Mamani JB Garrigós MM. The effects of omega-3 polyunsaturated fatty acids on breast cancer as a preventive measure or as an adjunct to conventional treatments. Nutrients. (2023) 15:1310. 10.3390/nu15061310 36986040 PMC10052714

[195] Sakhi AK Russnes KM Thoresen M Bastani NE Karlsen A Smeland S. Pre-radiotherapy plasma carotenoids and markers of oxidative stress are associated with survival in head and neck squamous cell carcinoma patients: a prospective study. BMC Cancer. (2009) 9:458. 10.1186/1471-2407-9-458 20025747 PMC2813240

[196] Meyer F Bairati I Jobin E Gélinas M Fortin A Nabid A. Acute adverse effects of radiation therapy and local recurrence in relation to dietary and plasma beta carotene and alpha tocopherol in head and neck cancer patients. Nutr Cancer. (2007) 59:29–35. 10.1080/01635580701397590 17927499

[197] Zhu W Mei H Jia L Zhao H Li X Meng X. Epigallocatechin-3-gallate mouthwash protects mucosa from radiation-induced mucositis in head and neck cancer patients: a prospective, non-randomised, phase 1 trial. Invest New Drugs. (2020) 38:1129–36. 10.1007/s10637-019-00871-8 31701429

[198] Schwingshackl L Schwedhelm C Galbete C Hoffmann G. An updated systematic review and meta-analysis on adherence to mediterranean diet and risk of cancer. Eur J Nutr. (2021) 60:1561–86. 10.1007/s00394-020-02346-6 32770356 PMC7987633

[199] Chen G Leary S Niu J Perry R Papadaki A. The role of the mediterranean diet in breast cancer survivorship: a systematic review and meta-analysis of observational studies and randomised controlled trials. Nutrients. (2023) 15:2099. 10.3390/nu15092099 37432242 PMC10180628

[200] Chlebowski RT Blackburn GL Thomson CA Nixon DW Shapiro A Hoy MK. Dietary fat reduction and breast cancer outcome: interim efficacy results from the Women's Intervention Nutrition Study. J Natl Cancer Inst. (2006) 98:1767−76. 10.1093/jnci/djj494 17179478

[201] Pierce JP Natarajan L Caan BJ Parker BA Greenberg ER Flatt SW. Influence of a diet very high in vegetables, fruit, and fiber and low in fat on prognosis following treatment for breast cancer: the Women's Healthy Eating and Living (WHEL) randomized trial. JAMA. (2007) 298:289–98. 10.1001/jama.298.3.289 17635889 PMC2083253

[202] Castro-Espin C Agudo A. The role of diet in prognosis among cancer survivors: a systematic review and meta-analysis of dietary patterns and diet interventions. Nutrients. (2022) 14:348. 10.3390/nu14020348 35057525 PMC8779048

[203] Bruno E Oliverio A Paradiso AV Daniele A Tommasi S Tufaro A. A mediterranean dietary intervention in female carriers of BRCA mutations: results from an italian prospective randomized controlled trial. Cancers. (2020) 12:3732. 10.3390/cancers12123732 33322597 PMC7764681

[204] Nouh WE El Azab EF Oraby EA Ahmed SM El-Eshmawy MA Badawy HK. Genetic variants and breast carcinoma susceptibility: unveiling the role of MTHFR (rs1801131, rs1801133) and TP53 (rs1042522) *Gene*. (2025) 942:149259. 10.1016/j.gene.2025.149259 39837367

[205] Vidmar Golja M Šmid A Karas Kuželički N Trontelj J Geršak K Mlinarič-Raščan I. Folate insufficiency due to MTHFR deficiency is bypassed by 5-Methyltetrahydrofolate. J Clin Med. (2020) 9:2836. 10.3390/jcm9092836 32887268 PMC7564482

[206] Peng J Wu Z MTHFR. act as a potential cancer biomarker in immune checkpoints blockades, heterogeneity, tumor microenvironment and immune infiltration. Discov Oncol. (2023) 14:112. 10.1007/s12672-023-00716-0 37354330 PMC10290629

[207] Lee KA Luong MK Shaw H Nathan P Bataille V Spector TD. The gut microbiome: what the oncologist ought to know. Br J Cancer. (2021) 125:1197–209. 10.1038/s41416-021-01467-x 34262150 PMC8548300

[208] Gunjur A Shao Y Rozday T Klein O Mu A Haak BW. A gut microbial signature for combination immune checkpoint blockade across cancer types. Nat Med. (2024) 30:797–809. 10.1038/s41591-024-02823-z 38429524 PMC10957475

[209] Teixeira M Silva F Ferreira RM Pereira T Figueiredo C Oliveira HP. A review of machine learning methods for cancer characterization from microbiome data. NPJ Precis Oncol. (2024) 8:123. 10.1038/s41698-024-00617-7 38816569 PMC11139966

[210] Spencer CN McQuade JL Gopalakrishnan V McCulloch JA Vetizou M Cogdill AP. Dietary fiber and probiotics influence the gut microbiome and melanoma immunotherapy response. Science. (2021) 374:1632–40. 10.1126/science.aaz7015 34941392 PMC8970537

[211] Valdés-Mas R Leshem A Zheng D Cohen Y Kern L Zmora N. Metagenome-informed metaproteomics of the human gut microbiome, host, and dietary exposome uncovers signatures of health and inflammatory bowel disease. Cell. (2025) 188:1062–83. 10.1016/j.cell.2024.12.016 39837331

[212] Arnone AA Ansley K Heeke AL Howard-McNatt M Cook KL. Gut microbiota interact with breast cancer therapeutics to modulate efficacy. EMBO Mol Med. (2025) 17:219–34. 10.1038/s44321-024-00185-0 39820166 PMC11822015

[213] Wu X Oniani D Shao Z Arciero P Sivarajkumar S Hilsman J. A scoping review of artificial intelligence for precision nutrition. Adv Nutr. (2025) 16:100398. 10.1016/j.advnut.2025.100398 40024275 PMC11994916

[214] Fountzilas E Pearce T Baysal MA Chakraborty A Tsimberidou AM. Convergence of evolving artificial intelligence and machine learning techniques in precision oncology. NPJ Digit Med. (2025) 8:75. 10.1038/s41746-025-01471-y 39890986 PMC11785769

[215] Muller E Shiryan I Borenstein E. Multi-omic integration of microbiome data for identifying disease-associated modules. Nat Commun. (2024) 15:2621. 10.1038/s41467-024-46888-3 38521774 PMC10960825

[216] Zitvogel L Derosa L Routy B Loibl S Heinzerling L de Vries IJM. Impact of the ONCOBIOME network in cancer microbiome research. Nat Med. (2025) 31:1085–98. 10.1038/s41591-025-03608-8 40217075

[217] Figueiredo JC Hsu L Hutter CM Lin Y Campbell PT Baron JA. Genome-wide diet-gene interaction analyses for risk of colorectal cancer. PLoS Genet. (2014) 10:e1004228. 10.1371/journal.pgen.1004228 24743840 PMC3990510

[218] Simon MC Sina C Ferrario PG Daniel H. Gut microbiome analysis for personalized nutrition: the state of science. Mol Nutr Food Res. (2023) 67:e2200476. 10.1002/mnfr.202200476 36424179

[219] Luca SV Macovei I Bujor A Miron A Skalicka-Wozniak K Aprotosoaie AC. Bioactivity of dietary polyphenols: the role of metabolites. Crit Rev Food Sci Nutr. (2020) 60:626–59. 10.1080/10408398.2018.1546669 30614249

[220] Yang G Ge S Singh R Basu S Shatzer K Zen M. Glucuronidation: driving factors and their impact on glucuronide disposition. Drug Metab Rev. (2017) 49:105–38. 10.1080/03602532.2017.1293682 28266877 PMC7660525

[221] Wu B Kulkarni K Basu S Zhang S Hu M. First-pass metabolism via UDP-glucuronosyltransferase: a barrier to oral bioavailability of phenolics. J Pharm Sci. (2011) 100:3655–81. 10.1002/jps.22568 21484808 PMC3409645

[222] Hu M. Commentary: bioavailability of flavonoids and polyphenols: call to arms. Mol Pharm. (2007) 4:803–6. 10.1021/mp7001363 18052085 PMC2551754

[223] Basar AO Prieto C Durand E Villeneuve P Sasmazel HT Lagaron J. Encapsulation of β-carotene by emulsion electrospraying using deep eutectic solvents. Molecules. (2020) 25:981. 10.3390/molecules25040981 32098315 PMC7070406

[224] Li X Xin Y Mo Y Marozik P He T Guo H. The bioavailability and biological activities of phytosterols as modulators of cholesterol metabolism. Molecules. (2022) 27:523. 10.3390/molecules27020523 35056839 PMC8781140

[225] Salehi B Quispe C Sharifi-Rad J Cruz-Martins N Nigam M Mishra AP. Phytosterols: from preclinical evidence to potential clinical applications. Front Pharmacol. (2020) 11:599959. 10.3389/fphar.2020.599959 33519459 PMC7841260

[226] Babadi D Dadashzadeh S Osouli M Daryabari MS Haeri A. Nanoformulation strategies for improving intestinal permeability of drugs: A more precise look at permeability assessment methods and pharmacokinetic properties changes. J Control Release. (2020) 321:669–709. 10.1016/j.jconrel.2020.02.041 32112856

[227] Singh H. Nanotechnology applications in functional foods; opportunities and challenges. Prev Nutr Food Sci. (2016) 21:1–8. 10.3746/pnf.2016.21.1.1 27069899 PMC4827628

[228] Peng X McClements DJ Liu X Liu F. EGCG-based nanoparticles: synthesis, properties, and applications. Crit Rev Food Sci Nutr. (2025) 65:2177–98. 10.1080/10408398.2024.2328184 38520117

[229] Siddiqui SA Bahmid NA Taha A Khalifa I Khan S Rostamabadi H. Recent advances in food applications of phenolic-loaded micro/nanodelivery systems. Crit Rev Food Sci Nutr. (2023) 63:8939–59. 10.1080/10408398.2022.2056870 35426751

[230] Svigelj R Dossi N Grazioli C Toniolo R. Deep eutectic solvents (DESs) and their application in biosensor development. Sensors. (2021) 21:4263. 10.3390/s21134263 34206344 PMC8271379

[231] Mussagy CU Hucke HU Ramos NF Ribeiro HF Alves MB Mustafa A. Tailor-made solvents for microbial carotenoids recovery. Appl Microbiol Biotechnol. (2024) 108:234. 10.1007/s00253-024-13049-x 38400930 PMC10894098

[232] Liu X Luo T Lin X Fei T Wang L. Deep eutectic solvents-synergistic ultrasonic-assisted extraction of polyphenols from raspberry (*Rubus idaeus* L.): Optimization, mechanisms, and in vitro and cellular antioxidant activity. Food Chem. (2025) 480:143918. 10.1016/j.foodchem.2025.143918 40117811

[233] Ferreira C Sarraguça M. A comprehensive review on deep eutectic solvents and its use to extract bioactive compounds of pharmaceutical interest. Pharmaceuticals. (2024) 17:124. 10.3390/ph17010124 38256957 PMC10820243

[234] Li Y Hu K Huang C Hu Y Ji H Liu S. Improvement of solubility, stability and antioxidant activity of carotenoids using deep eutectic solvent-based microemulsions. Colloids Surf B Biointerfaces. (2022) 217:112591. 10.1016/j.colsurfb.2022.112591 35679734

[235] Cherukuvada S Nangia A. Eutectics as improved pharmaceutical materials: design, properties and characterization. Chem Commun. (2014) 50:906–23. 10.1039/C3CC47521B 24322207

[236] Chettri A Subba A Singh GP Bag PP. Pharmaceutical co-crystals: A green way to enhance drug stability and solubility for improved therapeutic efficacy. J Pharm Pharmacol. (2024) 76:1–12. 10.1093/jpp/rgad097 37934904

[237] Smith AJ Kavuru P Wojtas L Zaworotko MJ Shytle RD. Cocrystals of quercetin with improved solubility and oral bioavailability. Mol Pharm. (2011) 8:1867–76. 10.1021/mp200209j 21846121

[238] Nyamba I Sombié CB Yabré M Zimé-Diawara H Yaméogo J Ouédraogo S. Pharmaceutical approaches for enhancing solubility and oral bioavailability of poorly soluble drugs. Eur J Pharm Biopharm. (2024) 204:114513. 10.1016/j.ejpb.2024.114513 39313163

[239] Poli G Bologna E Saguy IS. Possible interactions between selected food processing and medications. Front Nutr. (2024) 11:1380010. 10.3389/fnut.2024.1380010 38680533 PMC11045975

[240] James A Wang K Wang Y. Therapeutic activity of green tea epigallocatechin-3-gallate on metabolic diseases and non-alcoholic fatty liver diseases: the current updates. Nutrients. (2023) 15:3022. 10.3390/nu15133022 37447347 PMC10346988

[241] Zhao T Li C Wang S Song X. Green tea (*Camellia sinensis*): a review of its phytochemistry, pharmacology, and toxicology. Molecules. (2022) 27:3909. 10.3390/molecules27123909 35745040 PMC9231383

[242] Chainani-Wu N. Safety and anti-inflammatory activity of curcumin: a component of tumeric (*Curcuma longa*). J Altern Complement Med. (2003) 9:161–8. 10.1089/107555303321223035 12676044

[243] Hewlings SJ Kalman DS. Curcumin: a review of its effects on human health. Foods. (2017) 6:92. 10.3390/foods6100092 29065496 PMC5664031

[244] Kocaadam B Sanlier N. Curcumin, an active component of turmeric (*Curcuma longa*), and its effects on health. Crit Rev Food Sci Nutr. (2017) 57:2889–95. 10.1080/10408398.2015.1077195 26528921

[245] Acosta L Byham-Gray L Kurzer M Samavat H. Hepatotoxicity with high-dose green tea extract: effect of catechol-o-methyltransferase and uridine5'-diphospho-glucuronosyltransferase 1A4 genotypes. J Diet Suppl. (2023) 20:850–69. 10.1080/19390211.2022.2128501 36178169 PMC10060436

[246] Kordiak J Bielec F Jabłoński S Pastuszak-Lewandoska D. Role of beta-carotene in lung cancer primary chemoprevention: a systematic review with meta-analysis and meta-regression. Nutrients. (2022) 14:1361. 10.3390/nu14071361 35405977 PMC9003277

[247] Javaid M Kadhim K Bawamia B Cartlidge T Farag M Alkhalil M. Bleeding risk in patients receiving omega-3 polyunsaturated fatty acids: a systematic review and meta-analysis of randomized clinical trials. J Am Heart Assoc. (2024) 13:e032390. 10.1161/JAHA.123.032390 38742535 PMC11179820

[248] Golanski J Szymanska P Rozalski M. Effects of omega-3 polyunsaturated fatty acids and their metabolites on haemostasis-current perspectives in cardiovascular disease. Int J Mol Sci. (2021) 22:2394. 10.3390/ijms22052394 33673634 PMC7957531

[249] Liu S Liu J He L Liu L Cheng B Zhou F. A comprehensive review on the benefits and problems of curcumin with respect to human health. Molecules. (2022) 27:4400. 10.3390/molecules27144400 35889273 PMC9319031

[250] Binns CW Lee MK Lee AH. Problems and prospects: public health regulation of dietary supplements. Annu Rev Public Health. (2018) 39:403–20. 10.1146/annurev-publhealth-040617-013638 29272167

[251] Intrasook J Tsusaka TW Anal AK. Trends and current food safety regulations and policies for functional foods and beverages containing botanicals. J Food Drug Anal. (2024) 32:112–39. 10.38212/2224-6614.3499 38934687 PMC11210467

[252] Ravanfar R Tamaddon AM Niakousari M Moein MR. Preservation of anthocyanins in solid lipid nanoparticles: optimization of a microemulsion dilution method using the Placket-Burman and Box-Behnken designs. Food Chem. (2016) 199:573–80. 10.1016/j.foodchem.2015.12.061 26776010

[253] Han C Yang C Li X Liu E Meng X Liu B. DHA loaded nanoliposomes stabilized by β-sitosterol: Preparation, characterization and release *in vitro* and *vivo*. *Food Chem*. (2022) 368:130859. 10.1016/j.foodchem.2021.130859 34425339

[254] Otchere E McKay BM English MM Aryee ANA. Current trends in nano-delivery systems for functional foods: a systematic review. PeerJ. (2023) 11:e14980. 10.7717/peerj.14980 36949757 PMC10026715

[255] Chandrasekaran P Sivaraman G Rasala S Sethuraman MG Kotla NG Rochev Y. Quercetin conjugated fluorescent nitrogen-doped carbon dots for targeted cancer therapy application. Soft Matter. (2022) 18:5645–53. 10.1039/D2SM00747A 35861218

[256] Li B Shao H Gao L Li H Sheng H Zhu L. Nano-drug co-delivery system of natural active ingredients and chemotherapy drugs for cancer treatment: a review. Drug Deliv. (2022) 29:2130–61. 10.1080/10717544.2022.2094498 35815678 PMC9275501

[257] Thompson HJ Lutsiv T McGinley JN Hussan H Playdon MC. Dietary oncopharmacognosy as a crosswalk between precision oncology and precision nutrition. Nutrients. (2023) 15:2219. 10.3390/nu15092219 37432381 PMC10180692

[258] Chunarkar-Patil P Kaleem M Mishra R Ray S Ahmad A Verma D. Anticancer drug discovery based on natural products: from computational approaches to clinical studies. Biomedicines. (2024) 12:201. 10.3390/biomedicines12010201 38255306 PMC10813144

